# Exploring Attitudes Toward “Sugar Relationships” Across 87 Countries: A Global Perspective on Exchanges of Resources for Sex and Companionship

**DOI:** 10.1007/s10508-023-02724-1

**Published:** 2023-12-21

**Authors:** Norbert Meskó, Marta Kowal, András Láng, Ferenc Kocsor, Szabolcs A. Bandi, Adam Putz, Piotr Sorokowski, David A. Frederick, Felipe E. García, Leonardo A. Aguilar, Anna Studzinska, Chee-Seng Tan, Biljana Gjoneska, Taciano L. Milfont, Merve Topcu Bulut, Dmitry Grigoryev, Toivo Aavik, Mahmoud Boussena, Alan D. A. Mattiassi, Reza Afhami, Rizwana Amin, Roberto Baiocco, Hamdaoui Brahim, Ali R. Can, Joao Carneiro, Hakan Çetinkaya, Dimitri Chubinidze, Eliane Deschrijver, Yahya Don, Dmitrii Dubrov, Izzet Duyar, Marija Jovic, Julia A. Kamburidis, Farah Khan, Hareesol Khun-Inkeeree, Maida Koso-Drljevic, David Lacko, Karlijn Massar, Mara Morelli, Jean C. Natividade, Ellen K. Nyhus, Ju Hee Park, Farid Pazhoohi, Ekaterine Pirtskhalava, Koen Ponnet, Pavol Prokop, Dušana Šakan, Singha Tulyakul, Austin H. Wang, Sibele D. Aquino, Derya D. Atamtürk, Nana Burduli, Antonio Chirumbolo, Seda Dural, Edgardo Etchezahar, Nasim Ghahraman Moharrampour, Balazs Aczel, Luca Kozma, Samuel Lins, Efisio Manunta, Tiago Marot, Moises Mebarak, Kirill G. Miroshnik, Katarina Misetic, Marietta Papadatou-Pastou, Bence Bakos, Fatima Zahra Sahli, Sangeeta Singh, Çağlar Solak, Tatiana Volkodav, Anna Wlodarczyk, Grace Akello, Marios Argyrides, Ogeday Çoker, Katarzyna Galasinska, Talía Gómez Yepes, Aleksander Kobylarek, Miguel Landa-Blanco, Marlon Mayorga, Barış Özener, Ma. Criselda T. Pacquing, Marc Eric S. Reyes, Ayşegül Şahin, William Tamayo-Agudelo, Gulmira Topanova, Ezgi Toplu-Demirtaş, Belgüzar N. Türkan, Marcos Zumárraga-Espinosa, Simone Grassini, Jan Antfolk, Clément Cornec, Katarzyna Pisanski, Sabrina Stöckli, Stephanie Josephine Eder, Hyemin Han

**Affiliations:** 1 Institute of Psychology, Faculty of Humanities and Social Sciences, University of Pécs, Pecs, 7624 Hungary; 2 IDN Human Being Lab, University of Wrocław, Wrocław, Poland; 3 Institute of Psychology, University of Wrocław, Wrocław, Poland; 4 Crean College of Health and Behavioral Sciences, Chapman University, Orange, CA USA; 5 Departamento de Psiquiatría y Salud Mental, Facultad de Medicina, Universidad de Concepción, Concepción, Chile; 6 School of Psychology, Central University of Venezuela, Caracas, Venezuela; 7 Departament of Humanities, Icam, Toulouse, France; 8 Department of Psychology and Counselling, Universiti Tunku Abdul Rahman, Kampar, Malaysia; 9 Macedonian Academy of Sciences and Arts, Skopje, North Macedonia; 10 School of Psychology, University of Waikato, Tauranga, New Zealand; 11 Clinical Addiction Research Unit, Lund University, Malmö, Sweden; 12 Center for Sociocultural Research, HSE University, Moscow, Russian Federation; 13 Institute of Psychology, University of Tartu, Tartu, Estonia; 14 Departement of Psychology and Educational Sciences, Mohamed Lamine Debaghine, University Setif2, Setif, Algeria; 15 Department of Education, Languages, Interculture, Literatures and Psychology, University of Florence, Florence, Italy; 16 Department of Art Studies, Tarbiat Modares University, Tehran, Iran; 17 Department of Professional Psychology, Bahria University, Islamabad, Pakistan; 18 Department of Developmental and Social Psychology, Sapienza University of Rome, Rome, Italy; 19 Idepartment of Sociologie, University of Ibn Tofail, Kenitra, Morocco; 20 Department of Anthropology, Hatay Mustafa Kemal University, Hatay, Turkey; 21 Department of Social Psychology, University of Porto, Porto, Portugal; 22 Department of Psychology, Yaşar University, İzmir, Turkey; 23 Psychological Set Research and Correction Center, Tbilisi State Medical University, Tbilisi, Georgia; 24 Department of Experimental Psychology, Ghent University, Ghent, Belgium; 25 School of Psychology, University of New South Wales, Sydney, Australia; 26 School of Psychology, University of Sydney, Sydney, Australia; 27 School of Education, Universiti Utara Malaysia, Kedah, Malaysia; 28 HSE University, Moscow, Russian Federation; 29 Department of Anthropology, Istanbul University, Istanbul, Turkey; 30 Department of Marketing Management and Public Relations, Faculty of Organizational Sciences, University of Belgrade, Belgrade, Serbia; 31 Department of General, Experimental and Genetic Psychology, Sofia University, Sofia, Bulgaria; 32 Institute of Education & Research, Women University Mardan, Mardan, Pakistan; 33 Psychology and Counselling, Prince of Songkla University, Pattani, Thailand; 34 Department of Psychology, University of Sarajevo, Sarajevo, Bosnia and Herzegovina; 35 Interdisciplinary Research Team on Internet and Society, Faculty of Social Studies, Masaryk University, Brno, Czech Republic; 36 Work and Social Psychology, Maastricht University, Maastricht, The Netherlands; 37 Department of Dynamic and Clinical Psychology, and Health Studies, Sapienza University of Rome, Rome, Italy; 38 Department of Psychology, Pontifical Catholic University of Rio de Janeiro, Rio de Janeiro, Brazil; 39 Department of Management, University of Agder, Kristiansand, Norway; 40 Department of Child and Family Studies, Yonsei University, Seoul, Korea; 41 Department of Psychology, University of British Columbia, Vancouver, Canada; 42 Department of Psychology, Ivane Javakhishvili Tbilisi State University, Tbilisi, Georgia; 43 Faculty of Social Sciences, Imec-Mict-Ghent University, Ghent, Belgium; 44 Department of Environmental Ecology and Landscape Management, Comenius University, Bratislava, Slovakia; 45 Institute of Zoology, Slovak Academy of Sciences, Bratislava, Slovakia; 46 Department of Psychology, Faculty of Legal and Business Studies Dr Lazar Vrkatić, Union University, Novi Sad, Serbia; 47 Department of Health and Physical Education, Thaksin University, Songkhla, Thailand; 48 Political Science, University of Nevada, Las Vegas, Las Vegas, NV USA; 49 Department of Psychology, University of Georgia, Tbilisi, Georgia; 50 Department of Psychology, Sapienza University of Rome, Rome, Italy; 51 Department of Psychology, Izmir University of Economics, İzmir, Turkey; 52 Education Universidad Internacional de Valencia, Valencia, Spain; 53 Ciipme Conicet, Buenos Aires, Argentina; 54 School of Psychological Sciences, University of Melbourne, Melbourne, Australia; 55 Institute of Psychology, ELTE Eötvös Loránd University, Budapest, Hungary; 56 Division of Psychology, School of Education and Social Sciences, University of the West of Scotland, Paisley, Scotland, UK; 57 Departament of Psychology, University of Porto, Porto, Portugal; 58 CLLE, Université de Toulouse, Toulouse, France; 59 Department of Administration, Getúlio Vargas Foundation, Rio de Janeiro, Brazil; 60 Department of Psychology, Universidad del Norte, Puerto Colombia, Colombia; 61 Department of Psychology, Saint Petersburg State University, Saint Petersburg, Russian Federation; 62 Department of Primary Education, National and Kapodistrian University of Athens, Athens, Greece; 63 ELTE Eötvös Loránd University, Budapest, Hungary; 64 Institute of Sports Professions, Ibn Tofail University, Kenitra, Morocco; 65 Department of Psychology, Manisa Celal Bayar University, Manisa, Turkey; 66 Department of Pedagogy and Psychology, Kuban State University, Krasnodar, Russian Federation; 67 Escuela de Psicología, Universidad Católica del Norte, Antofagasta, Chile; 68 Mental Health, Gulu University, Gulu, Uganda; 69 Department of Psychology, Neapolis University Pafos, Paphos, Cyprus; 70 Department of Psychology, Pamukkale University, Denizli, Turkey; 71 Department of Psychology, Center For Research On Biological Basis of Social Behavior, SWPS University of Social Sciences and Humanities, Warsaw, Poland; 72 Department of Education, International University of Valencia, Valencia, Spain; 73 Institute of Pedagogy, University of Wrocław, Wrocław, Poland; 74 School of Psychological Sciences, National Autonomous University of Honduras, Tegucigalpa, Honduras; 75 Escuela de Psicología, Pontificia Universidad Católica del Ecuador-Ambato, Ambato, Ecuador; 76 Department of Psychology, University of Santo Tomas, Manila, Philippines; 77 Faculty of Psychology, Universidad Cooperativa de Colombia, Medellín, Colombia; 78 Department of Theoretical and Practical Psychology, Kazakh National Women’s Pedagogical University, Almaty, Kazakhstan; 79 Department of Psychological Counseling and Guidance, Mef University, Istanbul, Turkey; 80 Carrera de Psicología, Universidad Politécnica Salesiana, Quito, Ecuador; 81 Department of Psychosocial Science, University of Bergen, Bergen, Norway; 82 Cognitive and Behavioral Neuroscience Lab, University of Stavanger, Stavanger, Norway; 83 Faculty of Arts, Psychology and Theology, Åbo Akademi University, Turku, Finland; 84 ENES Bioacoustics Research Lab, CRNL, CNRS, Insern, University of Saint-Etienne, Saint-Etienne, France; 85 Department of Business Administration, University of Zurich, Zurich, Switzerland; 86 Department of Neurosciences and Developmental Biology, University of Vienna, Vienna, Austria; 87 Educational Psychology Program, University of Alabama, Tuscaloosa, AL USA

**Keywords:** Resources for sex, Sugar relationships, Cross-cultural comparison, Human mating

## Abstract

The current study investigates attitudes toward one form of sex for resources: the so-called sugar relationships, which often involve exchanges of resources for sex and/or companionship. The present study examined associations among attitudes toward sugar relationships and relevant variables (e.g., sex, sociosexuality, gender inequality, parasitic exposure) in 69,924 participants across 87 countries. Two self-report measures of Acceptance of Sugar Relationships (ASR) developed for younger companion providers (ASR-YWMS) and older resource providers (ASR-OMWS) were translated into 37 languages. We tested cross-sex and cross-linguistic construct equivalence, cross-cultural invariance in sex differences, and the importance of the hypothetical predictors of ASR. Both measures showed adequate psychometric properties in all languages (except the Persian version of ASR-YWMS). Results partially supported our hypotheses and were consistent with previous theoretical considerations and empirical evidence on human mating. For example, at the individual level, sociosexual orientation, traditional gender roles, and pathogen prevalence were significant predictors of both ASR-YWMS and ASR-OMWS. At the country level, gender inequality and parasite stress positively predicted the ASR-YWMS. However, being a woman negatively predicted the ASR-OMWS, but positively predicted the ASR-YWMS. At country-level, ingroup favoritism and parasite stress positively predicted the ASR-OMWS. Furthermore, significant cross-subregional differences were found in the openness to sugar relationships (both ASR-YWMS and ASR-OMWS scores) across subregions. Finally, significant differences were found between ASR-YWMS and ASR-OMWS when compared in each subregion. The ASR-YWMS was significantly higher than the ASR-OMWS in all subregions, except for Northern Africa and Western Asia.

**Supplementary Information:**

The online version contains supplementary material available at 10.1007/s10508-023-02724-1.

## Introduction

Sex for resources has been common for centuries (e.g., concubine, mistress, paramour; Murdock, 1952; Nelson, 1993; Ringdal, 2007; Sanger, 1858; Scott, 2014). Sugar relationships are one modern version of transactional relationships that include sexual activity and/or companionship. In a sugar relationship, a man with resources (*sugar daddy*) or, less frequently, a woman with resources (*sugar mama*) provides material compensation (money or other assets) for the sexual companionship offered by a partner who is typically younger in age (*sugar baby* or, less frequently, *sugar boy*; Nayar, 2017).

In recent years, the nature of sugar relationships has been addressed in several different disciplinary approaches (Meskó et al., 2021). Sagar et al. (2016) adopt a sociological perspective on university students’ motives for working in the sex industry. Mixon (2019) proposes an economic approach to sugar relationships, in which the concept of human capital investment explains the relatively high proportion of young female university students enrolled in expensive programs among the users of specialized dating sites. From a feminist perspective, Cordero (2015) points out the importance of negotiating power and agency by prospective partners in a sugar relationship. Other authors focus on the legal and ethical aspects of sugar relationships. Miller (2011), Motyl (2012), and Motz (2014) question whether sugar relationships should be legally defined as a form of sex work, considering that in countries where sex work (and active support for sex work) is banned, no comparable legal provision restricts the access to web services designed for managing sugar relationships. Ernst et al. (2021) focus on the prejudice, stigmatization, and social exclusion targeted at university students involved in the sex industry, which hinders these students in seeking and receiving emotional support from their social environment.

Wade (2009), the founder and CEO of *Seeking Arrangement* (the US sugar babies dating site), defines sugar relationships as an arrangement where one person provides intimacy, companionship, or other forms of attention in return for personal gain such as financial support or professional advancement. Scull (2020) states that this definition is more inclusive and representative of the diverse range of sugar relationships that exist as it encompasses those that do not involve sex or age disparities. This definition is preferable to academic ones as it is more inclusive and represents the wide range of sugar relationships that exist.

Exchanging sex for resources between young attractive partners and older partners with resources occurs in many present-day societies. The term transactional sex is mostly used by academics in reference to relationships in African countries to describe trades of sexual accessibility for resources (e.g., Choudhry et al., 2014; Masvawure, 2010; Stoebenau et al., 2016; Wamoyi et al., 2010). The term *compensated date* (Chu, 2018) is typically used in Asia and Eastern Europe, including Hong Kong (Lee & Shek, 2013), Japan (McLellan, 2013), Moscow, Kyiv, and Minsk (Swader & Vorobeva, 2015). Finally, the term *sugar relationship* is most common in North America and Europe (e.g., Birkás et al., 2020; Ipolyi et al., 2021; Láng et al., 2021; Scull, 2022; Upadhyay, 2021). In this research, we will examine sugar relationships as a specific manifestation of the broader phenomenon of “sex for resources” transactions, alongside other known variations of this phenomenon.

Although little is known about the inner nature of sugar relationships, age difference is one of the fundamental and immanent components of this type of transactional relationship. The available data suggest that sugar relationships, unlike prostitution, cannot be seen as a “purely” business transaction by the participants. In sugar relationships, the older party often shares resources related to social capital in addition to material resources (skills, network, know-how, etc.; Ojebode et al., 2010) with the younger party. This age gap is an evolutionarily well-understood part of the human mating strategy (Conroy-Beam & Buss, 2019). Therefore, we considered it important to emphasize the importance of age in the present study.

In a qualitative study of Swedish sugar daddies, men often emphasized the desire for the presence of some degree of emotional intimacy and mutual enjoyment of the relationship and sexual interactions, in addition to the economic transaction. The interactions may include initial dates to facilitate these feelings. Furthermore, to create this atmosphere, the financial transaction is often framed as a “gift” rather than as a “payment for services” (Gunnarsson & Strid, 2022, 2023). This framing is captured well by one participant who described his experience of these relationships: “The women involved in [sugar dating] are more for real, you get to take part of their… real thoughts and real life, compared to being with a prostitute that you get to be with for 30 min or an hour. In some ways, it becomes more for real” (Gunnarson & Strid, 2022, p. 316).

In a sugar relationship, the partners engage in a direct sexual transaction lacking commitment and shared reproductive goals, which is possibly part of a short-term mating strategy (Anderson & Klofstad, 2012; Burtăverde & Ene, 2021; Whyte et al., 2019). In one interview study of 48 women from the United States, several common themes emerged for why they engaged in sugar relationships. The most commonly cited motivations were money (83%), followed by material items, services, activities, and other expenses (58%), followed by sexual interactions (27%) and the companionship (25%) or hope of finding love (13%), and finally mentorship and access to social networks (8%) and curiosity, boredom, or fun (8%) (Scull, 2022). A qualitative Danish study of sugar babies aged 18–30 showed that the economic motive is dominant when engaging in sugar dating, but this is rarely the only motive (Groes et al., 2021). Additional motives were need for excitement, a wish to explore own sexual boundaries, or a need for appreciation/confirmation.

To measure psychological aspects of mating and their relation to sugar relationships in a standardized way, Birkás et al. (2020) developed an attitudinal measure of young people’s openness to engage in sex for material compensation encounters (Acceptance of Sugar Relationships in Young Women and Men Scale; ASR-YWMS). Láng et al. (2021) published a complementary measure assessing older people’s openness to provide compensation for sex with young partners (Acceptance of Sugar Relationship in Older Men and Women Scale, ASR-OMWS). Birkás et al. (2020) found that young (18–28 years) participants’ positive attitude toward sugar relationships (ASR-YWMS) were positively associated with a manipulative, game playing love style (*Ludus*), self-centered sexual motivation, unrestricted sociosexual orientation, and socially aversive personality traits such as Machiavellianism or subclinical psychopathy (two components of the Dark Triad), as well as borderline personality organization. Láng et al. (2021) replicated these psychological correlates in a sample of older people (40–71 years), using the ASR-OMWS. These results were consistent with the idea that openness to sugar relationships is part of a short-term mating strategy aimed at gaining personal benefits rapidly. Birkás and Csathó (2015) found a positive correlation between the Dark Triad traits and present-oriented time perspectives, indicating that individuals with these socially aversive traits tend to prioritize short-term benefits and focus on situations that offer immediate rewards.

The present study examined several factors expected to have universal associations with attitudes toward sex-for-resources encounters. Considered here are potentially important correlates that have not been extensively explored in past research. To this end, a large multinational sample was surveyed for reliable cross-cultural differences in ASR (and, more generally, in openness to sex for resources) and for the universal predictors of these differences, taking into account possible moderators. Given the absence of directly related previous findings, the tested predictors were adopted from studies of related outcome variables such as preferred interpersonal distance (Sorokowska et al., 2017), affective interpersonal touch in close relationships (Sorokowska et al., 2021), and love experiences (Sorokowski et al., 2023). These included (1) individual-level predictors such as sex, age, sociosexual orientation, and parasite history; and (2) culture-level predictors such as collectivism, gender equality, and social welfare as measured by the Human Development Index (HDI). The sections below summarize the reasons for the focus on these individual and cultural level predictors.

In research, the distinction between individual and cultural context of predictor variables can be unclear (Leung, 1989). Individual-level predictors are directly related to the individual, which can explain differences between individuals. Cultural-level predictors are those variables that can explain why individuals belonging to a certain culture are similar to each other (e.g., have similar preferences, habits, values) and why they are different from individuals belonging to other cultural units. However, these two levels of predictors are not always sharply separated. For example, resistance to pathogens has both an individual dimension (immune system, health behavior, etc.) and a cultural dimension (prevalence of climate-specific parasites, level of health care, etc.). Thus, depending on whether we use a self-report questionnaire (individual aspect) or analyze a public database (cultural aspect), we can methodologically utilize both individual and cultural type data on the same individual (or cultural) level predictor.

### Individual-Level Predictors

#### Sex

Previous studies consistently found that men scored higher on openness toward sugar relationships than women did (e.g., Birkás et al., 2020; Ipolyi et al., 2021; Láng et al., 2021; Scull, 2022; Upadhyay, 2021). These findings are in line with the proposed sex difference in prioritization of sexual variety and access among men, who have higher reproductive potential and lower minimum obligatory biological costs of mating, versus women, who have higher obligatory biological costs and might require long-term investment from a mate more consistently than men do (e.g., Buss, 1989; Walter et al., 2020).

#### Age

Past research has found no significant associations of age with acceptance of sugar relationships among men or women (Birkás et al., 2020; Ipolyi et al., 2021; Láng et al., 2021). Older people completed the ASR-OMWS, while younger people completed the ASR-YWMS, which had partially different items. Thus, attitudes toward openness to participating in sugar relationships were not directly comparable between the two age groups using this method. To shed more light on this matter, participants in this study completed both versions.

#### Sociosexual Orientation

Simpson and Gangestad (1991) use the term sociosexuality to describe one’s willingness to engage in casual sex, particularly without emotional connection or commitment. Individuals with an unrestricted sociosexual orientation show a higher openness to casual sexual encounters (i.e., a short-term mating strategy) than those with a restricted sociosexual orientation, who are less willing to engage in uncommitted and emotionally detached sex (i.e., they pursue a long-term mating strategy; Penke & Asendorpf, 2008; Simpson & Gangestad, 1991, 1992). Schmitt (2005) conducted a 48-nation study of sociosexuality, which examines the relationship between culture and human mating strategies. He found that sociosexuality, or the willingness to engage in sexual behavior outside of a committed relationship, varied widely across cultures. This is an important finding as it suggests that cultural factors might play a significant role in shaping human mating strategies. Birkás et al. (2020) found that the higher young participants scored on the ASR-YWMS, the more unrestricted sociosexual orientation they reported (*r* = .52), while Láng et al. (2021) observed a comparable positive association between scores on the ASR-OMWS and unrestricted sociosexuality reported by older participants (*r* = .55).

#### Parasite History

The external signs of pathogens carried by individuals have been an important source of information to observers since early stages of human evolution (Ewald, 1994). The ability to detect the relevant cues of an infection, combined with motivational systems such as disgust or fear when exposed to these cues provided an adaptive response to avoid close contact with the contagious person, would have likely enhanced survival and reproductive success (Anderson & May, 1991). The highest risk for a human group is posed by highly virulent non-endemic (i.e., previously not common) pathogens (Thornhill & Fincher, 2014, 2015). In addition to the pathogens transmitted in a wide variety of social situations, sexually transmitted infection (STIs) may have exerted further selection pressure on the human mating psychology (Mackey & Immerman, 2000). The feelings of disgust and fear of infection are adaptive emotional responses potentially reducing the risk of contracting an STI (Nesse & Ellsworth, 2009; Rozin et al., 2008). Hlay et al. (2022) found that the intensity of sexual disgust and pathogen disgust were positively associated with a more restricted sociosexual orientation.

### Culture-Level Predictors

#### Individualism–Collectivism

Hofstede (1980, 1981) defines culture as the collective programming of the mind distinguishing the members of one group or category of people from others. One fundamental dimension on which cultures differ from each other is individualism–collectivism (IND–COL; Hofstede, 1980, 1981). In societies with high collectivism, people largely depend on close intragroup ties, while those living in societies with high individualism show a high preference for independence and maintain a strong sense of autonomy (Hofstede, 2001). As Nayar (2017) notes, the emergence of modern individualism (accompanied by a decline in collectivist values) shifted courtship and intimacy from the private sphere to the public sphere of recreation and consumerism. Mulvihill and Large (2019) go as far as suggesting that practices of “transactional intimacy” are replacing traditional interpersonal intimacy in individualistic societies. Based on a cross-cultural study, Schmitt (2005) suggests that cultures that value individualism and self-expression tend to have higher levels of sociosexuality. In this perspective, young people engaging in sugar relationships (sugar babies/boys) choose to pursue a financial strategy to cope with their economic and social conditions when offering their older and wealthier prospective partners (sugar daddies/mommies) companionship in return for material compensation (Nayar, 2017). Minkov et al. (2013) found that so-called personal-sexual norms (e.g., participation to sex for resources) strongly correlated with national wealth and individualistic values. The wealthy and individualistic countries scored higher on the justifiability of these behavior, suggesting that individualism is associated with freedom of personal choice in crucially important matters, such as sexual behavior.

#### Gender Equality

According to the biosocial role theory (previously known as social role theory), sex differences in human behavior primarily originate in men’s and women’s different positions in social structures rather than in evolutionary processes resulting in dispositional differences (Eagly & Wood, 1999, 2016; Wood & Eagly, 2012). This perspective assigns primary importance to gender equality, which it considers to depend on whether women can release themselves from men’s oppressive power (e.g., Coy, 2012). Consequently, gender equality is not possible as long as women submit themselves to men sexually (and otherwise). Sex for resources (e.g., sugar relationships, prostitution) is the most extreme case of sex-based oppression, since sexuality in this case is not an interaction that takes place between equal partners but a business transaction in which a man offers money to a woman in return for using her body as a means of sexual satisfaction. In an equality-oriented perspective, sex for resources results from a sexually oppressive patriarchal social order (Miller & Schwartz, 1995; Upadhyay, 2021), which assigns particular importance to differentiated sex roles. The more differentiated sex roles (i.e., common gender-related behavioral norms and beliefs) are in a society, the more intense the pressure for conforming to role expectations (Wood & Eagly, 2012). Since sex for resources is a result of sex-based oppression, it is more frequent in societies where gender inequality is more pronounced (Benoit et al., 2019). According to research by Lippa (2009), there is a positive correlation between the degree of gender equality in a country and the magnitude of gender differences in sexual desire and sociosexuality, indicating that the level of gender equality may influence the size of gender disparities in sexual behavior and human mating strategies. Additionally, Schmitt (2005) found that cultures with greater gender equality tend to have higher levels of sociosexuality, while cultures with greater gender inequality tend to have lower levels of sociosexuality.

#### Human Development Index

The HDI is a composite measure including life expectancy (closely related to access to health care and nutrition), education (mean years of schooling completed and expected years of schooling upon entering the education system), and income per capita (Stanton, 2007). Cross-national differences in HDI have been associated with a number of individual-level variables, such as social trust (Özcan & Bjørnskov, 2011; Sorokowski et al., 2023). The relative development of a society presumably impacts the motives for sexual contact between men and women (e.g., Lippa, 2009; Schmitt, 2005). Even though both women and men are receptive to many of the qualities of a potential partner (e.g., place more importance on similarity and on socially appealing personality characteristics such as intelligence, honesty, warmth; Regan et al., 2000), several empirical findings show that there are potential sex differences in sexual preferences. Men generally prefer potential opposite-sex partners who are younger than themselves and physically attractive, while women prefer partners who are older and wealthier than themselves (Buss, 1989; Conroy-Beam et al., 2015; Walter et al., 2021; Zhang et al., 2019). Although no previous studies explored the relationship between openness to sex for resources and HDI, indirect empirical evidence suggests that they should be negatively associated. Specifically, young people living in developing countries are more exposed to sexual exploitation than those living in countries at a higher level of development (e.g., Averdijk et al., 2020; Krisch et al., 2019; LoPiccalo et al., 2016).

### Research Aims and Hypotheses

The study design was informed by the basic methodological principles of cross-cultural research in psychology (see, e.g., Poortinga & Fontaine, 2022), and was part of a larger cross-cultural project (see Kowal et al., 2022, 2023). The aim of the present work was twofold. First (H1), the psychometric properties of the ASR-YWMS (Birkás et al., 2020) and the ASR-OMWS (Láng et al., 2021) were assessed with a large multinational sample. Furthermore, we tested the sex differences obtained in previous studies in both ASR scales (H2) and finally intra- and cross-subregional differences and similarities in both ASR scales (H3). According to our second aim, we (H4) explored the associations of ASR with the predictor variables.

#### Hypothesis 1

The first set of hypotheses centered around establishing the validity of the measures across cultures. When we tried to define the geographical unit of analysis, we found that the analysis by country was too fine-grained, while the analysis by 7 world regions seemed too oversimplified. Both would have made interpretation difficult. We therefore chose an intermediate path and proceeded with the analysis by subregions. The sampled regions and subregions were coded in accordance with the standard country or area codes for statistical use published by the Statistics Division of the United Nations Secretariat (1999). Subregional analysis is a common and widespread method for understanding certain socio-economic and health processes (e.g., Nishimura et al., 2022; OECD, 2015; UNICEF & WHO, 2019).

#### Hypothesis 1.1

Cross-linguistic measurement invariance.

It was expected that for each of the individual-level measures we used (ASR-YWMS; the ASR-OMWS; the Three-Item Sociosexual Orientation Inventory, SOI-3; the Gender-Equitable Men Scale, GEMS; and the Individualism–Collectivism scale), measurement invariance would be supported so that the data obtained from these measures would be suitable for further analysis.

#### Hypothesis 1.2

Cross-sexual measurement invariance.

It was expected that our research scales, the ASR-YWMS and ASR-OMWS, would measure constructs of interest in a consistent way across men and women. That is, we hypothesized sexual invariance in both measures.

#### Hypothesis 2

Cross-sexual differences of the acceptance of sugar relationships.

Based on sex differences obtained in previous analyses on Hungarian samples (men scored higher on both ASR measures; Birkás et al., 2020; Láng et al., 2021), we expected to obtain similar sex differences in the present multicultural analysis, with men scoring higher on both scales (i.e., ASR-YWMS and ASR-OMWS).

#### Hypothesis 3

Intra- and cross-subregional differences and similarities.

We hypothesized that openness to sugar relationships measured by ASR-YWMS and ASR-OMWS would be articulated differently in different subregions and that this difference is not due to the cultural variation of the instruments.

#### Hypothesis 3.1

Cross-subregional measurement invariance.

It was expected that the ASR-YWMS and ASR-OMWS are suitable for comparative analysis of subregions because the scales consistently measure constructs of interest across subregions, and thus are invariant across subregions.

#### Hypothesis 3.2

Cross-subregional differences of the acceptance of sugar relationships.

It was expected that there are differences in ASR-YWMS and ASR-OMWS scores across subregions. That is, people living in different subregions have different attitudes toward openness to sugar relationships compared to each other.

#### Hypothesis 3.3

Intra-subregional differences between the ASR-YWMS and ASR-OMWS.

It was expected that there are differences between ASR-YWMS and ASR-OMWS scores within cultures. It was also expected that the difference between ASR-YWMS and ASR-OMWS would differ between cultures.

#### Hypothesis 4

Associations between individual- and country-level predictors of the acceptance of sugar relationships.

Openness to sugar relationships was expected to be associated with the different individual and country-level predictor variables based on the theoretical introduction.

#### Hypothesis 4.1

Individual-level predictors of ASR-YWMS.

At the individual level, ASR-YWMS was expected to be associated positively with being a man, age, unrestricted sociosexual orientation (measured by the SOI-3), traditional gender roles (measured by the GEMS), individualistic values (measured by Collectivism Scale) and pathogen prevalence (measured by the nine-item Pathogen Prevalence Index).

#### Hypothesis 4.2

Country-level predictors of ASR-YWMS.

At the country level, ASR-YWMS was expected to be associated positively with gender inequality (measured by the Gender Inequality Index), and parasite stress (measured by the Country-specific zoonotic and non-zoonotic parasite stress). Furthermore, ASR-YWMS was expected to be associated negatively with HDI and collectivism (measured by the 178-nation index of Ingroup Favoritism).

#### Hypothesis 4.3

Individual-level predictors of ASR-OMWS.

At the individual level, ASR-OMWS was expected to be associated positively with being a man, age, unrestricted sociosexual orientation (measured by the SOI-3), traditional gender roles (measured by the GEMS), individualistic values (measured by Collectivism Scale), and pathogen prevalence (measured by the nine-item Pathogen Prevalence Index).

#### Hypothesis 4.4

Country-level predictors of ASR-OMWS.

At the country level, ASR-OMWS was expected to be associated positively with gender inequality (measured by the Gender Inequality Index), and parasite stress (measured by the Country-specific zoonotic and non-zoonotic parasite stress). Furthermore, ASR-OMWS was expected to be associated negatively with HDI and collectivism (measured by the 178-nation index of Ingroup Favoritism).

## Method

### Participants and Procedure

A total of 118,324 participants from 176 countries completed the survey. After excluding those participants who did not meet the inclusion criteria (passing the attention check) and those linguistic subsamples that did not reach the size of at least 100 participants, the final sample consisted of 69,924 individuals from 87 countries, who completed the survey in one of 37 languages (see details in Supplementary Materials).

The final sample included 45,509 (65.12%) women, 23,449 (33.55%) men, 665 (.91%) non-binary individuals, 264 (.38%) chose “prefer not to say,” and 37 participants who provided no data regarding their gender. Regarding sex at birth 45,745 (66.03%) were female, 23,434 (33.83%) participants’ sex were male, 96 (.14%) were intersex, and 649 did not respond. The participants were aged 18 to 90 years (*M* = 29.68, SD = 11.99, *Mdn* = 25). Regarding intimate partner relationship, 17,685 (25.32%) participants were dating someone, 9,050 (12.96%) were in a committed relationship, 14,494 (20.75%) were married, 28,626 (40.98%) single, and 2069 did not respond. Regarding employment, 18,966 (32.86%) participants were students, 3,720 (6.44%) were in full time employment, 4,522 (7.83%) were in part time employment, 4,302 (7.45%) were self-employed, 24,560 (42.55%) were unemployed, 1,652 (2.86%) were retired, and 12,202 did not respond. (See Table S1.A and Table S1.B in Supplementary Materials for demographic data by country.)

The original Hungarian survey was translated into 37 languages, and all translated versions were checked for consistency with the back-translation method (Brislin, 1970, 1983; Hambleton & De Jong, 2003; Muñiz et al., 2013; see Supplementary Materials for the detailed instructions provided for all translation teams). Data were collected online between April and August 2021. Online data collection was conducted via Qualtrics in all but four countries. Due to technical reasons, one Russian collaborator collected data using the Toloka website (a crowdsourcing platform popular in Russia), the Algerian and Moroccan participants provided data in a paper-and-pencil format, and the Iranian participants completed a Google form. The collaborators ensured that all national subsamples were heterogeneous in terms of sex, age, residence (urban vs. rural), and education. The participants were invited to share the link to the survey to their own networks on social media platforms. Approximately 6% of the obtained data were collected with the help of outsourcing companies. The brief descriptive statistics regarding the sample size, sex, and age in each language group is presented in Table S2 in Supplementary Materials for additional information.

### Transparency and Openness

We report how we determined our sample size, all data exclusions, all manipulations, and all measures in the study, and we follow Journal Article Reporting Standards (JARS; Kazak, 2018). All data, analysis code, and research materials are available at https://osf.io/prdj2/?view_only=48e348faeac344a59554ad44c7e89482. Data were analyzed using R, version 4.0.0 (R Core Team, 2020). See the section Analytic Plan for the detailed analytic methodology. This study’s design and its analysis were not pre-registered.

### Individual-Level Measures

#### Acceptance of Sugar Relationships in Young Women and Men Scale

The ASR-YWMS (Birkás et al., 2020) is a five-item scale assessing one’s willingness to engage in a sugar relationship as the younger partner (sugar baby/boy), who provides sexual companionship for the older partner in return for material compensation (gifts and/or money). Examples of items include: If it would benefit my career, I would think about engaging in a sugar relationship; I would seriously consider engaging in a sugar relationship if I thought it would help me have a better financial situation. The participants rated each item on a seven-point scale ranging from Absolutely disagree (1) to Absolutely agree (7). (See the 37-language version of the ASR-YWMS and details of internal consistency in Table S3 in Supplementary Materials.)

#### Acceptance of Sugar Relationships in Older Men and Women Scale

The ASR-OMWS (Láng et al., 2021) is a five-item scale assessing one’s willingness to engage in a sugar relationship as the older partner (sugar daddy/mommy), who provides material compensation (gifts and/or money) for the younger partner in return for sexual companionship. Examples of items include: “If it would be beneficial for my sex life or for others’ judgment of me, I would consider engaging in a sugar relationship”; “I would seriously consider engaging in a sugar relationship if that was the way to find a partner who would meet all my needs.” The participants rated each item on a five-point scale ranging from Absolutely disagree (1) to Absolutely agree (7). (See the 37-language version of the ASR-OMWS and details of internal consistency in Table S3 in Supplementary Materials.)

#### Three-Item Sociosexual Orientation Inventory (SOI-3)

The Revised Sociosexual Orientation Inventory (SOI-R; Penke & Asendorpf, 2008) is a nine-item scale assessing one’s sociosexual behavior, attitude, and desire with three items each. Since the present study only focused on the participants’ overall sociosexual orientation and due to space constraints, we only used one item of each component: Sociosexual behavior (With how many different partners have you had sexual intercourse without having an interest in a long-term committed relationship with this person?); Sociosexual attitude (I can imagine myself being comfortable and enjoying “casual” sex with different partners); and Sociosexual desire (In everyday life, how often do you have spontaneous fantasies about having sex with someone you have just met?). The participants rated each item on a nine-point scale and ranging from “1 = 0 to 9 = 20 or more” for Sociosexual behavior; ranging from “1 = Strongly disagree” to “9 = Strongly agree” for Sociosexual attitude, and “1 = Never” to “9 = At least once a day” for Sociosexual desire. Higher overall scores indicated a more unrestricted sociosexual orientation. (See details of internal consistency in Table S3 in Supplementary Materials.)

#### Gender-Equitable Men Scale

The original GEMS (Pulerwitz & Barker, 2008) is a 24-item scale assessing young men’s attitudes toward various sex-related norms. A more recently developed version (Levtov et al., 2014) comprises five subscales (Gender, Violence, Masculinity, Sexuality, and Reproductive Health), of which the Gender subscale was used in the present study to assess the male participants’ adherence to traditional gender roles. The subscale comprises three reverse-scored items: A woman’s most important role is to take care of her home and cook; Changing diapers, giving kids a bath, and feeding kids are the mother’s responsibility; A man should have the final word about decisions in his home. Each was rated on a seven-point scale ranging from Absolutely agree (1) to Absolutely disagree (7). (See details of internal consistency in Table S3 in Supplementary Materials.)

#### Personal Individualism Measured Using the Collectivism Scale

The items were consistent with the collectivism-to-individualism continuum proposed by Hofstede (1981, 2001). The scale comprises four reverse-scored items as follows: Group welfare is more important than individual rewards; Group success is more important than individual success; Being accepted by the members of the workgroup is very important; and Employees should pursue their goals only after considering the welfare of the group. After analyzing the Collectivism Scale (Wu, 2006), it became evident that the third item may not reliably represent general attitudes toward individualism because it focused specifically on a workplace context where different norms might operate. We performed an exploratory factor analysis and this item loaded onto the individualism attitudes less than did the other items (.37 as compared to .83, .87, and .50), and fell below the usually recommended criterion (i.e., below .40; Costello & Osborne, 2005). We therefore removed that item and used the remaining three items in all subsequent analyses. The participants rated each item on a seven-point scale ranging from Absolutely disagree (1) to Absolutely agree (7). Higher overall scores indicated higher individualism. (See details of internal consistency in Table S3 in Supplementary Materials.)

#### Parasite History

The 9-item Pathogen Prevalence Index (Murray & Schaller, 2010) was used to measure parasite history. The participants responded to the question, Have you ever contracted (been sick with) any of the following diseases? concerning each of nine infectious diseases, including leishmanias, schistosomes, trypanosomes, leprosy, malaria, typhus, filariae, dengue, and tuberculosis, for which Murray and Schaller (2010) calculated the index at a regional level. The response alternatives were Never (0), Once (1), and More than once (2). Higher overall scores indicated more frequent past experiences with parasites. (See details of internal consistency in Table S3 in Supplementary Materials.)

### Country-Level Measures

#### Gender Inequality Index and Human Development Index

Gender Inequality Index (GII) and the HDI of each sampled country (for data on the HDI—Human Development Report, 2021/2022; United Nations Development Programme, 2022) were used for analyses.

#### National Collectivism

The 178-nation index of Ingroup Favoritism from Van de Vliert (2011) was used as a proxy for the collectivism score for each country.

#### Parasite Load

Country-specific zoonotic and non-zoonotic parasite stress in a given region were analyzed using data reported by Fincher and Thornhill (2012).

### Analysis Plans

#### Cross-Linguistic Measurement Invariance Test and Measurement Alignment

In the present study, participants were presented with different survey forms depending on their language. Because we intended to examine the relationship between variables with data collected across different languages, we needed to assure that the survey forms in different languages were measuring the constructs of interest in a consistent manner, particularly when the constructs were measured in terms of latent variables requiring validity checks (Fischer & Karl, 2019).

To be able to assure the requirement for cross-language data analysis, we conducted a measurement invariance test to assess whether the measurement model of each scale in the survey forms was consistently valid across different language versions (Putnick & Bornstein, 2016). The measurement invariance test was performed via multigroup confirmatory factor analysis (MG-CFA) in R package, using *lavaan*. Whether measurement invariance was supported for a scale was determined by the changes in the fit quality indicators, that is, root mean square error of approximation (RMSEA), standardized root mean squared residual (SRMR), and comparative fit index (CFI), across different levels of model constraints. The least restrictive level of model invariance is configural invariance, which only assumes an equal measurement model across different groups. Metric invariance assumes equal factor loadings in addition to configural invariance. Scalar invariance additionally requires equal intercepts. Finally, the most restrictive form of invariance, residual invariance, additionally assumes equal residuals. In cross-group studies involving multiple-group comparisons of latent variables, at least scalar invariance should be supported. If scalar invariance was supported, we assumed that composite scores were possible to use for further analyses.

Whether a specific level of invariance is supported by evidence was determined by examining to what extent RMSEA, SRMR, and CFI change when an additional assumption is added while performing MG-CFA (Putnick & Bornstein, 2016). First, the first level of invariance, configural invariance, is tested by examining the fit indicators when MG-CFA is conducted. We planned to utilize the widely used criteria for this purpose, that is, RMSEA and SRMR < .08, and CFI ≥ .90 (Hu & Bentler, 1999). For metric invariance, we tested ΔRMSEA and ΔSRMR <  + .30, and ΔCFI ≥ − .01, and for scalar and residual invariance, ΔRMSEA and ΔSRMR <  + .15, and ΔCFI ≥ − .01. In all cases, as the observed responses were anchored to ordinary scales, not continuous scales, we used the WLSMV estimator to minimize the potential bias (Li, 2016).

If scalar invariance was not supported by evidence, we conducted measurement alignment to address the non-invariance issue (Tam & Milfont, 2020). Measurement alignment is a psychometric method that adjusts factor loadings and intercepts to absorb the existing non-invariance and achieve scalar invariance for cross-group analysis (Asparouhov & Muthén, 2014). Measurement alignment was performed with an R package, *sirt* (Robitzsh, 2021). After performing measurement alignment, we examined whether *R*^2^_loadings_ and *R*^2^_intercepts_, which indicate to what extent the non-invariance in factor loadings and intercepts were absorbed via alignment, respectively, were .75 or higher (Han, 2022b). When these values exceeded .75, we assumed that alignment successfully addressed the non-invariance issue.

Then, Monte Carlo simulations were performed to test whether alignment was able to absorb non-invariance in a consistent and valid manner. We generated simulation datasets with *N* = 100, 200, and 500 with the mean and variance of a scale score of interest in each group (Han et al., 2022). Then, we performed measurement alignment and then the resultant group latent means were significantly correlated with the group latent means estimated from ordinary MG-CFA. Following Han et al. (2022), we examined whether the correlation coefficient was .95 or higher (Muthén & Asparouhov, 2018). For additional information, we also examined the correlation of group variances, although it was not required to be .95 or higher. To speed up the repetitive simulation processes, we employed multiprocessing, which utilized multiple processes, as done in Han et al. (2022). Once measurement alignment was successfully completed, we calculated latent factors scores of the tested scales with adjusted factor loadings and intercepts for further analyses.

#### Comparison of Measurement Invariance Between Men and Women

To be able to compare the ASR-YWMS and ASR-OMWS across two sexes, first, we performed additional data filtering. In this process, we only extracted responses collected from participants who identified their current gender as identical to their sex at birth, and who were not sexually non-binary. Then, we conducted the measurement invariance test by employing the same procedures and criteria that were used to examine cross-linguistic invariance. If scalar invariance was supported and measurement alignment was not required, then we compared the latent mean score between groups of men and women. If scalar invariance was not supported, we conducted measurement alignment and then calculated factor scores with adjusted factor loadings and intercepts. Then, we compared the latent mean score, which was calculated with the adjusted factor scores, between the two groups.

Comparison was performed by both frequentist and Bayesian *t*-tests. Bayesian *t*-test was performed to examine whether our alternative hypothesis, which was whether there was a significant nonzero difference in the variable of interest across two different groups, was supported by evidence directly, instead of testing whether our null hypothesis should be rejected (Wagenmakers et al., 2018a). Given *p*-values are only capable of testing the null hypothesis, not the alternative hypothesis that we were interested in, and likely to lead to inflated false positives, we decided to examine Bayes Factors (BF) additionally (Wagenmakers et al., 2018b).

BFs indicate to what extent an alternative hypothesis is more favored by evidence compared with a null hypothesis. For instance, if BF = 10, it means that evidence ten times more strongly supports an alternative hypothesis versus a null hypothesis. In general, when BF ≥ 3, it is possible to assume that there is significant positive evidence supporting an alternative hypothesis. In the same vein, BF ≥ 10 implies strong evidence while BF ≥ 100 implies very strong evidence (Han, 2022a; Raftery, 1995). In the present study, Bayesian *t*-test was performed with an R package, *BayesFactor* (Morey et al., 2018). Additionally, we also calculated an effect size indicator, Cohen’s *d*.

#### Cross-Subregional Comparison of Measurement Invariance

While comparing the ASR-YWMS and ASR-OMWS across fourteen different geographical regions, we first started with the measurement invariance test and measurement alignment similar to the cross-sexual comparison. Once the measurement invariance test and measurement alignment were completed, we examined whether there was a significant difference in the ASR-YWMS and ASR-OMWS across the subregions via ANOVA. In addition to frequentist ANOVA, we also conducted Bayesian ANOVA with *BayesFactor*. The same BF criteria were used to determine whether an alternative hypothesis was supported. After performing ANOVA, we performed the post hoc test via Tukey’s honestly significant difference (HSD) test to examine which pairs of subregions demonstrated the significant difference while controlling for the family-wise error rate.

#### Intra-Subregional Comparison of Measurement Invariance

We also compared the ASR-YWMS with ASR-OMWS in each individual subregion. Because two different scales were compared, we employed a method for nonparametric comparison, the Wilcoxon test. When *p*-values were calculated, we performed the false discovery rate correction to prevent potential inflated false positives. In addition to the Wilcoxon test statistics, we also calculated Cohen’s *d* to examine the effect sizes.

#### Bayesian Multilevel Modeling

To examine the association between individual- and country-level predictors and ASR variables, we conducted Bayesian multilevel modeling. Bayesian multilevel modeling allows to explore whether one model is better supported by evidence compared with another model in terms of a BF (Han, 2022a). For each dependent variable, we compared these four models:

- Model 0: DV ~ Intercept (null model)
- Model 1: DV ~ Predictors (fixed effect only model)
- Model 2: DV ~ Predictors + (1|Country) (random intercept model)
- Model 3: DV ~ Predictors + (1+Predictors|Country) (random slope model)

Each model was estimated with *brms* package (Bürkner, 2017). In this process, the default Cauchy prior distribution, Cauchy (0, 1), and the Gaussian family were employed. In addition, for better convergence and interpretation, all variables were standardized. To calculate model BFs, we compared Model 1 versus Model 0 (BF10), Model 2 versus Model 0 (BF20), Model 3 versus Model 0 (BF30). We deemed the model with the highest model BF value as the best model (Dawson et al., 2021).

Once the best model was identified, we examined whether each predictor was significant with the BF of each predictor. For instance, in the case of age, age was hypothesized to be positively associated with ASRs. Then, we assumed:

- H0 (null hypothesis): b(age) ≤ 0
- H1 (alternative hypothesis) b(age) > 0

and, calculated BF10 indicating extent to which H1 was more strongly supported by evidence compared with H0. Once BF10 ≥ 3, we deemed that the hypothesis, H1, was positively supported by evidence.

## Results

### Cross-Linguistic Measurement Invariance Test and Measurement Alignment (H1.1)

We examined whether measurement invariance was supported for each individual-level measure that we employed so that the data acquired with such measures are suitable for further analyses. We tested whether scalar invariance, which assumes equal factor loadings and intercepts across language groups, was supported. In this process, the ASR-YWMS, ASR-OMWS, SOI-3, GEMS, and Individualism–Collectivism Scale were tested. The nine-item Pathogen Prevalence Index was not tested for its measurement invariance because in some languages, all participants provided the same response to certain items (e.g., no Dengue infections for participants responding in languages spoken in European countries).

Table 1 demonstrates the results from the measurement invariance tests. In all cases, scalar invariance was not supported, so we performed measurement alignment to address the non-invariance in the measures. In addition, the Persian version of the ASR-YWMS and Individualism–Collectivism Scale were excluded from the tests due to a convergence issue. Table 2 reports the results from measurement alignment as well as Monte Carlo simulations to examine whether the results were reliable and valid. The measurement alignment procedures were able to address the non-invariance issue successfully given more than 75% of the non-invariance in factor loadings as well as intercepts were absorbed via alignment (see *R*^2^_loadings_ and *R*^2^i_ntercepts_). Also, the results from Monte Carlo simulations support the point that the outcomes were well replicated, so measurement alignment was able to produce reliable and valid outcomes. Thus, for our main analysis, we employed factor scores calculated with the factor loadings and intercepts adjusted via measurement alignment.

**Table 1:** Cross-lingual measurement invariance tests

	RMSEA	SRMR	CFI	∆RMSEA	∆SRMR	∆CFI
*Acceptance of Sugar Relationships in Young Women and Men Scale (ASR-YWMS)*						
Configural invariance	.052	.010	.994	–	–	–
Metric invariance	.073	.029	.977	.021	.018	− .016
*Acceptance of Sugar Relationships in Older Men and Women Scale (ASR-OMWS)*						
Configural invariance	.065	.014	.990	–	–	–
Metric invariance	.084	.033	.971	.018	.019	− .019
*Three-Item Sociosexual Orientation Inventory (SOI-3)*						
Configural invariance	.000	.000	1.000	–	–	–
Metric invariance	.066	.026	.976	.066	.026	− .024
*Gender-Equitable Men Scale (GEMS)*						
Configural invariance	.000	.000	1.000	–	–	–
Metric invariance	.069	.021	.977	.069	.021	− .023
*Individualism–Collectivism Scale*						
Configural invariance	.000	.000	1.000	–	–	–
Metric invariance	.059	.021	.987	.059	.021	− .013

The Persian version of the ASR-YWMS and IND-COL were excluded due to the convergence issue

**Table 2:** Measurement alignment and Monte Carlo simulation results

			Monte Carlo Simulation											
			*N* = 100				*N* = 200				*N* = 500			
			cor (mean)		cor (var)		cor (mean)		cor (var)		cor (mean)		cor (var)	
	*R*^2^_lodings_	*R*^2^_intercepts_	*M*	SD	*M*	SD	*M*	SD	*M*	SD	*M*	SD	*M*	SD
SOI-3	.96	.98	.97	.03	.51	.15	.97	.02	.49	.13	.98	.01	.46	.12
GEMS	.99	.95	.98	.01	.89	.19	.98	.01	.94	.07	.98	.01	.95	.02
IND-COL	1.00	1.00	.97	.01	.91	.05	.97	.01	.94	.04	.97	.01	.96	.01
ASR-YWMS (by language)	.99	.99	.95	.06	.95	.04	.96	.05	.94	.04	.97	.04	.94	.03
ASR-YWMS (by subregion)	.99	1.00	.99	.00	.98	.01	1.00	.00	.98	.01	1.00	.00	.98	.01
ASR-OMWS (by language)	.99	.99	.97	.02	.96	.01	.97	.01	.96	.01	.97	.01	.95	.01
ASR-OMWS (by sex)	1.00	1.00	1.00	.00	.97	.24	1.00	.00	.98	.18	1.00	.00	1.00	.00
ASR-OMWS (by subregion)	.99	1.00	.99	.01	.97	.02	.99	.01	.97	.02	1.00	.00	.96	.02

Additionally, both the ASR-YWMS and ASR-OMWS demonstrated good reliability in terms of McDonald's ω in all languages (*ω* ≥ .80). In the cases of the SOI-3 and collectivism scales, ω values were higher than .60 in all languages indicating at least acceptable reliability. The GEMS reported acceptable reliability (≥ .60) in all languages except Arabic. In general, languages reported acceptable to good reliability (≥ .70) in the case of the Pathogen Prevalence Index; however, Arabic, Spanish, and Portuguese were exceptions (< .60). See Table S3 in Supplementary Materials for full information.

### Cross-Sexual Measurement Invariance Test of the Acceptance of Sugar Relationships Scales (H1.2)

Before conducting the cross-sexual comparison of the acceptance for sugar relationships between men and women, we tested whether our scales, the ASR-YWMS and ASR-OMWS, measured the constructs of interest across sexes in a consistent manner via the measurement invariance test. Table 3 reports the results from the measurement invariance test. The most restrictive invariance, residual invariance, which assumes the equal loadings, intercepts, and residuals, was supported in the case of the ASR-YWMS. However, scalar invariance was not supported in the ASR-OMWS, so we performed measurement alignment. As shown in Table 2, the non-invariance was successfully absorbed in a reliable and valid manner. The outcome suggests that both scales can be used for cross-language investigations after performing measurement alignment.

**Table 3:** Cross-sexual measurement invariance tests

	RMSEA	SRMR	CFI	∆RMSEA	∆SRMR	∆CFI
*Acceptance of Sugar Relationships in Young Women and Men Scale (ASR-YWMS)*						
Configural invariance	.043	.008	.996	–	–	–
Metric invariance	.035	.011	.996	− .007	.004	.000
Scalar invariance	.046	.016	.991	.010	.005	− .005
Residual invariance	.051	.023	.987	.005	.006	− .004
*Acceptance of Sugar Relationships in Older Men and Women Scale (ASR-OMWS)*						
Configural invariance	.064	.013	.990	–	–	–
Metric invariance	.041	.014	.995	− .023	.001	.004
Scalar invariance	.057	.020	.986	.016	.007	− .008

### Cross-Sexual Comparison of the Acceptance of Sugar Relationships (H2)

First, we utilized the multigroup CFA, which was already implemented while testing the measurement invariance, to compare the ASR-YWMS scores between men and women. Because scalar invariance was supported in this case, measurement alignment was not required for the cross-group comparison. When the latent mean was compared between the two groups, men reported the significantly higher ASR-YWMS mean score compared with women, *t*(45,106) = 33.06, *p* < .001, Cohen’s *d* = .27, 95% CI [.25, .29], log(BF) = 553.62.

Second, we compared the factor score of the ASR-OMWS, which was calculated with the factor loadings and intercepts adjusted via measurement alignment, because scalar invariance was not supported for this scale. The comparison result indicated that men demonstrated the significantly higher ASR-OMWS mean score compared with women, *t*(43,445) = 46.50, *p* < .001, Cohen’s *d* = .39, 95% CI [.34, .45], log(BF) = 1118.45.

### Cross-Subregional Measurement Invariance Test of the Acceptance of Sugar Relationships Scales (H3.1)

1As in the case of the cross-sexual comparison, we also conducted the measurement invariance test to assure that the scales measured the constructs of interest consistently across different subregions. Table 4 reports the results from the test of both scales. Scalar invariance was not supported for both the ASR-YWMS and ASR-OMWS. Hence, we performed measurement alignment to address the non-invariance issue. As shown in Table 2, the non-invariance was successfully absorbed in a consistent manner for both scales. For the planned cross-subregional comparison, we used the factor scores calculated with the factor loadings and intercepts adjusted via measurement alignment.

**Table 4:** Cross-subregional measurement invariance tests

	RMSEA	SRMR	CFI	∆RMSEA	∆SRMR	∆CFI
*Acceptance of Sugar Relationships in Young Women and Men Scale (ASR-YWMS)*						
Configural invariance	.048	.010	.995	–	–	–
Metric invariance	.075	.025	.978	.027	.016	− .017
*Acceptance of Sugar Relationships in Older Men and Women Scale (ASR-OMWS)*						
Configural invariance	.068	.014	.990	–	–	–
Metric invariance	.082	.030	.974	.013	.017	− .016

### Cross-Subregional Comparison of the Acceptance of Sugar Relationships (H3.2)

**Fig. 1:**
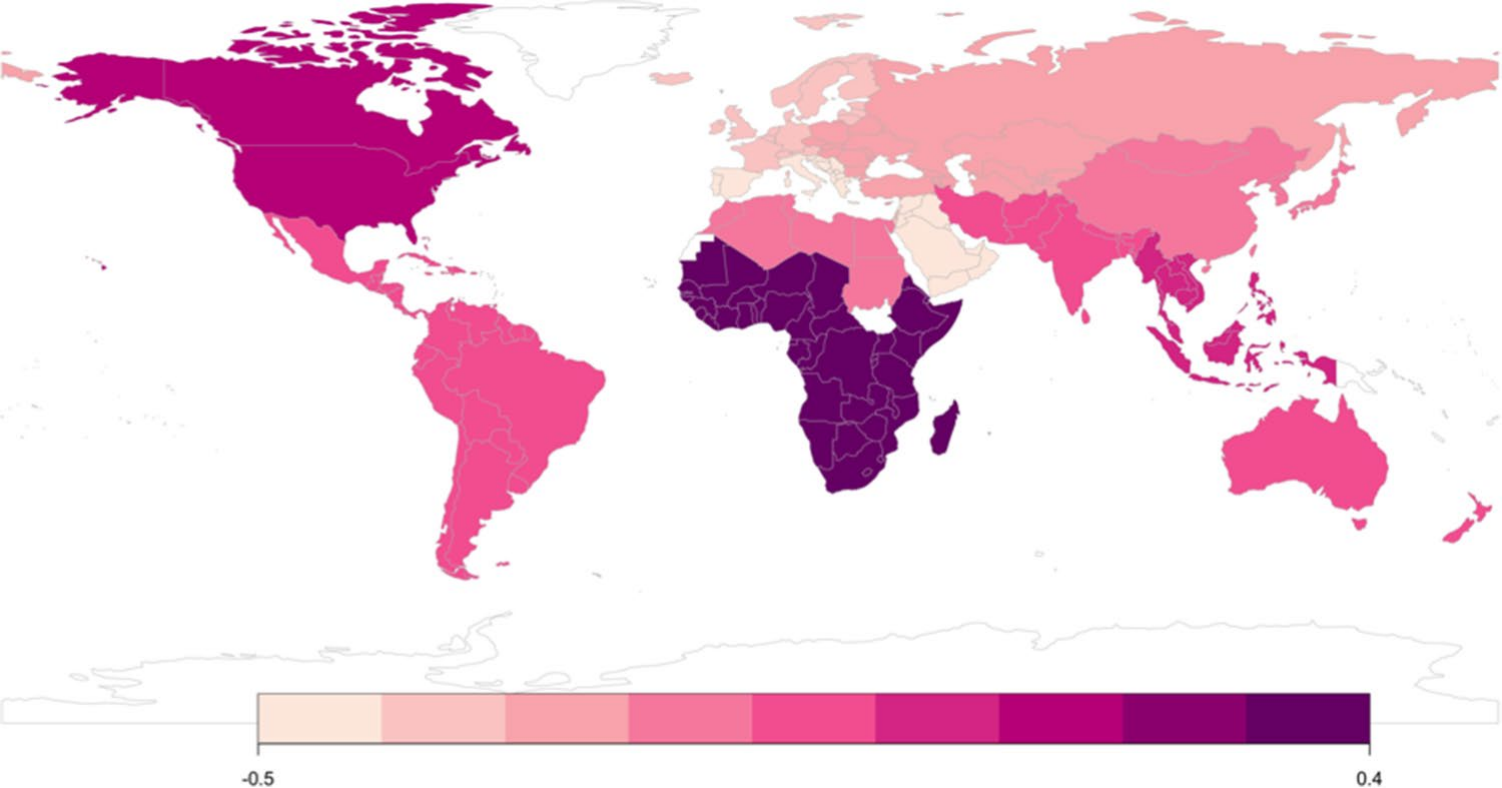
Mean Acceptance of Sugar Relationships in Young Women and Men Scale by subregion. The darker shade indicates higher scores (white areas indicate a lack of data for a given region). *Note*: Colored figures available in electronic version only

**Fig. 2:**
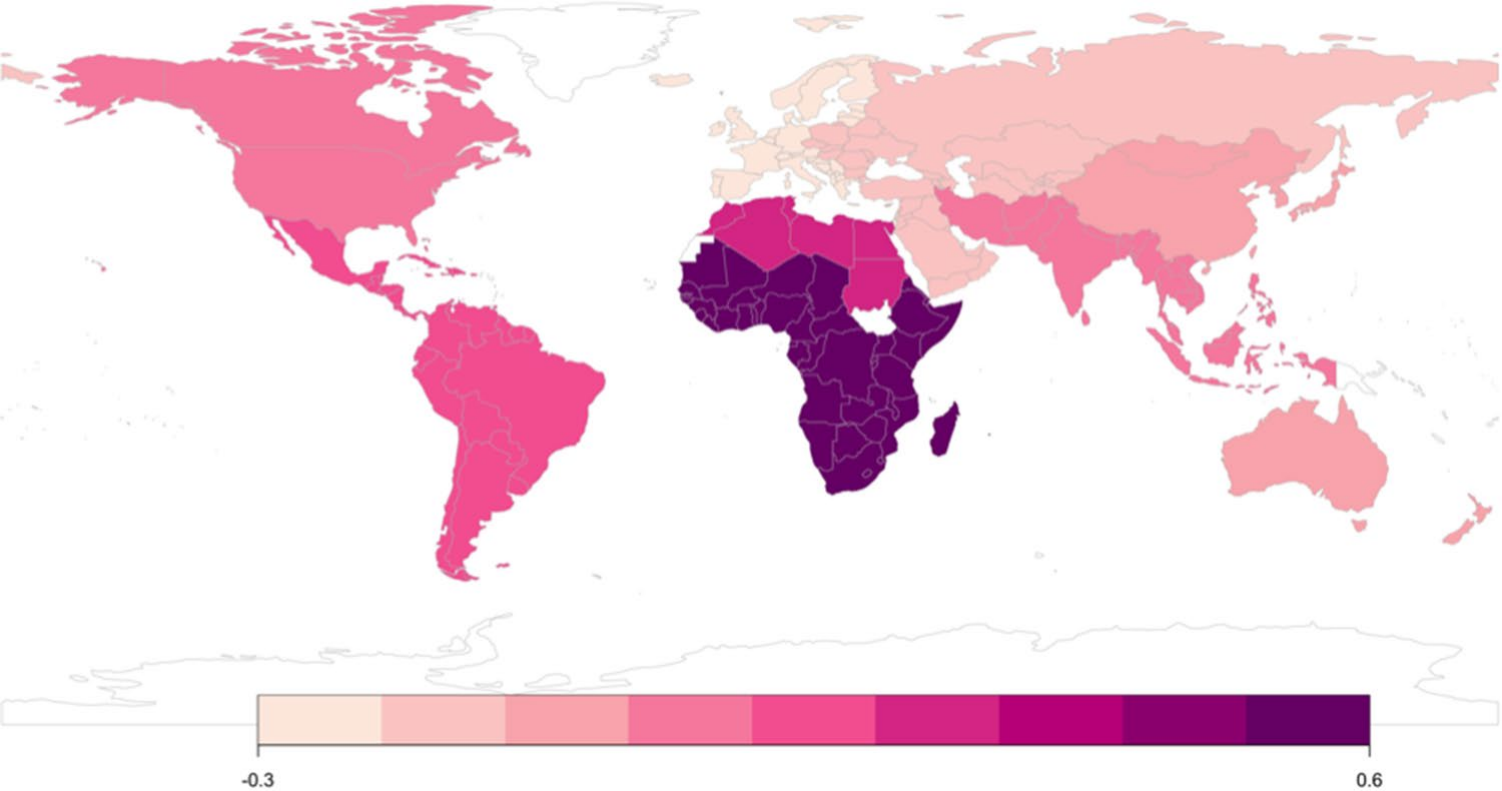
Mean Acceptance of Sugar Relationships in Older Women and Men Scale by subregion. The darker shade indicates higher scores (white areas indicate a lack of data for a given region). *Note*: Colored figures available in electronic version only

We visualized the mean score, which was calculated in terms of the latent mean score via measurement alignment, of the ASR-YWMS and ASR-OMWS in each subregion in Figs. 1 and 2, respectively.

**Fig. 3:**
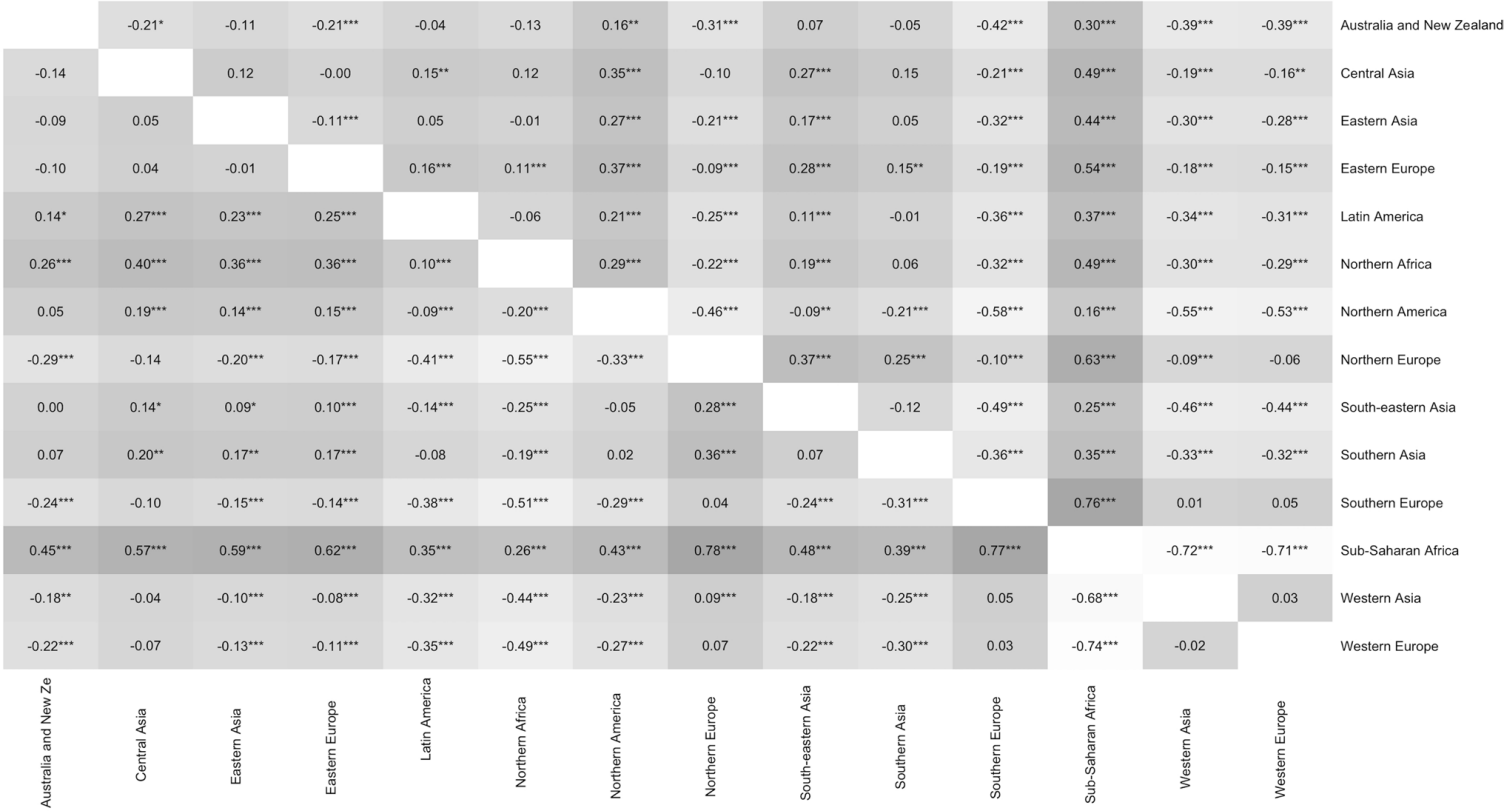
Cross-subregional differences between ASR-YWMS and ASR-OMWS. *Note*: Each cell demonstrates the Cohen’s *d* value resulting from each pair of comparison. Upper diagonal: comparisons of the ASR-YWMS. Lower diagonal: comparisons of the ASR-OWMS

ANOVA indicated that there were significant differences in the ASR-YWMS scores across different subregions, *F*(13, 10,148) = 183.49, *p* < .001, *η*^2^ = .19, log(BF) = 1,251.88. We also performed post hoc analysis via Tukey’s honestly significant different (HSD) test to control for the family-wise error rate. The result of the post hoc analysis is reported in the upper diagonal of Fig. 3.

A similar trend was found when the ASR-OMWS was examined. The result from ANOVA suggests that there were significant cross-subregional differences in the ASR-OMWS scores across subregions, *F*(13, 10,126) = 160.04, *p* < .001, *η*^2^ = .17, log(BF) = 1090.74. The result from the post hoc test is demonstrated in the lower diagonal of Fig. 3.

### Intra-Subregional Comparison Between the Acceptance of Receiving versus Giving Sugar Relationships (H3.3)

**Fig. 4:**
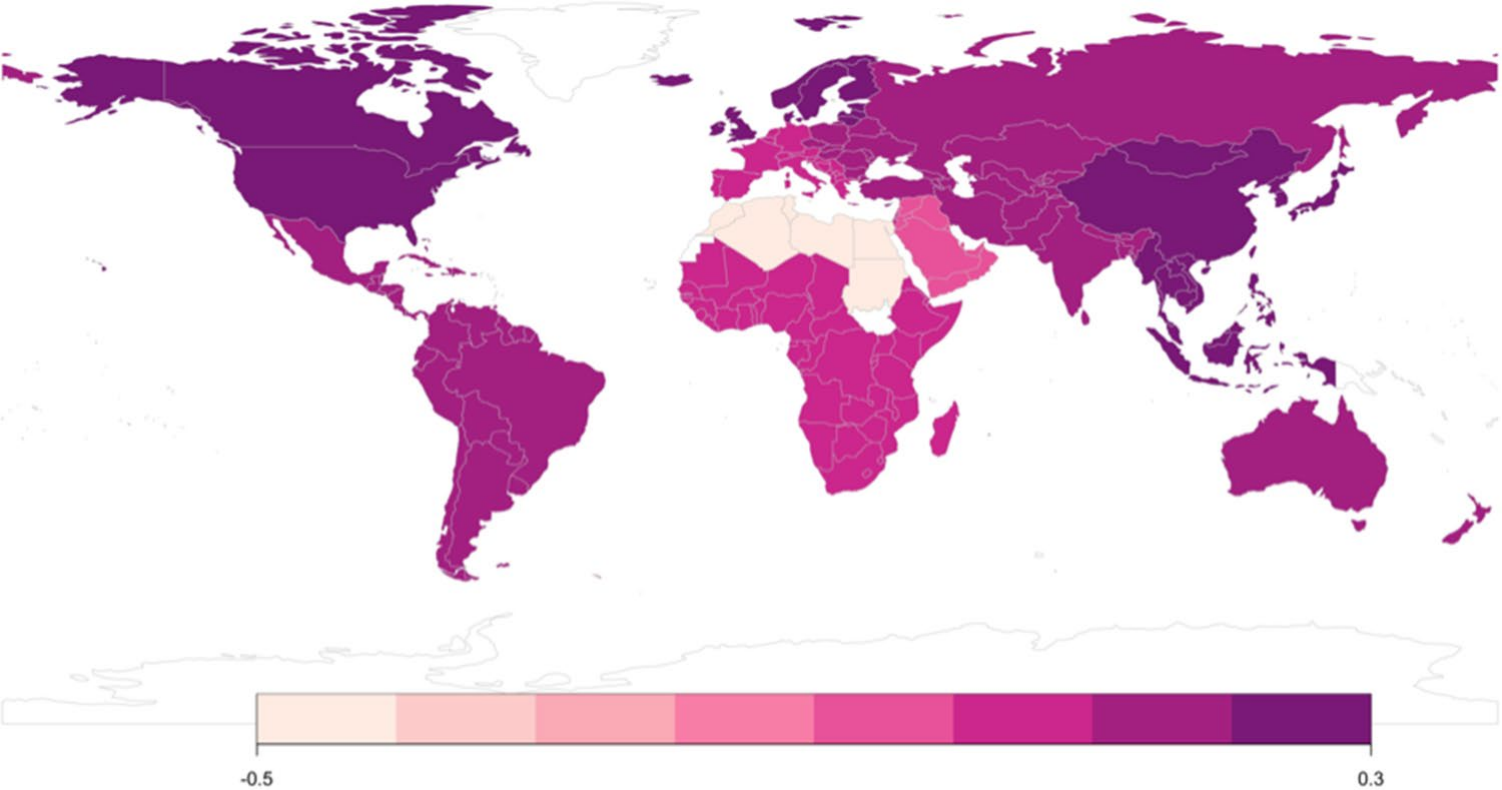
Cohen’s *d* of (ASR-YWMS—ASR-OMWS) within each subregion (white areas indicate a lack of data for a given region). *Note*: Colored figures available in electronic version only

We compared the ASR-YWMS and ASR-OMWS in each subregion. Table 5 summarizes the descriptive statistics of ASR-YWMS and ASR-OMWS, and the result of the comparison between them in each subregion. The comparison results are visualized in Fig. 4. After the false discovery rate correction, except for Northern Africa and Western Asia, the ASR-YWMS was significantly higher than the ASR-OMWS in all subregions. In Northern Africa, we found the opposite trend. There was no significant difference between the two scales in Western Asia.

**Table 5:** Results from intra-subregional comparisons of Acceptance of Sugar Relationships

Subregion	*N*	ASR-YWMS			ASR-OMWS			Wilcoxon test		
		*M*	SD	*Mdn*	*M*	SD	*Mdn*	*z*	*p* value (FDR)	Cohen's *d*
Australia and New Zealand	580	16.33	8.97	16.00	15.18	8.86	14.00	4.61	.000	.19
Central Asia	774	14.30	8.31	13.00	13.53	8.49	11.00	4.83	.000	.17
Eastern Asia	2888	15.16	7.14	15.00	14.08	7.28	13.00	12.49	.000	.23
Eastern Europe	13474	14.60	8.79	12.00	13.98	8.56	11.00	12.66	.000	.11
Latin America and the Caribbean	8103	16.50	9.18	15.00	15.96	9.27	15.00	12.44	.000	.14
Northern Africa	4395	15.05	7.60	18.00	17.03	8.97	20.00	− 32.69	.000	− .49
Northern America	4125	17.60	9.30	18.00	15.68	9.01	14.00	18.87	.000	.29
Northern Europe	4219	13.74	8.43	11.00	12.39	7.84	10.00	14.79	.000	.23
South-Eastern Asia	3325	17.09	9.36	17.00	15.43	8.84	14.00	16.44	.000	.29
Southern Asia	799	16.06	9.09	15.00	15.61	9.10	15.00	3.65	.000	.13
Southern Europe	10466	12.89	8.19	10.00	12.86	8.25	10.00	3.13	.002	.03
Sub-Saharan Africa	1829	18.99	11.00	18.00	18.76	11.20	18.00	3.69	.000	.09
Western Asia	9391	12.90	8.87	10.00	13.13	9.00	10.00	− 1.17	.242	− .01
Western Europe	4664	13.21	7.88	11.00	12.96	7.82	10.00	4.42	.000	.06

ASR-YWMS, Acceptance of Sugar Relationships in Young Women and Men Scale; ASR-OMWS, Acceptance of Sugar Relationships in Older Men and Women Scale

### Multilevel Modeling to Examine the Associations Between Individual- and Country-Level Predictors of the Acceptance of Sugar Relationships (H4)

We conducted Bayesian multilevel modeling to identify which model is the best among four candidates: the null model, fixed-effect model, random-intercept model, and random-slope model. The results of the model selection are presented in Table 6. For both the ASR-YWMS and ASR-OMWS, the random-slope model including all fixed effects, random intercepts, and random slopes was found to be best.

**Table 6:** Model comparisons for Bayesian multilevel modeling

logBFs	ASR-YMWS	ASR-OMWS
Fixed effects only versus null	10,256.52	9117.87
Random intercepts versus null	12,344.81	11,122.24
Random slopes versus null	12,644.30	11390.10

The results from Bayesian MLM are reported in Table 7.^2^ When the ASR-YWMS was examined as an outcome variable, we found several individual- and country-level predictors significantly predicting the dependent variable. At the individual level, sociosexual orientation (measured by the SOI-3), traditional gender roles (measured by the GEMS), and pathogen prevalence (measured by the nine-item Pathogen Prevalence Index) were positively associated with the ASR-YWMS.^3^ Interestingly, we found that being a woman positively predicted higher ASR-YWMS scores, while age negatively predicted it, opposite to our hypothesis. Collectivistic tendencies (measured by the Collectivism Scale) did not significantly predict the ASR-YWMS (H4.1).

**Table 7:** Results from Bayesian multilevel modeling

	Acceptance of sugar relationships in young women and men scale					Acceptance of sugar relationships in older men and women scale				
	*b*	SE	95% Bayesian CI		BF (H1 vs. H0)	*b*	SE	95% Bayesian CI		BF (H1 vs. H0)
			Lower	Upper				Lower	Upper	
Intercept	− .04	.03	− .10	.02		.02	.03	− .03	.07	
Individual-level predictors										
Sex (Ref: men)	.03	.01	.02	.05	.00	− .06	.01	− .08	− .05	**Infinite**
Age	− .16	.00	− .17	− .15	.00	− .11	.00	− .11	− .10	.00
Sociosexual orientation	.33	.01	.30	.35	**Infinite**	.29	.01	.27	.31	**Infinite**
Traditional gender roles	.08	.01	.06	.10	**Infinite**	.08	.01	.06	.10	**Infinite**
Collectivism scale	.00	.01	− .02	.01	2.65	.01	.01	− .01	.02	2.94
Pathogen prevalence	.03	.01	.02	.04	**3999.00**	.03	.01	.01	.04	**Infinite**
Country-level predictors										
HDI	.07	.05	− .03	.18	.09	.06	.05	− .03	.16	.11
Gender inequality	.06	.05	− .05	.16	**6.45**	.02	.05	− .07	.11	1.77
Ingroup favoritism	− .04	.03	− .10	.02	.12	.04	.03	− .02	.09	**8.59**
Parasite stress	.09	.04	.01	.18	**56.97**	.10	.04	.02	.17	**152.85**

Bolded data are Bayes Factors when they exceeded 3, which represented the presence of evidence positively supporting each hypothesis

At the country level, both gender inequality (measured by the Gender Inequality Index) and parasite stress (measured by the Country-specific zoonotic and non-zoonotic parasite stress) positively predicted the outcome variable. Collectivism (measured by the 178-nation index of Ingroup Favoritism) was negatively associated with the variable which was consistent with our hypothesis. However, the HDI was positively associated with the variable, contrary to our hypothesis (H4.2).

We also found several significant outcomes when the ASR-OMWS was analyzed. At the individual level (H4.3), sociosexual orientation, traditional gender roles, and pathogen prevalence were positively associated with the ASR-OMWS as hypothesized. Consistent with the hypothesis, women were less accepting of sugar relationships from the perspective of older resource providers. In contrast to the hypothesis, older people reported less acceptance of sugar relationships from both perspectives (ASR-OMWS and ASR-YWMS). Collectivism was not significantly associated with either score.

At the country-level (H4.4) parasite stress positively predicted the ASR-OMWS as we hypothesized. However, the HDI and ingroup favoritism were positively associated with the variable contrary to the hypothesis. The gender inequality did not show any significant association.

## Discussion

The present study expands the existing knowledge of relationships involving exchanges of sex for resources in human mating. We contribute a cross-cultural comparative analysis of acceptance of sugar relationships across demographic, psychological, and cultural dimensions. Although many empirical studies of sex for resources have been conducted with geographically and culturally diverse samples, the present study is the first to provide comparative data on ASR scales (designed to explore attitudes from both the perspectives of potential companionship providers and resource providers) for 87 countries. Furthermore, we also examined and validated the ASR scales across different languages through the measurement invariance test and alignment. Researchers who intend to conduct cross-cultural and cross-national studies on the topic may employ the scales with measurement alignment. The study revealed several novel findings, which are discussed below.

### Cross-Linguistic and Cross-Sexual Construct Equivalence, Internal Consistency (H1)

One aim was assessing the psychometric properties of the ASR-YWMS and ASR-OMWS with a large multinational sample. In order to compare the ASR scales with other psychometric instruments on a multilingual sample, it was necessary to test the linguistic invariance of all the scales used. The findings demonstrate that all 37 translated versions of the two measures (except for the Persian version of the ASR-YWMS) show adequate psychometric properties in terms of factor structure, explained variance, internal consistency, and cross-linguistic construct equivalence. All translated versions of the two measures were expected to show cross-linguistic construct equivalence with the respective original measures and adequate psychometric properties in terms of factor structure, explained variance, and internal consistency (H1.1). Our findings suggest that the contents of the two five-item scales (and one exception each of their translated versions) adequately reveal the universal human mating psychology underlying the cultural diversity of ASR, and therefore may provide useful instruments in further cross-cultural comparative analyses. Our results also support the measurement invariance of the other instruments (the SOI-3, the GEMS, and the Individualism–Collectivism scale). These results support Hypothesis 1.1.

As there was no sex variance in the original ASR-YWMS and ASR-OMWS measures, it was necessary to control for sexual invariance in the actual multicultural sample. When testing for sexual invariance, we found that both ASR scales are consistent measures across men and women. These results support Hypothesis 1.2.

### Sex Differences of the Acceptance of Sugar Relationships (H2)

The previously obtained sex differences in scores on ASR-YWMS and ASR-OMWS (men consistently being more accepting than women) were replicated in the present study. Men scored significantly higher on both measures (ASR-YWMS and ASR-OMWS) than women did. According to H2, the previously obtained cross-sex construct equivalences were expected with higher male scores on both ASR measures. Even though these results confirm Hypothesis 2, an interesting contradiction arises here that needs to be clarified. Indeed, in the theoretical introduction it was stated that “women prefer partners who are older and wealthier than themselves,” which implies that women should be more open to sugar relationships from a companionship provider perspective (ASR-YWMS) than men. What could be the reason for this contradiction? The answer is probably multi-causal, as is the way the sex-for-resources encounter works. Our data confirm that participants perceive sugar relations as part of short-term mating (positive correlation with unrestricted sociosexuality). This may explain why men are more open to it, as several studies have found marked gender differences in sexual drive in the same direction (e.g., Buss & Schmitt, 1993, 2019; Frankenbach et al., 2022; Meskó et al., 2022a, 2022b, 2022c; Trivers, 1972; Walter et al., 2020). However, it should be noted that women who engage in sugar relationships also face significant peer stigma (e.g., Ernst et al., 2021; Grittner & Walsh, 2020; Johansson Wilén & Gunnarsson, 2023; Scull, 2022), which is also likely to play a role in the higher rejection of this sexual activity among women compared to men. For a broader understanding of this phenomenon, it is worth considering that openness to sugar relationships is also associated with psychological variables such as egocentric sexual motivation, Ludus (playful) love style, higher subclinical psychopathy, and Machiavellianism scores (Birkás et al., 2020), which are associated with short-term mating strategy. We can probably better understand these sex differences in the acceptance of sugar relationships if we can consider individual differences in mating strategies and contextual influences (Gangestad & Simpson, 2000).

### Intra- and Cross-Subregional Differences and Similarities (H3)

The socioeconomic context in which individuals form their attitudes and make their mating decisions is likely to affect their openness to sugar relationships. Since data from 87 countries were analyzed by 14 geographic subregions, we first confirmed measurement invariance across subregions in the ASR-YWMS and ASR-OMWS. These results support Hypothesis 3.1.

Since cultural factors may influence individuals' mating attitudes in different ways, we hypothesized that the degree of openness to sugar relationships may also vary by subregion. We found significant variation across subregions both in ASR-YWMS and in ASR-OMWS. These results support Hypothesis 3.2.

Given that each participant, irrespective of their actual age, completed both the ASR-YWMS and ASR-OMWS in the present study, the obtained data allowed for the exploration of possible subregional differences in the relative preferences for being a companionship provider (ASR-YWMS) vs. resource provider (ASR-OMWS). The related results consistently revealed significantly higher preference for being a companionship (vs. resource) provider, except in Western Asia where no difference was found between the two perspectives, and in North Africa where higher relative preference for being a resource provider was found. Finding a valid explanation for the results raises several difficulties. For example, the mean age of the Dutch sample was 49.50 years, while that of the Thai sample was 21.13 years (see Table S2 in Supplementary Materials). At the same time, the Dutch participants’ mean scores on the ASR-YWMS and ASR-OMWS, respectively, were 13.95 and 13.40, while the respective scores were 23.90 and 23.88 in the Thai sample. That is, the differences are presumably due to cultural differences between the Thai and Dutch samples rather than to a lack of construct equivalence between the two translated versions of either the ASR-YWMS or the ASR-OMW (Brodeur et al., 2018; Krisch et al., 2019). These results support Hypothesis 3.3.

All these hypotheses (H3.1–3.3) were tested at the country level as shown in the online Supplementary Materials. The results at the country level were in general similar to those already found at the subregional level.

The first main set of questions (H1–3) of the present study dealt with the psychometric analysis of the ASR-YWMS and the ASR-OMWS. This revealed that both measures are characterized by reliability and measurement invariance in linguistic, sexual, and regional terms. The second main set of research questions (H4) was aimed at the associations between openness to sugar relationships and individual and cultural level predictor variables.

### Associations with Individual- and Culture-Level Predictors (H4)

The Bayesian multilevel models revealed the predictive power of, and interactions among, regional, demographic, economic and cultural variables for the ASR-YWMS and the ASR-OMWS. According to H4, both ASR measures were expected to be associated positively with male sex, higher age, unrestricted sociosexuality, and higher parasite history, but negatively associated with individualism, gender equality, and social welfare. Overall, our results partially supported Hypothesis 4: there were some cases where we found no significant associations between the target and predictor variables, and in some cases, we found associations in directions opposite to the expected.

#### Individual-Level Predictors on Acceptance of Sugar Relationships with Younger Women and Men Scale

Openness to sugar relationships as sexual companionship provider (ASR-YWMS) was positively associated with unrestricted sociosexual orientation, identification with traditional gender roles, and prevalence of pathogens at the individual level. Contrary to our hypothesis, we found that being woman positively and age negatively predicted openness to sugar relationships. The collectivism variable (collectivistic personal values) did not significantly predict ASR-YWMS. These results partly support Hypothesis H4.1.

Previous research on ASR (Ipolyi et al., 2021; Láng et al., 2021; Birkás et al., 2020) had a methodological assumption that younger (18–28 years old) male participants could only complete the ASR-YWMS (due to their age). It is possible that the previous research unnecessarily constrained itself with this assumption. Indeed, it is possible that young men who completed the ASR-YWMS assumed that their current or decades older selves would pay for sex (and did not consider which side of this sexual transaction was targeted by the questionnaire offered to them). The method used in the present study (each participant completing both ASR-YWMS and ASR-OMWS) allowed respondents to judge the two aspects independently. That is, they could compare and decide which attitude (paying for sex or sex for payment) was more characteristic of them. Our results showed that women were more accepting of being the younger partner in a sugar relationship (ASR-YWMS), but less accepting of being the older partner (ASR-OMWS), as expected. It is possible that these results reflect the typical age gap in the sugar relationship scenario: men are typically older, and women are typically much younger. This means that women might have a more positive attitude toward the role of “sugar baby” than men toward the role of “sugar boy”. At the same time, women might have more negative attitudes toward the role of “sugar mommy” than men toward the role of “sugar daddy”. Overall, although these results only partially support the hypotheses formulated based on previous research, they are in fact well interpretable in terms of the psychological functioning of sugar relationships. This expectation is based on the view that engaging in sex for resources is a general preference that is an essential part of the psychology of human mating. Women are more likely to prefer men who are older and wealthier than themselves, whereas men are more likely to prefer younger women (Walter et al., 2020, 2021).

#### Country-Level Predictors on Acceptance of Sugar Relationships with Younger Women and Men Scale

Openness to sugar relationships as sexual companionship provider (ASR-YWMS) was positively associated with both gender inequality and parasite stress at the country-level. Collectivism (collectivistic value by countries) was negatively associated with openness to sugar relationships, which is in line with our hypothesis. Contrary to our hypothesis, we found that HDI was positively associated with openness to sugar relationships. These results partly support Hypothesis H4.2.

The result on HDI shows that material well-being (higher HDI), rather than being negatively related to young people's openness to sugar relationships, was in fact positively related to it. This unexpected association raises some considerations. Although in the US-American and Danish studies (Groes et al., 2021; Scull, 2022) the most important motivation reported by participants in sugar relationships was money, our findings suggest that material resources may not be the most important motivation for being open to sugar relationships. Nevertheless, it is possible that material wealth may predict the extraction of material goods in countries with high HDI. Our results suggest that economic rationale may be significantly associated with individual decisions to buy and sell sex as a commodity (Mensah et al., 2022; Van der Veen, 2001). Whether individuals are driven by extrinsic or intrinsic motivations may also play a role. Ipolyi et al. (2021) found that scores on both the ASR-YWMS and ASR-OMWS were positively associated with extrinsic motivation focused on external values (e.g., financial success, social status, reputation, attractive appearance).

#### Individual-Level Predictors on Acceptance of Sugar Relationships with Older Men and Women Scale

Openness to sugar relationships as a resource provider (ASR-OMWS) was positively associated with unrestricted sociosexual orientation, identification with traditional gender roles, and prevalence of pathogens at the individual level. Consistent with our hypothesis, we found that being men positively predicted openness to sugar relationships. Collectivism (collectivistic personal values) did not significantly predict ASR-OMWS, not confirming our hypothesis. Contrary to our hypothesis, age negatively predicted openness to sugar relationships. These results partly support Hypothesis H4.3.

Collectivistic personal values showed no correlation with either ASR-YWMS or ASR-OMWS scores. A possible explanation is methodological. In this research, we used a collectivism scale (Wu, 2006) that can be measured primarily in the context of employment. Individualistic/collectivistic values in the work context are unlikely to be transposed one-to-one to the mating context. Future studies will be needed to test this explanation.

#### Country-Level Predictors on Acceptance of Sugar Relationships with Older Men and Women Scale

Openness to sugar relationships as a resource provider (ASR-OMWS) was positively associated with parasite stress as hypothesized. However, HDI and ingroup favoritism was positively associated with the openness to sugar relationships, contrary to the hypothesis. Gender inequality index did not show any significant association. These results partly support Hypothesis H4.4.

The most striking positive association is between pathogen saturation and openness to sugar relationships. Both individual-level and country-level measures support the hypotheses on both ASR scales. These results support previous findings. Since sexual contact is an important route of human-to-human transmission of pathogens, promiscuous sexual behavior plays an explicit role in the spread of infections (Richard et al., 2017). The greater the prevalence of pathogens within a community, the greater the threat to that community's viability (Mackey & Immerman, 2000).

Cross-culturally, pathogen stress levels correlate within a culture and the prevalence of polygynous marriage systems, and monogamous mating systems are less common in cultures with high pathogen stress (Low, 1990; White & Burton, 1988). Research by Marlowe (2003) has also suggested that monogamy is more prevalent in forager cultures with low pathogen levels and where men contribute a significant proportion of calories to the local diet.

The most contradictory results come from the analysis between collectivism and ASR. While the personal collectivism value (collectivism scale) showed no association with any of the ASR scales, country-level collectivism (ingroup favoritism) showed a negative association only with ASR-YWMS (as expected), but a positive association with ASR-OMWS. So, while collectivism is negatively correlated with young people's openness to participate in sex-for-resources, for older people it is positively correlated. This may relate to the differing typical age range for men and women in sugar relationships: the restrictive effect of collectivist values on female sex-for-resources behavior is well documented. For example, Li et al. (2022) found that female Chinese sex workers who faced stigma because of their activities were able to reduce the distress caused by stigma by attenuating their personal networks, which emphasized collectivist values. However, there is a paucity of literature on the collectivist norms associated with the purchase of sex.

An alternative explanation is that women’s openness to engage in sex for resources in patriarchal societies is part of the “staying alive” strategy (Campbell, 1999). Since women’s overt aggression in the competition for limited resources is usually disapproved (Benenson et al., 2022; Bleske-Rechek & Deaner, 2022; Campbell, 1999), they may employ alternative strategies. That is, survival motives may also have contributed to the emergence of female promiscuity (gaining men’s resources by adapting to male promiscuity).

### Limitations and Future Directions

Although we addressed possible explanations for the observed associations between openness to sex for resources and the tested predictors, due to the exploratory nature of the study, the findings cannot offer a full picture of the mechanisms via which the studied factors exert influence on one’s attitude toward sugar relationships.

Moreover, because the sampling was convenience-based, disproportionalities sometimes emerged (e.g., the Dutch sample was on average twice as old as the Thai sample). These suggest that our results have limited validity when comparing national samples. Further studies will be needed to paint a more accurate picture.

One of the main questions of this research is whether openness to sugar relationships is related to promiscuity. The results show that both ASR scales are positively related to the SOI-3, thus replicating previous similar findings (Birkás et al., 2020; Láng et al., 2021). However, it should be noted that other research (e.g., Chu, 2023; Motyl, 2012) also reports that some sugar relationships are long-term relationships. In this respect, our results are of limited, as we found no evidence for a long-term strategy.

One anonymous reviewer of the manuscript suggested that when we decided to use a culturally specific term (sugar relationship) in the research rather than the broader concept of "sex for resources" we may have made it difficult for some participants to understand the questionnaire. Although the results of the statistical analysis do not support this concern, we should consider the possibility that data are drawn from multiple countries, some of them might have already incorporated the idea of resource exchange into their mainstream understandings of relationships.

In this research, we did not take into account cultural differences in the acceptability of the explicitness of transactions described in ASR scales. Many intimate arrangements cross-culturally and throughout history were also about an exchange of resources, irrespective of the form of relationship. Our research does not focus on the question of how these other arrangements relate to the sugar relationships or what distinctions can be drawn between them. At the same time, there can be significant differences between cultures depending on the extent to which they have incorporated the "sex for resources" aspect into their mating rituals. Some cultures have long preferred marriages/ relationships that occur between individuals of similar social class or socioeconomic status; others have allowed for more mobility. Further, some cultures allow for an explicit consideration and discussion of the resources exchanged in a match (economic/financial/status in exchange for companionship/reproductive capacity, etc.) while others prefer not to speak of such considerations directly. However, these culturally embedded norms can also have an impact on attitudes measured on the ASR scales. These possible effects need to be clarified in future research.

### Conclusions

The current research represents the largest investigation of predictors of openness to sugar relationships, testing the psychometric properties of ASR-YWMS and ASR-OMWS, based on data from 69,924 participants across 87 countries. As such, it takes an important step toward understanding the opening attitude toward participating in sex for resources, across cultures or demographic groups, alongside factors operating at the individual level. The main strength of this research is its cross-cultural nature and large sample size, which allows a systematic examining of the impact of individual and cultural variables and the factors that explain the greatest variation in openness to sugar relationships. We believe that a more nuanced understanding of the phenomenon of openness to sugar relationships will provide fundamental insights into the psychology of human mating and may translate into developing more effective ways to counteract the possible negative psychological consequences of participating in sex-for-resources type of encounters.

### Supplementary Information

Below is the link to the electronic supplementary material.

Supplementary file1 (DOCX 255 kb)

## Data Availability

All data and Supplementary Materials have been made publicly available at the Open Science Framework (OSF) and can be accessed at https://osf.io/prdj2/?view_only=48e348faeac344a59554ad44c7e89482.

## Footnotes

1 Following an anonymous reviewer’s suggestion, we also conducted tests for H3s at the country level. First, we were able to achieve scalar invariance required for cross-country comparisons via measurement alignment (relevant to H3.1). Second, both the ASR-YWMS and ASR-OMWS reported significant cross-country differences as shown at the subregional level (relevant to H3.2). Third, we also examined the difference between the ASR-YWMS and ASR-OMWS in each country (relevant to H3.3). See “Additional Country-wise Analyses” section in our online supplementary material at https://osf.io/4umga?view_only=48e348faeac344a59554ad44c7e89482 or our OSF repository for further details.

2 One anonymous reviewer raised a concern about the possibility that the associations between several predictors at the individual level (e.g., collectivism) and dependent variables not supporting the hypothesis may be attributable to the shared variance across the individual and country levels. To examine whether that was the case, we tested the regression models without random slopes. The additional analyses reported that for both the ASR-YWMS and ASR-OMWS as dependent variables, the exclusion of the random slopes did not alter the analysis results regarding the individual-level predictors. Plus, the exclusion of ingroup favoritism at the country level also did not alter the results as well. See “Additional Analyses related to Multilevel Modeling” section in our online supplementary material at https://osf.io/4umga?view_only=48e348faeac344a59554ad44c7e89482 or our OSF repository for further details). Hence, we shall conclude that the potential shared variance across the different levels would not be a serious concern while interpreting the findings in Table 7.

3 Given that the SOI-3 consists of only three items and it demonstrated strong correlation (*r* ≥ .50) with the variables of interest, we examined its incremental validity in the models via additional MLMs following one reviewer’s suggestion. The results indicated that the inclusion of the SOI-3 significantly improved the regression models, so its incremental validity was also significant. See “Incremental Validity of the Sociosexual Orientation” section in our online supplementary material at https://osf.io/4umga?view_only=48e348faeac344a59554ad44c7e89482 or our OSF repository for further details.
