# Supplementary material for: Exploring Attitudes Toward “Sugar Relationships” Across 87 Countries: A Global Perspective on Exchanges of Resources for Sex and Companionship
Source: Arch Sex Behav. 2023 Dec 21;53(2):811–37. doi: 10.1007/s10508-023-02724-1 (PMC10844470; doi:10.1007/s10508-023-02724-1)
Supplement: Supplementary file 1 — Supplementary file1 (DOCX 255 kb) [file 10508_2023_2724_MOESM1_ESM.docx]

**Exploring Attitudes Towards “Sugar Relationships” Across 87 Countries: A Global Perspective on Exchanges of Resources for Sex and Companionship**

**Supplementary Materials**

[Instruction for the translators when translating the survey into local languages 3](#_Toc126406967)

[Data collection 4](#_Toc126406968)

[Table S1.A. Detailed participants’ descriptive characteristics across countries (age, gender, sex at birth). 6](#_Toc126406969)

[Table S1.B. Detailed participants’ descriptive characteristics across countries (relationship status, employment). 10](#_Toc126406970)

[Table S2. Descriptive statistics along languages. 14](#_Toc126406971)

[Table S3. Reliability coefficients (McDonald's ω) of scales used in the study along languages. 15](#_Toc126406972)

[Language: Arab 16](#_Toc126406973)

[Language: Bosnian 17](#_Toc126406974)

[Language: Brazilian Portuguese 18](#_Toc126406975)

[Language: Bulgarian 19](#_Toc126406976)

[Language: Chinese (traditional) 20](#_Toc126406977)

[Language: Croatian 21](#_Toc126406978)

[Language: Czech 22](#_Toc126406979)

[Language: Dutch 23](#_Toc126406980)

[Language: English 24](#_Toc126406981)

[Language: Estonian 25](#_Toc126406982)

[Language: Finnish 26](#_Toc126406983)

[Language: French 27](#_Toc126406984)

[Language: Georgian 28](#_Toc126406985)

[Language: German 29](#_Toc126406986)

[Language: Greek 30](#_Toc126406987)

[Language: Hebrew 31](#_Toc126406988)

[Language: Hungarian 32](#_Toc126406989)

[Language: Italian 33](#_Toc126406990)

[Language: Japanese 34](#_Toc126406991)

[Language: Korean 35](#_Toc126406992)

[Language: Lithuanian 36](#_Toc126406993)

[Language: Macedonian 37](#_Toc126406994)

[Language: Malay 38](#_Toc126406995)

[Language: Norwegian 39](#_Toc126406996)

[Language: Persian 40](#_Toc126406997)

[Language: Polish 41](#_Toc126406998)

[Language: Portuguese 42](#_Toc126406999)

[Language: Romanian 43](#_Toc126407000)

[Language: Russian 44](#_Toc126407001)

[Language: Serbian 45](#_Toc126407002)

[Language: Slovak 46](#_Toc126407003)

[Language: Slovenian 47](#_Toc126407004)

[Language: Spanish 48](#_Toc126407005)

[Language: Spanish (Latin American) 49](#_Toc126407006)

[Language: Swedish 50](#_Toc126407007)

[Language: Turkish 51](#_Toc126407008)

[Language: Ukrainian 52](#_Toc126407009)

Instruction for the translators when translating the survey into local languages

How should the translation look?

We follow the guidelines of WHO (https://www.who.int/substance_abuse/research_tools/translation/en/). In brief:

1. At least one (ideally more than one) person will do a forward translation (from English to your native language). If more than one person is involved in this part, they work together and discuss all issues. Once this is done, it is time for:

2. At least one (ideally more than one) person will now take the version of the questionnaire translated by the other team and translate this version back to English from your native language. If more than one person is involved in this part, they work together and discuss all issues. Once this is done, it is time for:

3. At the end, all translators compare two versions of the questionnaire and spot any differences. Please, at this point, try to figure out why such differences appeared. Do they substantially change the meaning? Maybe some questions would need slight alterations? Our goal is to investigate psychological phenomena. Thus, the equivalence of our measures is super important. If we do not achieve the equivalence of invariance, we would not draw any conclusions from our study, as any potential differences between our participants from different countries would be attributed to the differences in measures and not underlying across individuals or countries factors.

4. Please, copy the final version into the sheet called “FINAL” and let me know. I will then upload your translation into Qualtrics and send you the link to the study. I will then ask you to make sure that everything is ok and nothing got lost or twisted in the process.

5. IMPORTANT THINGS:

During the translation, one of the most essential things would be to translate the questions/items but WITHOUT changing anything else. I know that the excel file looks a bit messy, but that is because it has some HTML formatting inserted (so to make our survey look more visually appealing). If you, by accident, delete some “<” signs, it will result in errors in the layout of the study. So please, translate ONLY English words and ignore the rest, leaving it unaltered (as shown in the excel file, sheet “INSTRUCTIONS”).

If the local language in your country is SIMILAR (or identical) to languages of other countries (e.g., Spanish, Portuguese), please, let me know. I can then assign you to the given language translation group (so you would join forces).

All the best,
Marta Kowal

Data collection

Data were collected mostly online in all but two countries*,* as participants from Algeria and Morocco could not access the Qualtrics website. Thus, collaborators collected data in person using a paper-pencil method. Furthermore, Iranian participants also had difficulties accessing the Qualtrics website, so we re-created the survey and collected data using Google Forms. One Russian Collaborator collected data using the Toloka website (a crowdsourcing platform popular in Russia)*.*

#

Table S1.A. Detailed participants’ descriptive characteristics across countries (age, gender, sex at birth).

| Country | N | Age | | Gender | | | | | | | | Sex at birth | | | | | |
| --- | --- | --- | --- | --- | --- | --- | --- | --- | --- | --- | --- | --- | --- | --- | --- | --- | --- |
|  |  | Mean | SD | Men (%) | | Women (%) | | Non-binary /  third gender (%) | | Prefer not  to say (%) | | Male (%) | | Female (%) | | Intersex (%) | |
| Algeria | 1 530 | 28,10 | 9,55 | 500 | (32,68%) | 1 026 | (67,06%) | 1 | (,07%) | 3 | (,20%) | 497 | (32,48%) | 1 031 | (67,39%) | 2 | (,13%) |
| Angola | 47 | 33,72 | 7,89 | 26 | (55,32%) | 21 | (44,68%) | 0 | (,00%) | 0 | (,00%) | 26 | (55,32%) | 21 | (44,68%) | 0 | (,00%) |
| Argentina | 877 | 40,24 | 16,34 | 391 | (44,58%) | 476 | (54,28%) | 3 | (,34%) | 7 | (,80%) | 396 | (45,15%) | 478 | (54,50%) | 3 | (,34%) |
| Australia | 387 | 31,74 | 11,55 | 181 | (46,77%) | 198 | (51,16%) | 6 | (1,55%) | 2 | (,52%) | 181 | (46,89%) | 203 | (52,59%) | 2 | (,52%) |
| Austria | 154 | 28,75 | 10,00 | 44 | (28,57%) | 104 | (67,53%) | 5 | (3,25%) | 1 | (,65%) | 44 | (28,76%) | 109 | (71,24%) | 0 | (,00%) |
| Azerbaijan | 35 | 21,63 | 5,25 | 24 | (68,57%) | 9 | (25,71%) | 2 | (5,71%) | 0 | (,00%) | 24 | (68,57%) | 10 | (28,57%) | 1 | (2,86%) |
| Bahrain | 36 | 28,08 | 7,51 | 21 | (58,33%) | 15 | (41,67%) | 0 | (,00%) | 0 | (,00%) | 20 | (55,56%) | 15 | (41,67%) | 1 | (2,78%) |
| Bangladesh | 30 | 23,00 | 5,54 | 23 | (76,67%) | 6 | (20,00%) | 0 | (,00%) | 1 | (3,33%) | 23 | (76,67%) | 7 | (23,33%) | 0 | (,00%) |
| Belarus | 100 | 25,39 | 9,89 | 30 | (30,00%) | 63 | (63,00%) | 3 | (3,00%) | 4 | (4,00%) | 31 | (31,00%) | 68 | (68,00%) | 1 | (1,00%) |
| Belgium | 1 806 | 44,23 | 17,37 | 782 | (43,30%) | 1 021 | (56,53%) | 2 | (,11%) | 1 | (,06%) | 779 | (43,13%) | 1 024 | (56,70%) | 3 | (,17%) |
| Bolivia | 38 | 25,13 | 6,85 | 10 | (26,32%) | 28 | (73,68%) | 0 | (,00%) | 0 | (,00%) | 10 | (27,03%) | 27 | (72,97%) | 0 | (,00%) |
| Bosnia and Herzegovina | 293 | 26,69 | 9,64 | 59 | (20,14%) | 231 | (78,84%) | 0 | (,00%) | 3 | (1,02%) | 62 | (21,16%) | 231 | (78,84%) | 0 | (,00%) |
| Brazil | 1 104 | 29,67 | 9,49 | 459 | (41,58%) | 630 | (57,07%) | 10 | (,91%) | 5 | (,45%) | 463 | (41,94%) | 638 | (57,79%) | 3 | (,27%) |
| Brunei | 163 | 26,41 | 6,44 | 74 | (45,40%) | 88 | (53,99%) | 1 | (,61%) | 0 | (,00%) | 75 | (46,01%) | 88 | (53,99%) | 0 | (,00%) |
| Bulgaria | 229 | 24,65 | 6,17 | 47 | (20,52%) | 180 | (78,60%) | 2 | (,87%) | 0 | (,00%) | 45 | (19,65%) | 184 | (80,35%) | 0 | (,00%) |
| Canada | 750 | 32,32 | 10,69 | 351 | (46,80%) | 380 | (50,67%) | 18 | (2,40%) | 1 | (,13%) | 356 | (47,59%) | 392 | (52,41%) | 0 | (,00%) |
| Chile | 1 204 | 29,53 | 7,41 | 258 | (21,43%) | 905 | (75,17%) | 28 | (2,33%) | 13 | (1,08%) | 261 | (21,68%) | 942 | (78,24%) | 1 | (,08%) |
| China | 46 | 26,39 | 9,17 | 23 | (50,00%) | 20 | (43,48%) | 2 | (4,35%) | 1 | (2,17%) | 23 | (50,00%) | 21 | (45,65%) | 2 | (4,35%) |
| Colombia | 535 | 26,78 | 11,65 | 183 | (34,21%) | 348 | (65,05%) | 3 | (,56%) | 1 | (,19%) | 184 | (34,39%) | 350 | (65,42%) | 1 | (,19%) |
| Croatia | 1 637 | 27,34 | 11,21 | 385 | (23,52%) | 1 231 | (75,20%) | 14 | (,86%) | 7 | (,43%) | 389 | (23,76%) | 1 247 | (76,18%) | 1 | (,06%) |
| Cyprus | 164 | 29,54 | 10,29 | 71 | (43,29%) | 93 | (56,71%) | 0 | (,00%) | 0 | (,00%) | 70 | (42,68%) | 93 | (56,71%) | 1 | (,61%) |
| Czech Republic | 846 | 26,85 | 8,77 | 228 | (26,95%) | 612 | (72,34%) | 4 | (,47%) | 2 | (,24%) | 227 | (26,86%) | 616 | (72,90%) | 2 | (,24%) |
| Denmark | 290 | 27,11 | 8,06 | 72 | (24,83%) | 215 | (74,14%) | 3 | (1,03%) | 0 | (,00%) | 70 | (24,22%) | 218 | (75,43%) | 1 | (,35%) |
| Dominican Republic | 354 | 25,18 | 10,32 | 109 | (30,79%) | 244 | (68,93%) | 1 | (,28%) | 0 | (,00%) | 108 | (30,51%) | 246 | (69,49%) | 0 | (,00%) |
| Ecuador | 1 384 | 30,08 | 11,55 | 604 | (43,64%) | 776 | (56,07%) | 2 | (,14%) | 2 | (,14%) | 606 | (43,79%) | 778 | (56,21%) | 0 | (,00%) |
| Egypt | 37 | 25,30 | 5,05 | 22 | (59,46%) | 15 | (40,54%) | 0 | (,00%) | 0 | (,00%) | 22 | (59,46%) | 15 | (40,54%) | 0 | (,00%) |
| El Salvador | 208 | 25,09 | 9,84 | 89 | (42,79%) | 115 | (55,29%) | 4 | (1,92%) | 0 | (,00%) | 90 | (43,27%) | 116 | (55,77%) | 2 | (,96%) |
| Estonia | 365 | 33,38 | 12,09 | 59 | (16,16%) | 302 | (82,74%) | 1 | (,27%) | 3 | (,82%) | 62 | (16,99%) | 303 | (83,01%) | 0 | (,00%) |
| Finland | 588 | 32,02 | 10,59 | 108 | (18,37%) | 448 | (76,19%) | 28 | (4,76%) | 4 | (,68%) | 110 | (18,71%) | 478 | (81,29%) | 0 | (,00%) |
| France | 1 590 | 27,23 | 10,50 | 406 | (25,53%) | 1 139 | (71,64%) | 40 | (2,52%) | 5 | (,31%) | 406 | (25,55%) | 1 181 | (74,32%) | 2 | (,13%) |
| Georgia | 469 | 24,67 | 10,75 | 96 | (20,56%) | 365 | (78,16%) | 2 | (,43%) | 4 | (,86%) | 98 | (20,90%) | 370 | (78,89%) | 1 | (,21%) |
| Germany | 562 | 28,84 | 10,08 | 177 | (31,49%) | 367 | (65,30%) | 13 | (2,31%) | 5 | (,89%) | 180 | (32,09%) | 379 | (67,56%) | 2 | (,36%) |
| Ghana | 289 | 33,89 | 13,83 | 270 | (93,75%) | 15 | (5,21%) | 1 | (,35%) | 2 | (,69%) | 273 | (94,46%) | 16 | (5,54%) | 0 | (,00%) |
| Greece | 825 | 28,43 | 10,85 | 128 | (15,52%) | 687 | (83,27%) | 7 | (,85%) | 3 | (,36%) | 133 | (16,12%) | 691 | (83,76%) | 1 | (,12%) |
| Guatemala | 40 | 36,90 | 10,56 | 16 | (40,00%) | 23 | (57,50%) | 1 | (2,50%) | 0 | (,00%) | 16 | (40,00%) | 23 | (57,50%) | 1 | (2,50%) |
| Honduras | 570 | 25,79 | 8,46 | 188 | (32,98%) | 376 | (65,96%) | 2 | (,35%) | 4 | (,70%) | 189 | (33,16%) | 380 | (66,67%) | 1 | (,18%) |
| Hungary | 530 | 25,92 | 9,21 | 109 | (20,57%) | 410 | (77,36%) | 9 | (1,70%) | 2 | (,38%) | 110 | (20,75%) | 420 | (79,25%) | 0 | (,00%) |
| India | 364 | 28,46 | 8,95 | 218 | (59,89%) | 144 | (39,56%) | 1 | (,27%) | 1 | (,27%) | 218 | (60,06%) | 145 | (39,94%) | 0 | (,00%) |
| Iran | 571 | 30,37 | 9,09 | 179 | (31,35%) | 386 | (67,60%) | 0 | (,00%) | 6 | (1,05%) | 1 | (50,00%) | 1 | (50,00%) | 0 | (,00%) |
| Ireland | 348 | 35,58 | 9,76 | 89 | (25,57%) | 254 | (72,99%) | 4 | (1,15%) | 1 | (,29%) | 91 | (26,15%) | 257 | (73,85%) | 0 | (,00%) |
| Israel | 1 012 | 33,32 | 13,33 | 384 | (37,94%) | 624 | (61,66%) | 2 | (,20%) | 2 | (,20%) | 386 | (38,14%) | 625 | (61,76%) | 1 | (,10%) |
| Italy | 3 005 | 31,00 | 12,16 | 882 | (29,35%) | 2 102 | (69,95%) | 17 | (,57%) | 4 | (,13%) | 881 | (29,34%) | 2 120 | (70,60%) | 2 | (,07%) |
| Jamaica | 41 | 34,37 | 9,44 | 9 | (21,95%) | 32 | (78,05%) | 0 | (,00%) | 0 | (,00%) | 9 | (21,95%) | 32 | (78,05%) | 0 | (,00%) |
| Japan | 1 910 | 40,91 | 11,75 | 1 002 | (52,46%) | 875 | (45,81%) | 16 | (,84%) | 17 | (,89%) | 1 012 | (52,98%) | 893 | (46,75%) | 5 | (,26%) |
| Jordan | 33 | 26,12 | 7,77 | 21 | (63,64%) | 11 | (33,33%) | 1 | (3,03%) | 0 | (,00%) | 21 | (63,64%) | 12 | (36,36%) | 0 | (,00%) |
| Kazakhstan | 491 | 28,91 | 11,25 | 148 | (30,14%) | 341 | (69,45%) | 2 | (,41%) | 0 | (,00%) | 148 | (30,14%) | 343 | (69,86%) | 0 | (,00%) |
| Kenya | 316 | 25,13 | 3,89 | 186 | (58,86%) | 128 | (40,51%) | 1 | (,32%) | 1 | (,32%) | 187 | (59,18%) | 128 | (40,51%) | 1 | (,32%) |
| Kuwait | 51 | 26,47 | 6,31 | 40 | (78,43%) | 11 | (21,57%) | 0 | (,00%) | 0 | (,00%) | 40 | (78,43%) | 11 | (21,57%) | 0 | (,00%) |
| Lebanon | 116 | 27,12 | 7,56 | 41 | (35,34%) | 72 | (62,07%) | 3 | (2,59%) | 0 | (,00%) | 41 | (35,34%) | 75 | (64,66%) | 0 | (,00%) |
| Lithuania | 657 | 26,88 | 10,55 | 184 | (28,01%) | 466 | (70,93%) | 5 | (,76%) | 2 | (,30%) | 182 | (27,70%) | 471 | (71,69%) | 4 | (,61%) |
| Macedonia | 1 062 | 27,69 | 10,53 | 394 | (37,10%) | 660 | (62,15%) | 4 | (,38%) | 4 | (,38%) | 394 | (37,10%) | 666 | (62,71%) | 2 | (,19%) |
| Malaysia | 985 | 24,43 | 8,26 | 218 | (22,13%) | 758 | (76,95%) | 3 | (,30%) | 6 | (,61%) | 227 | (23,05%) | 756 | (76,75%) | 2 | (,20%) |
| Mexico | 339 | 28,10 | 8,42 | 171 | (50,44%) | 158 | (46,61%) | 9 | (2,65%) | 1 | (,29%) | 174 | (51,33%) | 164 | (48,38%) | 1 | (,29%) |
| Moldova | 34 | 24,26 | 7,83 | 8 | (23,53%) | 25 | (73,53%) | 0 | (,00%) | 1 | (2,94%) | 9 | (26,47%) | 25 | (73,53%) | 0 | (,00%) |
| Montenegro | 37 | 26,32 | 10,54 | 8 | (21,62%) | 28 | (75,68%) | 0 | (,00%) | 1 | (2,70%) | 8 | (21,62%) | 29 | (78,38%) | 0 | (,00%) |
| Morocco | 2 128 | 29,55 | 6,07 | 1 052 | (49,44%) | 1 076 | (50,56%) | 0 | (,00%) | 0 | (,00%) | 1 050 | (49,34%) | 1 078 | (50,66%) | 0 | (,00%) |
| NA | 172 | 29,82 | 12,87 | 39 | (27,66%) | 98 | (69,50%) | 1 | (,71%) | 3 | (2,13%) | 39 | (28,06%) | 99 | (71,22%) | 1 | (,72%) |
| Netherlands | 488 | 44,57 | 17,91 | 218 | (44,67%) | 265 | (54,30%) | 4 | (,82%) | 1 | (,20%) | 221 | (45,38%) | 265 | (54,41%) | 1 | (,21%) |
| New Zealand | 309 | 45,13 | 17,19 | 148 | (47,90%) | 158 | (51,13%) | 3 | (,97%) | 0 | (,00%) | 149 | (48,22%) | 160 | (51,78%) | 0 | (,00%) |
| Nigeria | 627 | 21,93 | 4,56 | 326 | (51,99%) | 299 | (47,69%) | 0 | (,00%) | 2 | (,32%) | 318 | (51,46%) | 299 | (48,38%) | 1 | (,16%) |
| Norway | 1 312 | 35,68 | 12,36 | 247 | (18,83%) | 1 064 | (81,10%) | 1 | (,08%) | 0 | (,00%) | 246 | (18,79%) | 1 062 | (81,13%) | 1 | (,08%) |
| Pakistan | 225 | 25,32 | 6,19 | 68 | (30,22%) | 155 | (68,89%) | 0 | (,00%) | 2 | (,89%) | 69 | (30,94%) | 154 | (69,06%) | 0 | (,00%) |
| Peru | 48 | 25,69 | 7,58 | 20 | (41,67%) | 27 | (56,25%) | 1 | (2,08%) | 0 | (,00%) | 20 | (41,67%) | 28 | (58,33%) | 0 | (,00%) |
| Philippines | 1 763 | 24,79 | 9,00 | 491 | (27,85%) | 1 155 | (65,51%) | 85 | (4,82%) | 32 | (1,82%) | 535 | (30,36%) | 1 226 | (69,58%) | 1 | (,06%) |
| Poland | 5 535 | 25,94 | 9,20 | 1 401 | (25,32%) | 4 063 | (73,42%) | 55 | (,99%) | 15 | (,27%) | 1 418 | (25,63%) | 4 110 | (74,30%) | 4 | (,07%) |
| Portugal | 1 395 | 30,49 | 11,29 | 500 | (35,84%) | 872 | (62,51%) | 20 | (1,43%) | 3 | (,22%) | 504 | (36,13%) | 891 | (63,87%) | 0 | (,00%) |
| Romania | 788 | 25,45 | 9,66 | 304 | (38,58%) | 482 | (61,17%) | 1 | (,13%) | 1 | (,13%) | 304 | (38,58%) | 484 | (61,42%) | 0 | (,00%) |
| Russia | 2 856 | 26,29 | 10,06 | 860 | (30,13%) | 1 973 | (69,13%) | 14 | (,49%) | 7 | (,25%) | 859 | (30,16%) | 1 988 | (69,80%) | 1 | (,04%) |
| Serbia | 1 297 | 26,84 | 9,90 | 363 | (27,99%) | 929 | (71,63%) | 2 | (,15%) | 3 | (,23%) | 367 | (28,30%) | 930 | (71,70%) | 0 | (,00%) |
| Slovakia | 935 | 23,98 | 6,74 | 220 | (23,53%) | 707 | (75,61%) | 7 | (,75%) | 1 | (,11%) | 221 | (23,64%) | 713 | (76,26%) | 1 | (,11%) |
| Slovenia | 682 | 35,57 | 12,87 | 141 | (20,67%) | 533 | (78,15%) | 6 | (,88%) | 2 | (,29%) | 140 | (20,53%) | 540 | (79,18%) | 2 | (,29%) |
| South Africa | 109 | 27,72 | 7,96 | 56 | (51,38%) | 51 | (46,79%) | 2 | (1,83%) | 0 | (,00%) | 56 | (51,38%) | 53 | (48,62%) | 0 | (,00%) |
| South Korea | 835 | 42,51 | 13,25 | 382 | (45,75%) | 451 | (54,01%) | 1 | (,12%) | 1 | (,12%) | 382 | (45,75%) | 452 | (54,13%) | 1 | (,12%) |
| Spain | 205 | 31,95 | 10,50 | 84 | (40,98%) | 115 | (56,10%) | 5 | (2,44%) | 1 | (,49%) | 88 | (42,93%) | 116 | (56,59%) | 1 | (,49%) |
| Sweden | 341 | 32,38 | 11,63 | 86 | (25,22%) | 245 | (71,85%) | 10 | (2,93%) | 0 | (,00%) | 89 | (26,10%) | 252 | (73,90%) | 0 | (,00%) |
| Switzerland | 420 | 35,58 | 15,98 | 122 | (29,05%) | 294 | (70,00%) | 4 | (,95%) | 0 | (,00%) | 120 | (28,57%) | 300 | (71,43%) | 0 | (,00%) |
| Taiwan | 409 | 31,68 | 8,66 | 195 | (47,68%) | 203 | (49,63%) | 9 | (2,20%) | 2 | (,49%) | 205 | (50,12%) | 203 | (49,63%) | 1 | (,24%) |
| Thailand | 248 | 21,25 | 4,08 | 139 | (56,05%) | 98 | (39,52%) | 9 | (3,63%) | 2 | (,81%) | 138 | (55,65%) | 109 | (43,95%) | 1 | (,40%) |
| Tunisia | 628 | 27,20 | 6,35 | 387 | (61,62%) | 233 | (37,10%) | 3 | (,48%) | 5 | (,80%) | 388 | (61,88%) | 234 | (37,32%) | 5 | (,80%) |
| Türkiye | 7 291 | 26,43 | 10,15 | 2 226 | (30,53%) | 4 996 | (68,52%) | 46 | (,63%) | 23 | (,32%) | 2 238 | (30,70%) | 5 037 | (69,10%) | 14 | (,19%) |
| Uganda | 331 | 27,51 | 8,72 | 184 | (55,59%) | 146 | (44,11%) | 0 | (,00%) | 1 | (,30%) | 183 | (55,29%) | 148 | (44,71%) | 0 | (,00%) |
| UK | 671 | 28,17 | 10,51 | 171 | (25,48%) | 474 | (70,64%) | 20 | (2,98%) | 6 | (,89%) | 174 | (25,93%) | 497 | (74,07%) | 0 | (,00%) |
| Ukraine | 1 422 | 23,62 | 8,33 | 292 | (20,53%) | 1 116 | (78,48%) | 9 | (,63%) | 5 | (,35%) | 297 | (20,90%) | 1 124 | (79,10%) | 0 | (,00%) |
| United Arab Emirates | 40 | 28,50 | 8,87 | 18 | (45,00%) | 21 | (52,50%) | 1 | (2,50%) | 0 | (,00%) | 18 | (45,00%) | 21 | (52,50%) | 1 | (2,50%) |
| Uruguay | 482 | 29,80 | 9,47 | 90 | (18,67%) | 386 | (80,08%) | 4 | (,83%) | 2 | (,41%) | 92 | (19,09%) | 390 | (80,91%) | 0 | (,00%) |
| USA | 3 713 | 36,12 | 13,95 | 1 499 | (40,37%) | 2 155 | (58,04%) | 51 | (1,37%) | 8 | (,22%) | 1 501 | (40,46%) | 2 204 | (59,41%) | 5 | (,13%) |
| Uzbekistan | 182 | 27,25 | 10,33 | 43 | (23,63%) | 139 | (76,37%) | 0 | (,00%) | 0 | (,00%) | 44 | (24,31%) | 137 | (75,69%) | 0 | (,00%) |
| Venezuela | 553 | 28,19 | 11,36 | 272 | (49,19%) | 273 | (49,37%) | 5 | (,90%) | 3 | (,54%) | 273 | (49,37%) | 279 | (50,45%) | 1 | (,18%) |
| Total | 69 924 | 29,68 | 11,98 | 23 449 | (33,55%) | 45 509 | (65,12%) | 665 | (,95%) | 264 | (,38%) | 23 434 | (33,83%) | 45 745 | (66,03%) | 96 | (,14%) |

Table S1.B. Detailed participants’ descriptive characteristics across countries (relationship status, employment).

| Country | N | Relationship status | | | | | | | | Employment status | | | | | | | | | | | |
| --- | --- | --- | --- | --- | --- | --- | --- | --- | --- | --- | --- | --- | --- | --- | --- | --- | --- | --- | --- | --- | --- |
|  |  | Single (%) | | Dating (%) | | In a committed  relationship (%) | | Married (%) | | Student (%) | | Full time employed (%) | | Part time employed (%) | | Self employed (%) | | Unemployed (%) | | Retired (%) | |
| Algeria | 1 530 | 494 | (32,29%) | 374 | (24,44%) | 230 | (15,03%) | 432 | (28,24%) | 360 | (26,73%) | 77 | (5,72%) | 86 | (6,38%) | 78 | (5,79%) | 741 | (55,01%) | 5 | (,37%) |
| Angola | 47 | 12 | (25,53%) | 6 | (12,77%) | 8 | (17,02%) | 21 | (44,68%) | 28 | (77,78%) | 2 | (5,56%) | 3 | (8,33%) | 2 | (5,56%) | 1 | (2,78%) | 0 | (,00%) |
| Argentina | 877 | 471 | (54,08%) | 134 | (15,38%) | 51 | (5,86%) | 215 | (24,68%) | 158 | (28,06%) | 73 | (12,97%) | 114 | (20,25%) | 47 | (8,35%) | 78 | (13,85%) | 93 | (16,52%) |
| Australia | 387 | 160 | (41,34%) | 27 | (6,98%) | 122 | (31,52%) | 78 | (20,16%) | 137 | (44,19%) | 58 | (18,71%) | 14 | (4,52%) | 37 | (11,94%) | 55 | (17,74%) | 9 | (2,90%) |
| Austria | 154 | 53 | (34,64%) | 8 | (5,23%) | 70 | (45,75%) | 22 | (14,38%) | 35 | (30,17%) | 14 | (12,07%) | 6 | (5,17%) | 4 | (3,45%) | 55 | (47,41%) | 2 | (1,72%) |
| Azerbaijan | 35 | 27 | (77,14%) | 7 | (20,00%) | 1 | (2,86%) | 0 | (,00%) | 4 | (13,33%) | 1 | (3,33%) | 1 | (3,33%) | 1 | (3,33%) | 23 | (76,67%) | 0 | (,00%) |
| Bahrain | 36 | 18 | (50,00%) | 3 | (8,33%) | 2 | (5,56%) | 13 | (36,11%) | 18 | (60,00%) | 0 | (,00%) | 4 | (13,33%) | 2 | (6,67%) | 6 | (20,00%) | 0 | (,00%) |
| Bangladesh | 30 | 21 | (70,00%) | 2 | (6,67%) | 5 | (16,67%) | 2 | (6,67%) | 2 | (8,00%) | 2 | (8,00%) | 2 | (8,00%) | 1 | (4,00%) | 18 | (72,00%) | 0 | (,00%) |
| Belarus | 100 | 51 | (51,00%) | 15 | (15,00%) | 24 | (24,00%) | 10 | (10,00%) | 27 | (32,53%) | 2 | (2,41%) | 10 | (12,05%) | 4 | (4,82%) | 40 | (48,19%) | 0 | (,00%) |
| Belgium | 1 806 | 530 | (29,35%) | 61 | (3,38%) | 519 | (28,74%) | 696 | (38,54%) | 718 | (41,36%) | 165 | (9,50%) | 75 | (4,32%) | 103 | (5,93%) | 284 | (16,36%) | 391 | (22,52%) |
| Bolivia | 38 | 21 | (55,26%) | 12 | (31,58%) | 2 | (5,26%) | 3 | (7,89%) | 5 | (16,13%) | 3 | (9,68%) | 4 | (12,90%) | 1 | (3,23%) | 18 | (58,06%) | 0 | (,00%) |
| Bosnia and Herzegovina | 293 | 142 | (48,46%) | 33 | (11,26%) | 65 | (22,18%) | 53 | (18,09%) | 80 | (29,30%) | 4 | (1,47%) | 10 | (3,66%) | 19 | (6,96%) | 160 | (58,61%) | 0 | (,00%) |
| Brazil | 1 104 | 506 | (45,87%) | 99 | (8,98%) | 309 | (28,01%) | 189 | (17,14%) | 294 | (33,41%) | 62 | (7,05%) | 148 | (16,82%) | 64 | (7,27%) | 304 | (34,55%) | 8 | (,91%) |
| Brunei | 163 | 93 | (57,06%) | 15 | (9,20%) | 25 | (15,34%) | 30 | (18,40%) | 73 | (48,67%) | 9 | (6,00%) | 10 | (6,67%) | 15 | (10,00%) | 43 | (28,67%) | 0 | (,00%) |
| Bulgaria | 229 | 73 | (31,88%) | 21 | (9,17%) | 116 | (50,66%) | 19 | (8,30%) | 46 | (33,82%) | 4 | (2,94%) | 9 | (6,62%) | 2 | (1,47%) | 75 | (55,15%) | 0 | (,00%) |
| Canada | 750 | 273 | (36,45%) | 70 | (9,35%) | 200 | (26,70%) | 206 | (27,50%) | 359 | (55,92%) | 69 | (10,75%) | 41 | (6,39%) | 69 | (10,75%) | 93 | (14,49%) | 11 | (1,71%) |
| Chile | 1 204 | 465 | (38,65%) | 357 | (29,68%) | 237 | (19,70%) | 144 | (11,97%) | 386 | (39,55%) | 73 | (7,48%) | 151 | (15,47%) | 105 | (10,76%) | 258 | (26,43%) | 3 | (,31%) |
| China | 46 | 28 | (60,87%) | 3 | (6,52%) | 11 | (23,91%) | 4 | (8,70%) | 12 | (30,77%) | 0 | (,00%) | 2 | (5,13%) | 1 | (2,56%) | 24 | (61,54%) | 0 | (,00%) |
| Colombia | 535 | 288 | (53,83%) | 143 | (26,73%) | 36 | (6,73%) | 68 | (12,71%) | 93 | (21,18%) | 14 | (3,19%) | 58 | (13,21%) | 22 | (5,01%) | 242 | (55,13%) | 10 | (2,28%) |
| Croatia | 1 637 | 691 | (42,24%) | 154 | (9,41%) | 566 | (34,60%) | 225 | (13,75%) | 421 | (28,20%) | 20 | (1,34%) | 47 | (3,15%) | 80 | (5,36%) | 884 | (59,21%) | 41 | (2,75%) |
| Cyprus | 164 | 66 | (40,24%) | 12 | (7,32%) | 51 | (31,10%) | 35 | (21,34%) | 68 | (49,64%) | 7 | (5,11%) | 9 | (6,57%) | 9 | (6,57%) | 43 | (31,39%) | 1 | (,73%) |
| Czech Republic | 846 | 292 | (34,52%) | 127 | (15,01%) | 304 | (35,93%) | 123 | (14,54%) | 181 | (28,96%) | 25 | (4,00%) | 33 | (5,28%) | 19 | (3,04%) | 361 | (57,76%) | 6 | (,96%) |
| Denmark | 290 | 107 | (36,90%) | 16 | (5,52%) | 131 | (45,17%) | 36 | (12,41%) | 38 | (18,63%) | 13 | (6,37%) | 6 | (2,94%) | 12 | (5,88%) | 133 | (65,20%) | 2 | (,98%) |
| Dominican Republic | 354 | 193 | (54,52%) | 89 | (25,14%) | 18 | (5,08%) | 54 | (15,25%) | 59 | (23,41%) | 13 | (5,16%) | 19 | (7,54%) | 14 | (5,56%) | 147 | (58,33%) | 0 | (,00%) |
| Ecuador | 1 384 | 573 | (41,49%) | 363 | (26,29%) | 106 | (7,68%) | 339 | (24,55%) | 304 | (25,35%) | 76 | (6,34%) | 199 | (16,60%) | 80 | (6,67%) | 521 | (43,45%) | 19 | (1,58%) |
| Egypt | 37 | 22 | (59,46%) | 2 | (5,41%) | 5 | (13,51%) | 8 | (21,62%) | 9 | (27,27%) | 5 | (15,15%) | 1 | (3,03%) | 2 | (6,06%) | 15 | (45,45%) | 1 | (3,03%) |
| El Salvador | 208 | 95 | (45,89%) | 80 | (38,65%) | 5 | (2,42%) | 27 | (13,04%) | 41 | (29,71%) | 5 | (3,62%) | 5 | (3,62%) | 1 | (,72%) | 86 | (62,32%) | 0 | (,00%) |
| Estonia | 365 | 135 | (36,99%) | 63 | (17,26%) | 98 | (26,85%) | 69 | (18,90%) | 122 | (49,00%) | 28 | (11,24%) | 13 | (5,22%) | 10 | (4,02%) | 72 | (28,92%) | 4 | (1,61%) |
| Finland | 588 | 175 | (29,76%) | 28 | (4,76%) | 267 | (45,41%) | 118 | (20,07%) | 189 | (41,18%) | 24 | (5,23%) | 21 | (4,58%) | 26 | (5,66%) | 190 | (41,39%) | 9 | (1,96%) |
| France | 1 590 | 711 | (44,72%) | 153 | (9,62%) | 599 | (37,67%) | 127 | (7,99%) | 349 | (26,36%) | 78 | (5,89%) | 92 | (6,95%) | 127 | (9,59%) | 666 | (50,30%) | 12 | (,91%) |
| Georgia | 469 | 311 | (66,31%) | 35 | (7,46%) | 59 | (12,58%) | 64 | (13,65%) | 80 | (22,60%) | 18 | (5,08%) | 12 | (3,39%) | 8 | (2,26%) | 236 | (66,67%) | 0 | (,00%) |
| Germany | 562 | 222 | (39,57%) | 39 | (6,95%) | 189 | (33,69%) | 111 | (19,79%) | 157 | (34,58%) | 41 | (9,03%) | 26 | (5,73%) | 37 | (8,15%) | 185 | (40,75%) | 8 | (1,76%) |
| Ghana | 289 | 75 | (26,04%) | 27 | (9,38%) | 12 | (4,17%) | 174 | (60,42%) | 144 | (52,36%) | 58 | (21,09%) | 9 | (3,27%) | 7 | (2,55%) | 38 | (13,82%) | 19 | (6,91%) |
| Greece | 825 | 352 | (42,67%) | 60 | (7,27%) | 289 | (35,03%) | 124 | (15,03%) | 218 | (32,83%) | 37 | (5,57%) | 40 | (6,02%) | 31 | (4,67%) | 336 | (50,60%) | 2 | (,30%) |
| Guatemala | 40 | 15 | (37,50%) | 8 | (20,00%) | 3 | (7,50%) | 14 | (35,00%) | 20 | (57,14%) | 2 | (5,71%) | 9 | (25,71%) | 0 | (,00%) | 4 | (11,43%) | 0 | (,00%) |
| Honduras | 570 | 284 | (49,91%) | 159 | (27,94%) | 42 | (7,38%) | 84 | (14,76%) | 103 | (22,59%) | 14 | (3,07%) | 26 | (5,70%) | 39 | (8,55%) | 269 | (58,99%) | 5 | (1,10%) |
| Hungary | 530 | 176 | (33,21%) | 30 | (5,66%) | 251 | (47,36%) | 73 | (13,77%) | 107 | (25,91%) | 8 | (1,94%) | 18 | (4,36%) | 5 | (1,21%) | 274 | (66,34%) | 1 | (,24%) |
| India | 364 | 182 | (50,00%) | 23 | (6,32%) | 56 | (15,38%) | 103 | (28,30%) | 132 | (40,37%) | 16 | (4,89%) | 54 | (16,51%) | 22 | (6,73%) | 102 | (31,19%) | 1 | (,31%) |
| Iran | 571 | 258 | (45,18%) | 92 | (16,11%) | 35 | (6,13%) | 186 | (32,57%) | 0 | (,00%) | 1 | (50,00%) | 0 | (,00%) | 0 | (,00%) | 1 | (50,00%) | 0 | (,00%) |
| Ireland | 348 | 95 | (27,30%) | 18 | (5,17%) | 93 | (26,72%) | 142 | (40,80%) | 205 | (61,19%) | 46 | (13,73%) | 14 | (4,18%) | 35 | (10,45%) | 28 | (8,36%) | 7 | (2,09%) |
| Israel | 1 012 | 333 | (32,91%) | 75 | (7,41%) | 222 | (21,94%) | 382 | (37,75%) | 322 | (44,85%) | 69 | (9,61%) | 52 | (7,24%) | 45 | (6,27%) | 201 | (27,99%) | 29 | (4,04%) |
| Italy | 3 005 | 995 | (33,12%) | 254 | (8,46%) | 1 389 | (46,24%) | 366 | (12,18%) | 682 | (25,23%) | 260 | (9,62%) | 327 | (12,10%) | 270 | (9,99%) | 1 100 | (40,70%) | 64 | (2,37%) |
| Jamaica | 41 | 19 | (46,34%) | 2 | (4,88%) | 5 | (12,20%) | 15 | (36,59%) | 19 | (59,38%) | 4 | (12,50%) | 5 | (15,63%) | 0 | (,00%) | 4 | (12,50%) | 0 | (,00%) |
| Japan | 1 910 | 809 | (42,38%) | 237 | (12,41%) | 12 | (,63%) | 851 | (44,58%) | 808 | (44,47%) | 296 | (16,29%) | 194 | (10,68%) | 200 | (11,01%) | 170 | (9,36%) | 149 | (8,20%) |
| Jordan | 33 | 24 | (72,73%) | 1 | (3,03%) | 4 | (12,12%) | 4 | (12,12%) | 11 | (36,67%) | 2 | (6,67%) | 2 | (6,67%) | 4 | (13,33%) | 11 | (36,67%) | 0 | (,00%) |
| Kazakhstan | 491 | 254 | (51,73%) | 51 | (10,39%) | 52 | (10,59%) | 134 | (27,29%) | 179 | (47,35%) | 23 | (6,08%) | 26 | (6,88%) | 19 | (5,03%) | 127 | (33,60%) | 4 | (1,06%) |
| Kenya | 316 | 147 | (46,52%) | 92 | (29,11%) | 43 | (13,61%) | 34 | (10,76%) | 41 | (15,71%) | 41 | (15,71%) | 43 | (16,48%) | 52 | (19,92%) | 84 | (32,18%) | 0 | (,00%) |
| Kuwait | 51 | 30 | (58,82%) | 5 | (9,80%) | 8 | (15,69%) | 8 | (15,69%) | 25 | (53,19%) | 0 | (,00%) | 3 | (6,38%) | 2 | (4,26%) | 17 | (36,17%) | 0 | (,00%) |
| Lebanon | 116 | 56 | (48,28%) | 11 | (9,48%) | 22 | (18,97%) | 27 | (23,28%) | 35 | (36,84%) | 6 | (6,32%) | 9 | (9,47%) | 12 | (12,63%) | 32 | (33,68%) | 1 | (1,05%) |
| Lithuania | 657 | 272 | (41,40%) | 96 | (14,61%) | 184 | (28,01%) | 105 | (15,98%) | 163 | (36,63%) | 33 | (7,42%) | 22 | (4,94%) | 18 | (4,04%) | 207 | (46,52%) | 2 | (,45%) |
| Macedonia | 1 062 | 385 | (36,25%) | 81 | (7,63%) | 365 | (34,37%) | 231 | (21,75%) | 385 | (40,87%) | 34 | (3,61%) | 50 | (5,31%) | 75 | (7,96%) | 392 | (41,61%) | 6 | (,64%) |
| Malaysia | 985 | 598 | (60,71%) | 157 | (15,94%) | 84 | (8,53%) | 146 | (14,82%) | 156 | (17,41%) | 27 | (3,01%) | 57 | (6,36%) | 87 | (9,71%) | 561 | (62,61%) | 8 | (,89%) |
| Mexico | 339 | 157 | (46,31%) | 97 | (28,61%) | 32 | (9,44%) | 53 | (15,63%) | 78 | (27,66%) | 26 | (9,22%) | 46 | (16,31%) | 28 | (9,93%) | 103 | (36,52%) | 1 | (,35%) |
| Moldova | 34 | 16 | (47,06%) | 1 | (2,94%) | 11 | (32,35%) | 6 | (17,65%) | 3 | (11,54%) | 1 | (3,85%) | 2 | (7,69%) | 1 | (3,85%) | 19 | (73,08%) | 0 | (,00%) |
| Montenegro | 37 | 26 | (70,27%) | 2 | (5,41%) | 5 | (13,51%) | 4 | (10,81%) | 10 | (31,25%) | 0 | (,00%) | 1 | (3,13%) | 4 | (12,50%) | 17 | (53,13%) | 0 | (,00%) |
| Morocco | 2 128 | 572 | (26,88%) | 519 | (24,39%) | 496 | (23,31%) | 541 | (25,42%) | 524 | (30,11%) | 11 | (,63%) | 154 | (8,85%) | 340 | (19,54%) | 711 | (40,86%) | 0 | (,00%) |
| NA | 172 | 59 | (43,70%) | 11 | (8,15%) | 32 | (23,70%) | 33 | (24,44%) | 45 | (36,59%) | 4 | (3,25%) | 8 | (6,50%) | 9 | (7,32%) | 52 | (42,28%) | 5 | (4,07%) |
| Netherlands | 488 | 150 | (30,74%) | 22 | (4,51%) | 107 | (21,93%) | 209 | (42,83%) | 160 | (35,16%) | 84 | (18,46%) | 20 | (4,40%) | 61 | (13,41%) | 36 | (7,91%) | 94 | (20,66%) |
| New Zealand | 309 | 113 | (36,57%) | 11 | (3,56%) | 73 | (23,62%) | 112 | (36,25%) | 108 | (37,11%) | 54 | (18,56%) | 29 | (9,97%) | 45 | (15,46%) | 12 | (4,12%) | 43 | (14,78%) |
| Nigeria | 627 | 469 | (74,92%) | 89 | (14,22%) | 36 | (5,75%) | 32 | (5,11%) | 16 | (2,90%) | 5 | (,91%) | 10 | (1,81%) | 2 | (,36%) | 518 | (94,01%) | 0 | (,00%) |
| Norway | 1 312 | 468 | (35,67%) | 63 | (4,80%) | 367 | (27,97%) | 414 | (31,55%) | 600 | (61,29%) | 88 | (8,99%) | 25 | (2,55%) | 34 | (3,47%) | 181 | (18,49%) | 51 | (5,21%) |
| Pakistan | 225 | 161 | (71,56%) | 6 | (2,67%) | 19 | (8,44%) | 39 | (17,33%) | 41 | (19,71%) | 6 | (2,88%) | 8 | (3,85%) | 12 | (5,77%) | 141 | (67,79%) | 0 | (,00%) |
| Peru | 48 | 21 | (43,75%) | 14 | (29,17%) | 9 | (18,75%) | 4 | (8,33%) | 9 | (22,50%) | 4 | (10,00%) | 8 | (20,00%) | 5 | (12,50%) | 14 | (35,00%) | 0 | (,00%) |
| Philippines | 1 763 | 951 | (53,94%) | 201 | (11,40%) | 454 | (25,75%) | 157 | (8,91%) | 338 | (20,95%) | 82 | (5,08%) | 68 | (4,22%) | 92 | (5,70%) | 1 020 | (63,24%) | 13 | (,81%) |
| Poland | 5 535 | 1 968 | (35,56%) | 598 | (10,80%) | 2 161 | (39,04%) | 808 | (14,60%) | 1 198 | (31,37%) | 247 | (6,47%) | 260 | (6,81%) | 171 | (4,48%) | 1 905 | (49,88%) | 38 | (1,00%) |
| Portugal | 1 395 | 562 | (40,34%) | 361 | (25,92%) | 234 | (16,80%) | 236 | (16,94%) | 411 | (33,86%) | 37 | (3,05%) | 104 | (8,57%) | 69 | (5,68%) | 587 | (48,35%) | 6 | (,49%) |
| Romania | 788 | 280 | (35,53%) | 53 | (6,73%) | 337 | (42,77%) | 118 | (14,97%) | 165 | (25,31%) | 6 | (,92%) | 47 | (7,21%) | 11 | (1,69%) | 417 | (63,96%) | 6 | (,92%) |
| Russia | 2 856 | 1 154 | (40,42%) | 426 | (14,92%) | 602 | (21,09%) | 673 | (23,57%) | 818 | (35,96%) | 123 | (5,41%) | 235 | (10,33%) | 142 | (6,24%) | 934 | (41,05%) | 23 | (1,01%) |
| Serbia | 1 297 | 575 | (44,33%) | 173 | (13,34%) | 368 | (28,37%) | 181 | (13,96%) | 328 | (28,37%) | 24 | (2,08%) | 72 | (6,23%) | 64 | (5,54%) | 661 | (57,18%) | 7 | (,61%) |
| Slovakia | 935 | 395 | (42,25%) | 113 | (12,09%) | 352 | (37,65%) | 75 | (8,02%) | 119 | (15,93%) | 15 | (2,01%) | 23 | (3,08%) | 19 | (2,54%) | 568 | (76,04%) | 3 | (,40%) |
| Slovenia | 682 | 231 | (33,87%) | 45 | (6,60%) | 268 | (39,30%) | 138 | (20,23%) | 325 | (49,85%) | 18 | (2,76%) | 53 | (8,13%) | 42 | (6,44%) | 194 | (29,75%) | 20 | (3,07%) |
| South Africa | 109 | 42 | (38,53%) | 15 | (13,76%) | 33 | (30,28%) | 19 | (17,43%) | 26 | (28,89%) | 9 | (10,00%) | 10 | (11,11%) | 9 | (10,00%) | 36 | (40,00%) | 0 | (,00%) |
| South Korea | 835 | 235 | (28,14%) | 115 | (13,77%) | 34 | (4,07%) | 451 | (54,01%) | 429 | (54,51%) | 73 | (9,28%) | 79 | (10,04%) | 119 | (15,12%) | 57 | (7,24%) | 30 | (3,81%) |
| Spain | 205 | 88 | (42,93%) | 36 | (17,56%) | 34 | (16,59%) | 47 | (22,93%) | 78 | (45,35%) | 17 | (9,88%) | 13 | (7,56%) | 20 | (11,63%) | 40 | (23,26%) | 4 | (2,33%) |
| Sweden | 341 | 129 | (37,83%) | 27 | (7,92%) | 133 | (39,00%) | 52 | (15,25%) | 112 | (42,11%) | 30 | (11,28%) | 9 | (3,38%) | 28 | (10,53%) | 73 | (27,44%) | 14 | (5,26%) |
| Switzerland | 420 | 138 | (32,94%) | 14 | (3,34%) | 191 | (45,58%) | 76 | (18,14%) | 96 | (29,27%) | 74 | (22,56%) | 15 | (4,57%) | 9 | (2,74%) | 97 | (29,57%) | 37 | (11,28%) |
| Taiwan | 409 | 200 | (48,90%) | 13 | (3,18%) | 118 | (28,85%) | 78 | (19,07%) | 214 | (58,63%) | 19 | (5,21%) | 24 | (6,58%) | 20 | (5,48%) | 80 | (21,92%) | 8 | (2,19%) |
| Thailand | 248 | 168 | (67,74%) | 68 | (27,42%) | 4 | (1,61%) | 8 | (3,23%) | 9 | (4,11%) | 0 | (,00%) | 8 | (3,65%) | 12 | (5,48%) | 190 | (86,76%) | 0 | (,00%) |
| Tunisia | 628 | 317 | (50,56%) | 94 | (14,99%) | 64 | (10,21%) | 152 | (24,24%) | 171 | (29,03%) | 74 | (12,56%) | 157 | (26,66%) | 70 | (11,88%) | 108 | (18,34%) | 9 | (1,53%) |
| Türkiye | 7 291 | 3 324 | (45,61%) | 820 | (11,25%) | 2 079 | (28,53%) | 1 065 | (14,61%) | 1 449 | (21,79%) | 156 | (2,35%) | 266 | (4,00%) | 513 | (7,71%) | 4 151 | (62,42%) | 115 | (1,73%) |
| Uganda | 331 | 114 | (34,55%) | 107 | (32,42%) | 25 | (7,58%) | 84 | (25,45%) | 46 | (17,97%) | 29 | (11,33%) | 52 | (20,31%) | 29 | (11,33%) | 96 | (37,50%) | 4 | (1,56%) |
| UK | 671 | 281 | (41,88%) | 57 | (8,49%) | 238 | (35,47%) | 95 | (14,16%) | 202 | (34,53%) | 42 | (7,18%) | 35 | (5,98%) | 34 | (5,81%) | 268 | (45,81%) | 4 | (,68%) |
| Ukraine | 1 422 | 653 | (45,92%) | 271 | (19,06%) | 289 | (20,32%) | 209 | (14,70%) | 239 | (25,11%) | 36 | (3,78%) | 92 | (9,66%) | 29 | (3,05%) | 553 | (58,09%) | 3 | (,32%) |
| United Arab Emirates | 40 | 15 | (37,50%) | 5 | (12,50%) | 4 | (10,00%) | 16 | (40,00%) | 20 | (52,63%) | 1 | (2,63%) | 3 | (7,89%) | 2 | (5,26%) | 12 | (31,58%) | 0 | (,00%) |
| Uruguay | 482 | 207 | (43,04%) | 129 | (26,82%) | 88 | (18,30%) | 57 | (11,85%) | 87 | (31,87%) | 31 | (11,36%) | 29 | (10,62%) | 9 | (3,30%) | 116 | (42,49%) | 1 | (,37%) |
| USA | 3 713 | 1 324 | (35,66%) | 286 | (7,70%) | 703 | (18,93%) | 1 400 | (37,71%) | 1 750 | (54,96%) | 294 | (9,23%) | 284 | (8,92%) | 291 | (9,14%) | 395 | (12,41%) | 170 | (5,34%) |
| Uzbekistan | 182 | 72 | (39,56%) | 22 | (12,09%) | 30 | (16,48%) | 58 | (31,87%) | 38 | (32,48%) | 11 | (9,40%) | 9 | (7,69%) | 11 | (9,40%) | 47 | (40,17%) | 1 | (,85%) |
| Venezuela | 553 | 314 | (56,78%) | 136 | (24,59%) | 53 | (9,58%) | 50 | (9,04%) | 96 | (26,52%) | 27 | (7,46%) | 73 | (20,17%) | 21 | (5,80%) | 136 | (37,57%) | 9 | (2,49%) |
| Total | 69 924 | 28 627 | (40,98%) | 9 050 | (12,96%) | 17 685 | (25,32%) | 14 494 | (20,75%) | 18 966 | (32,86%) | 3 720 | (6,44%) | 4 522 | (7,83%) | 4 302 | (7,45%) | 24 560 | (42,55%) | 1 652 | (2,86%) |

Table S2. Descriptive statistics along languages.

|  | N | Men | Women | M_Age_ | SD_Age_ | Median_Age_ | Range_Age_ |
| --- | --- | --- | --- | --- | --- | --- | --- |
| AR | 4425 | 2002 | 2409 | 28.62 | 7.51 | 27 | 18-80 |
| BG | 213 | 35 | 177 | 24.64 | 6.26 | 22 | 19-50 |
| BS | 246 | 50 | 195 | 27.43 | 9.58 | 23 | 18-55 |
| CS | 760 | 204 | 551 | 27.09 | 9.17 | 24 | 18-77 |
| DE | 769 | 217 | 537 | 32.65 | 14.50 | 28 | 18-90 |
| EL | 853 | 121 | 723 | 28.67 | 10.87 | 24 | 18-67 |
| EN | 11896 | 4985 | 6687 | 30.85 | 12.33 | 27 | 18-89 |
| ES | 6816 | 2483 | 4229 | 30.05 | 11.83 | 26 | 18-90 |
| ET | 355 | 54 | 297 | 33.62 | 11.95 | 33 | 18-76 |
| FA | 587 | 184 | 397 | 30.56 | 9.07 | 29 | 18-67 |
| FI | 444 | 64 | 353 | 32.60 | 10.92 | 29 | 18-77 |
| FIL | 1298 | 339 | 882 | 24.80 | 8.97 | 21 | 18-67 |
| FR | 2018 | 578 | 1392 | 27.00 | 10.17 | 23 | 18-82 |
| HE | 975 | 375 | 597 | 33.57 | 13.37 | 28 | 18-77 |
| HR | 1687 | 387 | 1276 | 27.48 | 11.21 | 22 | 18-78 |
| HU | 516 | 99 | 406 | 25.99 | 9.23 | 22 | 18-65 |
| IT | 2995 | 867 | 2103 | 30.99 | 12.10 | 26 | 18-77 |
| JA | 1903 | 1003 | 865 | 41.02 | 11.72 | 43 | 18-75 |
| KA | 462 | 88 | 364 | 24.45 | 10.46 | 20 | 18-73 |
| KO | 824 | 379 | 444 | 42.70 | 13.18 | 41.5 | 18-69 |
| LT | 671 | 179 | 483 | 27.17 | 10.70 | 22 | 18-67 |
| MK | 964 | 338 | 616 | 28.04 | 10.66 | 23 | 18-83 |
| MS | 244 | 93 | 151 | 28.82 | 11.00 | 25 | 18-72 |
| NL | 1781 | 804 | 972 | 49.50 | 15.99 | 50 | 18-90 |
| NO | 702 | 133 | 569 | 37.30 | 12.59 | 36 | 18-80 |
| PL | 5602 | 1395 | 4131 | 26.10 | 9.29 | 23 | 18-89 |
| PT | 2486 | 954 | 1498 | 30.40 | 10.57 | 27 | 18-72 |
| RO | 847 | 331 | 513 | 25.47 | 9.63 | 21 | 18-73 |
| RU | 3976 | 1180 | 2757 | 26.87 | 10.37 | 22 | 18-89 |
| SK | 1002 | 233 | 757 | 24.13 | 6.74 | 22 | 18-65 |
| SL | 666 | 127 | 531 | 35.78 | 12.83 | 34 | 18-81 |
| SR | 1423 | 381 | 1033 | 26.76 | 9.85 | 22 | 18-66 |
| SV | 368 | 81 | 276 | 32.51 | 11.78 | 29 | 18-76 |
| TH | 244 | 138 | 95 | 21.13 | 4.06 | 20 | 18-49 |
| TR | 7516 | 2319 | 5115 | 26.54 | 10.28 | 23 | 18-90 |
| UK | 1333 | 256 | 1063 | 23.01 | 7.77 | 20 | 18-72 |
| ZH | 529 | 231 | 284 | 31.01 | 9.00 | 30 | 18-66 |

*Note*: AR = Arabic, BG = Bulgarian, BS = Bosnian, CS = Czech, DE = German, EL = Greek, EN = English, ES = Spanish, ET = Estonian, FA = Persian, FI = Finnish, FIL = Filipino, FR = French, HE = Hebrew, HR = Croatian, HU = Hungarian, IT = Italian, JA = Japanese, KA = Georgian, KO = Korean, LT = Lithuanian, MK = Macedonian, MS = Malay, NL = Dutch, NO = Norwegian, PL = Polish, PT = Portuguese, RO = Romanian, RU = Russian, SK = Slovakian, SL = Slovenian, SR = Serbian, SV = Swedish, TH = Thailand, TR = Turkish, UK = Ukrainian, ZH = Chinese Traditional

Table S3. Reliability coefficients (McDonald's ω) of scales used in the study along languages.

|  | ASR-YWMS | ASR-OMWS | SOI3 | Individualism | GEMS | Parasite |
| --- | --- | --- | --- | --- | --- | --- |
| AR | .94 | .95 | .69 | .77 | .59 | .55 |
| BG | .92 | .92 | .68 | .79 | .75 | * |
| BS | .93 | .91 | .95 | .79 | .77 | * |
| CS | .92 | .89 | .70 | .77 | .79 | * |
| DE | .90 | .90 | .68 | .75 | .82 | * |
| EL | .91 | .93 | .78 | .82 | .78 | * |
| EN | .94 | .94 | .70 | .80 | .90 | .84 |
| ES | .93 | .93 | .70 | .76 | .83 | .57 |
| ET | .91 | .91 | .72 | .78 | .75 | * |
| FA | .87 | .92 | .59 | .83 | .79 | .96 |
| FI | .91 | .89 | .79 | .78 | .80 | * |
| FIL | .93 | .92 | .64 | .74 | .90 | * |
| FR | .91 | .90 | .71 | .79 | .82 | .73 |
| HE | .92 | .93 | .73 | .79 | .87 | .95 |
| HR | .93 | .92 | .68 | .83 | .82 | .91 |
| HU | .93 | .92 | .74 | .81 | .80 | * |
| IT | .92 | .93 | .70 | .84 | .86 | .90 |
| JA | .92 | .94 | .75 | .77 | .87 | .97 |
| KA | .93 | .91 | .68 | .75 | .74 | .94 |
| KO | .89 | .99 | .72 | .82 | .88 | .78 |
| LT | .93 | .93 | .67 | .77 | .80 | .91 |
| MK | .92 | .94 | .77 | .78 | .84 | .98 |
| MS | .93 | .95 | .79 | .82 | .79 | * |
| NL | .93 | .94 | .74 | .81 | .87 | .94 |
| NO | .92 | .94 | .72 | .75 | .75 | * |
| PL | .94 | .93 | .72 | .83 | .81 | .91 |
| PT | .92 | .93 | .72 | .83 | .83 | .19 |
| RO | .94 | .95 | .73 | .81 | .86 | .97 |
| RU | .93 | .94 | .66 | .74 | .75 | .85 |
| SK | .91 | .90 | .66 | .72 | .74 | .33 |
| SL | .91 | .92 | .80 | .81 | .78 | .97 |
| SR | .93 | .93 | .72 | .82 | .80 | .95 |
| SV | .93 | .91 | .69 | .82 | .85 | .97 |
| TH | .93 | .94 | .68 | .74 | .83 | .80 |
| TR | .94 | .95 | .75 | .83 | .83 | .86 |
| UK | .92 | .92 | .65 | .72 | .80 | .96 |
| ZH | .93 | .93 | .75 | .78 | .87 | .95 |

*Note*: AR = Arabic, BG = Bulgarian, BS = Bosnian, CS = Czech, DE = German, EL = Greek, EN = English, ES = Spanish, ET = Estonian, FA = Persian, FI = Finnish, FIL = Filipino, FR = French, HE = Hebrew, HR = Croatian, HU = Hungarian, IT = Italian, JA = Japanese, KA = Georgian, KO = Korean, LT = Lithuanian, MK = Macedonian, MS = Malay, NL = Dutch, NO = Norwegian, PL = Polish, PT = Portuguese, RO = Romanian, RU = Russian, SK = Slovakian, SL = Slovenian, SR = Serbian, SV = Swedish, TH = Thailand, TR = Turkish, UK = Ukrainian, ZH = Chinese Traditional; ASR-YWMS = Acceptance of Sugar Relationships in Young Women and Men Scale; ASR-OMWS = Acceptance of Sugar Relationships in Older Menand Women Scale; SOI3 - Three-Item Sociosexual Orientation Inventory; Individualism = Collectivism Scale; GEMS = Gender-Equitable Men Scale; Parasite = Nine-item Pathogen Prevalence Index; * = Each of the component variables has zero variance and is removed from the scale or the determinant of the covariance matrix is zero or approximately zero.

Language: Arab

**Country: Bahrain, Egypt, Jordan, United Arab Emirates**

**علاقة السكر:**  إن علاقة السكر هي علاقة جنسية تبادلية، نجد فيها شريك كبير السن وغني (سكر دادي/ سكرمامي) يوفر موارد مادية لشريك أصغر منه سنًا (سكر بايبي/بوي) مقابل مرافقته / مرافقتها. وعادة ما يجتمع الشركاء للتمتع بوقت الفراغ معًا، أما النشاط الجنسي فلا يتم إلا إذا وافق كلا الشريكين على ذلك.   من فضلك قيم العبارات التالية **كما لو كنت أحد "سكر بايبي/بوي"** , أي فرد يتلقى موارد مادية مقابل خدمة المرافقة التي يقدمه أو تقدمها. من فضلك وضح مدى موافقتك على كل من العبارات الواردة أدناه، وهذا باستخدام سلم القياس المكون من سبع نقاط والتي تتراوح من (1) “"لا أوافق تماما" ” إلى (7) “"أوافق تمامًا".”.

| أوافق تمامًا | 7 = | 6 | 5 | 4 | 3 | 2 | = 1 | لا أوافق تمامًا |
| --- | --- | --- | --- | --- | --- | --- | --- | --- |
|  |  |  |  |  |  |  |  |  |

| \| 7 \| 6 \| 5 \| 4 \| 3 \| 2 \| 1 \| \| --- \| --- \| --- \| --- \| --- \| --- \| --- \| | تعتبر علاقة السكر أمرًا جيدًا لأنها يمكن أن تساعد "السكر بيبي/بوي" في الحصول على وضع مالي مُرض. |
| --- | --- | --- | --- | --- | --- | --- | --- | --- |
| \| **7** \| **6** \| **5** \| **4** \| **3** \| **2** \| **1** \| \| --- \| --- \| --- \| --- \| --- \| --- \| --- \| | في المستقبل، قد ينتهي الأمر بي مندمج في علاقة سكر. |
| \| 7 \| 6 \| 5 \| 4 \| 3 \| 2 \| 1 \| \| --- \| --- \| --- \| --- \| --- \| --- \| --- \| | لو أعرف أنه لن أتعرض لأحكام سلبية أو عواقب سلبية، فلن يكون لدي مانع من محاولة إقامة علاقة سكر. |
| \| 7 \| 6 \| 5 \| 4 \| 3 \| 2 \| 1 \| \| --- \| --- \| --- \| --- \| --- \| --- \| --- \| | إذا كانت ستفيد مسيرتي، سأفكر في الدخول في علاقة سكر. |
| \| *7* \| *6* \| *5* \| *4* \| *3* \| *2* \| *1* \| \| --- \| --- \| --- \| --- \| --- \| --- \| --- \| | سأفكر بجدية في الدخول في علاقة سكر إذا تبين لي أنها ستساعدني في الحصول على وضع مالي أحسن. |

العبارات التالية قد تبدو متشابهة. ولكن الآن، من فضلك قيم العبارات التالية كما لو كنت أحد "سكر دادي/مامي" أي الفرد الذي يوفر موارد مادية لرفيقته/رفيقها. من فضلك وضح مدى موافقتك على كل من العبارات الواردة أدناه، وهذا باستخدام سلم القياس المكون من سبع نقاط والتي تتراوح من (1) “ "لا أوافق تماما" ” إلى (7) “ "أوافق تمامًا" ”

| أوافق تمامًا | 7 = | 6 | 5 | 4 | 3 | 2 | = 1 | لا أوافق تمامًا |
| --- | --- | --- | --- | --- | --- | --- | --- | --- |

| \| 7 \| 6 \| 5 \| 4 \| 3 \| 2 \| 1 \| \| --- \| --- \| --- \| --- \| --- \| --- \| --- \| | تعتبر علاقة السكر أمرًا جيدًا لأنها يمكن أن تساعد "الناس" في الحصول على شعور أفضل. |
| --- | --- | --- | --- | --- | --- | --- | --- | --- |
| \| 7 \| 6 \| 5 \| 4 \| 3 \| 2 \| 1 \| \| --- \| --- \| --- \| --- \| --- \| --- \| --- \| | في المستقبل، قد ينتهي الأمر بي مندمج في علاقة سكر. |
| \| 7 \| 6 \| 5 \| 4 \| 3 \| 2 \| 1 \| \| --- \| --- \| --- \| --- \| --- \| --- \| --- \| | لو أعرف أنه لن أتعرض لأحكام سلبية أو عواقب سلبية، فلن يكون لدي مانع من محاولة إقامة علاقة سكر. |
| \| 7 \| 6 \| 5 \| 4 \| 3 \| 2 \| 1 \| \| --- \| --- \| --- \| --- \| --- \| --- \| --- \| | إذا كانت ستفيد مسيرتي، سأفكر في الدخول في علاقة سكر. |
| \| 7 \| 6 \| 5 \| 4 \| 3 \| 2 \| 1 \| \| --- \| --- \| --- \| --- \| --- \| --- \| --- \| | سأفكر بجدية في الدخول في علاقة سكر إذا تبين لي أنها ستساعدني في الحصول على وضع مالي أحسن |

Language: Bosnian

**Country: Bosnia-Hercegovina, Montenegro**

**"Sponzorska" veza** je dogovorni seksualni odnos u kojem starija i bogatija osoba/partner (tzv. sponzor ili sponzorica, a na engleskom: sugar daddy ili sugar mommy) osigurava materijalna dobra mlađoj osobi/partneru (eng. sugar baby / sugar boy) u zamjenu za njegovo ili njeno društvo. Partneri se obično zajedno druže u slobodno vrijeme, a seksualna aktivnost je izraz obostrane volje.  Molimo da odgovorite na iduće izjave **kao da ste u ulozi osobe koja prima odgovarajuće materijalne povlastice**, u zamjenu za njeno društvo (kao da ste u ulozi tzv. sugar baby / sugar boy). Molimo Vas da označite u kojoj mjeri se slažete sa svakom izjavom koristeći se odgovorima od 1 do 7, pri čemu broj 1 označava - “Uopće se ne slažem” do (7) “U potpunosti se slažem”.

| Uopće se ne slažem = | 1 | 2 | 3 | 4 | 5 | 6 | 7 | = U potpunosti se slažem |
| --- | --- | --- | --- | --- | --- | --- | --- | --- |

| Sponzorska veza je dobra stvar jer može pomoći toj mlađoj osobi, koja prima materijalne povlastice za društvo (u ovom odnosu sugar baby / sugar boy), da ima zadovoljavajuću financijsku situaciju. | \| 1 \| 2 \| 3 \| 4 \| 5 \| 6 \| 7 \| \| --- \| --- \| --- \| --- \| --- \| --- \| --- \| |
| --- | --- | --- | --- | --- | --- | --- | --- | --- |
| Mogao/mogla bih u budućnosti započeti "sponzorsku" vezu. | \| 1 \| 2 \| 3 \| 4 \| 5 \| 6 \| 7 \| \| --- \| --- \| --- \| --- \| --- \| --- \| --- \| |
| Kada bih znao/znala da neću na sebe navući negativne komentare ili posljedice, htio/htjela bih isprobati "sponzorsku" vezu. | \| 1 \| 2 \| 3 \| 4 \| 5 \| 6 \| 7 \| \| --- \| --- \| --- \| --- \| --- \| --- \| --- \| |
| Ako bi to koristilo mojoj karijeri, razmislio/la bih o započinjanju "sponzorske" veze. | \| 1 \| 2 \| 3 \| 4 \| 5 \| 6 \| 7 \| \| --- \| --- \| --- \| --- \| --- \| --- \| --- \| |
| Ozbiljno bih razmotrio/la mogućnost započinjanja "sponzorske" veze, ako bi mi to pomoglo imati bolju financijsku situaciju. | \| 1 \| 2 \| 3 \| 4 \| 5 \| 6 \| 7 \| \| --- \| --- \| --- \| --- \| --- \| --- \| --- \| |

Iduće izjave možda izgledaju slično kao prethodne. No ovaj puta Vas molimo da odgovorite na sljedeće tvrdnje **kao da ste u ulozi osobe koja osigurava materijalna dobra**  u zamjenu za druženje s partnerom (tj. kao da ste u ulozi tzv. sugar daddy / sugar mommy). Molimo Vas da označite koliko se slažete sa svakom od idućih izjava koristeći se brojevima na skali od 1 do 7, pri čemu 1 označava odgovor “Uopće se ne slažem” do 7 “U potpunosti se slažem”.

| Uopće se ne slažem = | 1 | 2 | 3 | 4 | 5 | 6 | 7 | = U potpunosti se slažem |
| --- | --- | --- | --- | --- | --- | --- | --- | --- |

| Sponzorska veza je dobra stvar jer može pomoći ljudima da se bolje osjećaju. | \| 1 \| 2 \| 3 \| 4 \| 5 \| 6 \| 7 \| \| --- \| --- \| --- \| --- \| --- \| --- \| --- \| |
| --- | --- | --- | --- | --- | --- | --- | --- | --- |
| Mogao/mogla bih u budućnosti započeti "sponzorsku" vezu. | \| 1 \| 2 \| 3 \| 4 \| 5 \| 6 \| 7 \| \| --- \| --- \| --- \| --- \| --- \| --- \| --- \| |
| Kada bih znao/znala da neću na sebe navući negativne komentare ili posljedice, htio/htjela bih isprobati "sponzorsku" vezu. | \| 1 \| 2 \| 3 \| 4 \| 5 \| 6 \| 7 \| \| --- \| --- \| --- \| --- \| --- \| --- \| --- \| |
| Ako bi to doprinijelo mom seksualnom životu ili mom imidžu, razmislio/la bih o započinjanju "sponzorske" veze . | \| 1 \| 2 \| 3 \| 4 \| 5 \| 6 \| 7 \| \| --- \| --- \| --- \| --- \| --- \| --- \| --- \| |
| Ozbiljno bih razmotrio/la mogućnost započinjenja "sponzorske" veze, ako bi na taj način pronašao/la partnera/partnericu koji/a odgovara svim mojim potrebama. | \| 1 \| 2 \| 3 \| 4 \| 5 \| 6 \| 7 \| \| --- \| --- \| --- \| --- \| --- \| --- \| --- \| |

Language: Brazilian Portuguese

**Country: Brazil**

Um **relacionamento açucarado (*sugar relationship*)** é uma relação sexual de troca/permuta na qual um(a) parceiro(a) mais velho(a) e mais rico(a) (*sugar daddy / sugar mommy*) fornece recursos materiais a um(a) parceiro(a) mais jovem (*sugar baby/boy*) em troca de sua companhia. Os parceiros geralmente se encontram para passar momentos de lazer juntos, e a atividade sexual só é envolvida se ambos os parceiros derem o seu consentimento. Avalie as seguintes afirmações **como se você fosse um sugar baby/boy**, ou seja, uma pessoa que recebe recursos materiais em troca de sua companhia. Indique até que ponto você concorda com cada uma das afirmações abaixo usando as escalas de avaliação de sete pontos que variam de (1) “discordo totalmente”a (7) “concordo totalmente”.

| Discordo totalmente = | 1 | 2 | 3 | 4 | 5 | 6 | 7 | = Concordo totalmente |
| --- | --- | --- | --- | --- | --- | --- | --- | --- |

| Um relacionamento açucarado (sugar relationship) é uma coisa boa porque pode ajudar a(o) sugar baby/boy a ter uma situação financeira satisfatória. | \| 1 \| 2 \| 3 \| 4 \| 5 \| 6 \| 7 \| \| --- \| --- \| --- \| --- \| --- \| --- \| --- \| |
| --- | --- | --- | --- | --- | --- | --- | --- | --- |
| No futuro, posso acabar tendo um relacionamento açucarado (sugar relationship). | \| 1 \| 2 \| 3 \| 4 \| 5 \| 6 \| 7 \| \| --- \| --- \| --- \| --- \| --- \| --- \| --- \| |
| Se eu soubesse que não fosse acarretar julgamentos ou consequências negativas, gostaria de tentar um relacionamento açucarado (sugar relationship). | \| 1 \| 2 \| 3 \| 4 \| 5 \| 6 \| 7 \| \| --- \| --- \| --- \| --- \| --- \| --- \| --- \| |
| Se isso beneficiasse minha carreira, eu pensaria em ter um relacionamento açucarado (sugar relationship). | \| 1 \| 2 \| 3 \| 4 \| 5 \| 6 \| 7 \| \| --- \| --- \| --- \| --- \| --- \| --- \| --- \| |
| Eu consideraria seriamente me envolver em um relacionamento açucarado (sugar relationship) se achasse que isso me ajudaria a ter uma situação financeira melhor. | \| 1 \| 2 \| 3 \| 4 \| 5 \| 6 \| 7 \| \| --- \| --- \| --- \| --- \| --- \| --- \| --- \| |

As seguintes declarações podem parecer semelhantes. Mas agora, classifique as seguintes afirmações **como se você fosse um(a) *sugardaddy/sugar mommy***, ou seja, uma pessoa que fornece recursos materiais para a companhia de seu parceiro(a). Indique até que ponto você concorda com cada uma das afirmações abaixo usando as escalas de avaliação de sete pontos que variam de (1) “discordo totalmente” a (7) “concordo totalmente”.

| Discordo totalmente = | 1 | 2 | 3 | 4 | 5 | 6 | 7 | = Concordo totalmente |
| --- | --- | --- | --- | --- | --- | --- | --- | --- |

| Um relacionamento açucarado (sugar relationship) é uma coisa boa porque pode ajudar as pessoas a se sentirem melhor. | \| 1 \| 2 \| 3 \| 4 \| 5 \| 6 \| 7 \| \| --- \| --- \| --- \| --- \| --- \| --- \| --- \| |
| --- | --- | --- | --- | --- | --- | --- | --- | --- |
| No futuro, posso acabar tendo um relacionamento açucarado (sugar relationship) | \| 1 \| 2 \| 3 \| 4 \| 5 \| 6 \| 7 \| \| --- \| --- \| --- \| --- \| --- \| --- \| --- \| |
| Se eu soubesse que não fosse acarretar julgamentos ou consequências negativas, gostaria de tentar um relacionamento açucarado (sugar relationship). | \| 1 \| 2 \| 3 \| 4 \| 5 \| 6 \| 7 \| \| --- \| --- \| --- \| --- \| --- \| --- \| --- \| |
| Se fosse benéfico para a minha vida sexual ou para o julgamento dos outros sobre mim, consideraria envolver-me em um relacionamento açucarado (sugar relationship). | \| 1 \| 2 \| 3 \| 4 \| 5 \| 6 \| 7 \| \| --- \| --- \| --- \| --- \| --- \| --- \| --- \| |
| Eu consideraria seriamente ter um relacionamento açucarado (sugar relationship) se essa fosse a maneira de encontrar um(a) parceiro(a) que atendesse a todas as minhas necessidades. | \| 1 \| 2 \| 3 \| 4 \| 5 \| 6 \| 7 \| \| --- \| --- \| --- \| --- \| --- \| --- \| --- \| |

Language: Bulgarian

**Country: Bulgaria**

**Връзката с облаги** е сексуална връзка, при която по-възрастен и по-богат партньор (татенце/мамче) предоставя материални ресурси на по-млад партньор (бебче/момче) в замяна на нейната или неговата компания. Партньорите обикновено се срещат, за да прекарват свободното си време заедно, а сексуалната активност се включва само ако и двамата партньори дадат съгласието си. Моля, оценете следните твърдения така, **сякаш сте бебче/момче**, т.е. човек, който получава материални ресурси за своята компания. Моля, посочете степента, в която се съгласявате с всяко от изложените по-долу твърдения, използвайки 7-степенните рейтингови скали, вариращи от (1) "напълно несъгласен" до (7) "напълно съгласен".

| напълно несъгласен = | 1 | 2 | 3 | 4 | 5 | 6 | 7 | = напълно съгласен |
| --- | --- | --- | --- | --- | --- | --- | --- | --- |

| Връзката с облаги е добро нещо, защото може да помогне на бебчето/момчето да има задоволително финансово състояние. | \| 1 \| 2 \| 3 \| 4 \| 5 \| 6 \| 7 \| \| --- \| --- \| --- \| --- \| --- \| --- \| --- \| |
| --- | --- | --- | --- | --- | --- | --- | --- | --- |
| В бъдеще бих могъл да вляза във връзка с облаги. | \| 1 \| 2 \| 3 \| 4 \| 5 \| 6 \| 7 \| \| --- \| --- \| --- \| --- \| --- \| --- \| --- \| |
| Ако знаех, че няма да получа негативни оценки или няма да има негативни последици, бих искал да опитам връзка с облаги. | \| 1 \| 2 \| 3 \| 4 \| 5 \| 6 \| 7 \| \| --- \| --- \| --- \| --- \| --- \| --- \| --- \| |
| Ако това би било от полза за кариерата ми, бих обмислил да се впусна във връзка с облаги. | \| 1 \| 2 \| 3 \| 4 \| 5 \| 6 \| 7 \| \| --- \| --- \| --- \| --- \| --- \| --- \| --- \| |
| Бих обмислил сериозно да вляза във връзка с облаги, ако мисля, че това ще ми помогне да имам по-добро финансово състояние. | \| 1 \| 2 \| 3 \| 4 \| 5 \| 6 \| 7 \| \| --- \| --- \| --- \| --- \| --- \| --- \| --- \| |

Следните твърдения може да изглеждат подобни. Но Ви молим да оцените следните твърдения **сякаш сте татенце/мамче във връзка с облаги**, т.е. човек, който предоставя материални ресурси в замяна на компанията на партньора си. Моля, посечете степента, в която се съгласявате с всяко от твърденията по-долу като използвате 7-степенните рейтингови скали, вариращи от (1) “напълно несъгласен” до (7) “абсолютно съгласен”.

| напълно несъгласен = | 1 | 2 | 3 | 4 | 5 | 6 | 7 | = напълно съгласен |
| --- | --- | --- | --- | --- | --- | --- | --- | --- |

| Връзката с облаги е нещо добро, защото може да помогне на хората да се чувстват по-добре. | \| 1 \| 2 \| 3 \| 4 \| 5 \| 6 \| 7 \| \| --- \| --- \| --- \| --- \| --- \| --- \| --- \| |
| --- | --- | --- | --- | --- | --- | --- | --- | --- |
| В бъдеще бих могъл да вляза във връзка с облаги. | \| 1 \| 2 \| 3 \| 4 \| 5 \| 6 \| 7 \| \| --- \| --- \| --- \| --- \| --- \| --- \| --- \| |
| Ако знаех, че няма да получа негативни оценки или няма да има негативни последици, бих искал да опитам връзка с облаги. | \| 1 \| 2 \| 3 \| 4 \| 5 \| 6 \| 7 \| \| --- \| --- \| --- \| --- \| --- \| --- \| --- \| |
| Ако това би било от полза за сексуалния ми живот или за преценката на другите за мен, бих обмислил да вляза във връзка с облаги. | \| 1 \| 2 \| 3 \| 4 \| 5 \| 6 \| 7 \| \| --- \| --- \| --- \| --- \| --- \| --- \| --- \| |
| Сериозно бих обмислил да вляза във връзка с облаги, ако това беше начинът да намеря партньор, който да отговаря на всичките ми нужди. | \| 1 \| 2 \| 3 \| 4 \| 5 \| 6 \| 7 \| \| --- \| --- \| --- \| --- \| --- \| --- \| --- \| |

Language: Chinese (traditional)

**Country: China, Taiwan**

包養關係是指年長且資源較豐富的人（俗稱乾爹、乾媽）提供物質上的資源給年輕的伴侶以換取陪伴，是性交易的一種。雙方會在閒暇時間聚在一起，但是性行為是雙方同意才能發生。 但現在，請想像您是一個接受他人包養的年輕伴侶。 請你用以下的七分量表來評分你對下面各個敘述的同意程度，1分代表非常不同意、7分代表非常同意。.

| 非常不同意 = | 1 | 2 | 3 | 4 | 5 | 6 | 7 | = 非常同意 |
| --- | --- | --- | --- | --- | --- | --- | --- | --- |

| 包養關係是好的，因為可以緩解年輕伴侶的經濟負擔。 | \| 1 \| 2 \| 3 \| 4 \| 5 \| 6 \| 7 \| \| --- \| --- \| --- \| --- \| --- \| --- \| --- \| |
| --- | --- | --- | --- | --- | --- | --- | --- | --- |
| 我在未來有可能被人包養。 | \| 1 \| 2 \| 3 \| 4 \| 5 \| 6 \| 7 \| \| --- \| --- \| --- \| --- \| --- \| --- \| --- \| |
| 假如被包養不會帶來什麼負面名聲或者不好的結果，我也許會嘗試進入一段包養的關係 | \| 1 \| 2 \| 3 \| 4 \| 5 \| 6 \| 7 \| \| --- \| --- \| --- \| --- \| --- \| --- \| --- \| |
| 假如被包養會對我的未來生涯有正面幫助，我也許會嘗試進入一段包養的關係 | \| 1 \| 2 \| 3 \| 4 \| 5 \| 6 \| 7 \| \| --- \| --- \| --- \| --- \| --- \| --- \| --- \| |
| 假如被包養意謂著我有更多經濟資源，我會認真考慮是否進入一段包養的關係 | \| 1 \| 2 \| 3 \| 4 \| 5 \| 6 \| 7 \| \| --- \| --- \| --- \| --- \| --- \| --- \| --- \| |

下面這些敘述可能您才剛讀過一次。但現在，請重新想像您是一個包養其他人的乾爹或乾媽。 所謂的包養，是指提供物質資源以換取他人的陪伴。 請你用以下的七分量表來評分你對下面各個敘述的同意程度，1分代表非常不同意、7分代表非常同意

| 非常不同意 = | 1 | 2 | 3 | 4 | 5 | 6 | 7 | = 非常同意 |
| --- | --- | --- | --- | --- | --- | --- | --- | --- |

| 包養關係是好的，因為他讓包養者感覺更舒服。 | \| 1 \| 2 \| 3 \| 4 \| 5 \| 6 \| 7 \| \| --- \| --- \| --- \| --- \| --- \| --- \| --- \| |
| --- | --- | --- | --- | --- | --- | --- | --- | --- |
| 我在未來有可能包養其他人。 | \| 1 \| 2 \| 3 \| 4 \| 5 \| 6 \| 7 \| \| --- \| --- \| --- \| --- \| --- \| --- \| --- \| |
| 假如包養不會帶來什麼負面名聲或者不好的結果，我也許會嘗試進入一段包養的關係 | \| 1 \| 2 \| 3 \| 4 \| 5 \| 6 \| 7 \| \| --- \| --- \| --- \| --- \| --- \| --- \| --- \| |
| 假如包養會對我的性生活或對我的名聲有正面幫助，我也許會嘗試進入一段包養的關係 | \| 1 \| 2 \| 3 \| 4 \| 5 \| 6 \| 7 \| \| --- \| --- \| --- \| --- \| --- \| --- \| --- \| |
| 假如包養意謂著我可以找到一位全方位配合我的伴侶，我會認真考慮是否進入一段包養的關係 | \| 1 \| 2 \| 3 \| 4 \| 5 \| 6 \| 7 \| \| --- \| --- \| --- \| --- \| --- \| --- \| --- \| |

Language: Croatian

**Country: Croatia, Montenegro**

Veza  **sa sponzor(k)om ili sponzorska veza (eng. sugar relationship)** je transakciona seksualna veza u kojoj stariji/a i bogatiji/a partner tj. sponzor ili partnerka - sponzorka (eng. sugar daddy/mommy) pruža materijalna sredstva mlađem partneru – sponzorašu ili sponzoruši (eng. sugar baby/boy) kao nadoknadu za druženje. Partneri se obično sastaju kako bi proveli slobodno vreme zajedno, a seksualne aktivnosti su uključene samo ako oba partnera daju svoj pristanak.   Molimo ocenite sledeće izjave kao da ste **sugar baby/boy** ,tj. osoba koja prima materijalna sredstva za svoje druženje. Navedite u kojoj meri se slažete sa svakom od sledećih izjava koristeći skalu ocenjivanja od sedam nivoa u rasponu od (1) “apsolutno se ne slažem” do (7) “apsolutno se slažem”.

| uopšte se ne slažem = | 1 | 2 | 3 | 4 | 5 | 6 | 7 | = potpuno se slažem |
| --- | --- | --- | --- | --- | --- | --- | --- | --- |

| Sponzorska veza (engl. sugar relationship) je dobra stvar jer može pomoći partneru koji je s njim/njom u vezi da ima zadovoljavajuću finansijsku situaciju. | \| 1 \| 2 \| 3 \| 4 \| 5 \| 6 \| 7 \| \| --- \| --- \| --- \| --- \| --- \| --- \| --- \| |
| --- | --- | --- | --- | --- | --- | --- | --- | --- |
| U budućnosti bih mogao/la da budem u sponzorskoj vezi. | \| 1 \| 2 \| 3 \| 4 \| 5 \| 6 \| 7 \| \| --- \| --- \| --- \| --- \| --- \| --- \| --- \| |
| Kad bih znao/la da ne bi bilo negativnih osuda ili posledica, želeo/la bih da budem u sponzorskoj vezi. | \| 1 \| 2 \| 3 \| 4 \| 5 \| 6 \| 7 \| \| --- \| --- \| --- \| --- \| --- \| --- \| --- \| |
| Ako bi to koristilo mojoj karijeri, razmislio/la bih o tome da se upustim u sponzorsku vezu. | \| 1 \| 2 \| 3 \| 4 \| 5 \| 6 \| 7 \| \| --- \| --- \| --- \| --- \| --- \| --- \| --- \| |
| Ozbiljno bih razmotrio/la mogućnost sponzorske veze ako bih mislio/la da će to pomoći poboljšanju moje finansijske situacije. | \| 1 \| 2 \| 3 \| 4 \| 5 \| 6 \| 7 \| \| --- \| --- \| --- \| --- \| --- \| --- \| --- \| |

Sledeće izjave mogu izgledati slično. Ali sada, molimo Vas da ocenite sledeće izjave kao da **ste sponzor ili sponzorka (engl. sugar daddy/mommy)**, tj. osoba koja pruža materijalna sredstva svom partneru/ki u zamenu za ’druženje. Navedite u kojoj se meri slažete sa svakom od sledećih izjava koristeći sedmostepene skale u rasponu od (1) “apsolutno se ne slažem” do (7) “apsolutno se slažem”.

| uopšte se ne slažem = | 1 | 2 | 3 | 4 | 5 | 6 | 7 | = potpuno se slažem |
| --- | --- | --- | --- | --- | --- | --- | --- | --- |

| Sponzorska veza je dobra stvar jer može pomoći da se ljudi bolje osećaju. | \| 1 \| 2 \| 3 \| 4 \| 5 \| 6 \| 7 \| \| --- \| --- \| --- \| --- \| --- \| --- \| --- \| |
| --- | --- | --- | --- | --- | --- | --- | --- | --- |
| U budućnosti bih mogao/la da budem u sponzorskoj vezi. | \| 1 \| 2 \| 3 \| 4 \| 5 \| 6 \| 7 \| \| --- \| --- \| --- \| --- \| --- \| --- \| --- \| |
| Kad bih znao/la da ne bi bilo negativnih osuda ili posledica, želeo/la bih da se upustim u sponzorsku vezu. | \| 1 \| 2 \| 3 \| 4 \| 5 \| 6 \| 7 \| \| --- \| --- \| --- \| --- \| --- \| --- \| --- \| |
| Ukoliko bi sponzorska veza bila korisna za moj seksualni život, ili ako bi doprinela boljem mišljenju drugih o meni, ozbiljno bih razmislio/la da se upustim u takvu vezu. | \| 1 \| 2 \| 3 \| 4 \| 5 \| 6 \| 7 \| \| --- \| --- \| --- \| --- \| --- \| --- \| --- \| |
| Ozbiljno bih razmotrio/la mogućnost sponzorske veze ako bih mislio/la da će mi to pomoći da pronađem partnera/u koji/ja bi zadovoljavao/la sve moje potrebe. | \| 1 \| 2 \| 3 \| 4 \| 5 \| 6 \| 7 \| \| --- \| --- \| --- \| --- \| --- \| --- \| --- \| |

Language: Czech

**Country: Czech**

**Vztah se sugar daddy/mommy** je transakční sexuální vztah, ve kterém starší a zámožnější partner/ka (sugar daddy/mommy) poskytuje materiální zdroje mladší/mu partnerovi/ partnerce (sugar baby/boy) výměnou za jeho/její společnost. Partneři se obvykle scházejí, aby spolu trávili společně čas a sexuální aktivity jsou prováděny pouze, když s nimi oba partneři souhlasí.   Ohodnoťte, prosím, následující výroky, jako **kdybyste Vy byl/a sugar baby/boy**, tzn., člověk, který dostává za svou společnost materiální zdroje. Vyjádřete svůj souhlas na sedmibodové stupnici od (1) “zcela nesouhlasím” do (7) “zcela souhlasím”.

| zcela nesouhlasím = | 1 | 2 | 3 | 4 | 5 | 6 | 7 | = zcela souhlasím |
| --- | --- | --- | --- | --- | --- | --- | --- | --- |

| Vztah se sugar daddy/mommy je dobrá věc, protože může pomoci sugar baby/boy mít uspokojivou finanční situaci. | \| 1 \| 2 \| 3 \| 4 \| 5 \| 6 \| 7 \| \| --- \| --- \| --- \| --- \| --- \| --- \| --- \| |
| --- | --- | --- | --- | --- | --- | --- | --- | --- |
| V budoucnu bych mohl/a skončit ve vztahu se sugar daddy/mommy. | \| 1 \| 2 \| 3 \| 4 \| 5 \| 6 \| 7 \| \| --- \| --- \| --- \| --- \| --- \| --- \| --- \| |
| Kdybych věděl/a, že to pro mě nebude mít negativní dopady a že to nevyvolá odsouzení ostatních, rád/a bych vztah se sugar daddy/mommy vyzkoušel/a. | \| 1 \| 2 \| 3 \| 4 \| 5 \| 6 \| 7 \| \| --- \| --- \| --- \| --- \| --- \| --- \| --- \| |
| Přemýšlel bych o zapojení se do vztahu se sugar daddy/mommy, pokud by to prospělo mé kariéře. | \| 1 \| 2 \| 3 \| 4 \| 5 \| 6 \| 7 \| \| --- \| --- \| --- \| --- \| --- \| --- \| --- \| |
| Vážně bych zvažoval zapojení se do vztahu se sugar daddy/mommy, pokud bych si myslel, že mi to pomůže zlepšit finanční situaci. | \| 1 \| 2 \| 3 \| 4 \| 5 \| 6 \| 7 \| \| --- \| --- \| --- \| --- \| --- \| --- \| --- \| |

Následující výroky mohou vypadat podobně. Nyní ale, prosím, ohodnoťte výroky, jako kdybyste **byl/a tzv. sugar daddy/mommy**, tzn. člověk, který poskytuje materiální zdroje své/svému partnerovi/partnerce’ výměnou za jeho/její společnost. Uveďte, prosím, do jaké míry souhlasíte s následujícími výroky pomocí sedmibodové hodnotící stupnice, kde (1) znamená “zcela nesouhlasím” a (7) “zcela souhlasím”.

| zcela nesouhlasím = | 1 | 2 | 3 | 4 | 5 | 6 | 7 | = zcela souhlasím |
| --- | --- | --- | --- | --- | --- | --- | --- | --- |

| Vztah se sugar baby/boy je dobrá věc, protože může pomoci lidem cítit se lépe. | \| 1 \| 2 \| 3 \| 4 \| 5 \| 6 \| 7 \| \| --- \| --- \| --- \| --- \| --- \| --- \| --- \| |
| --- | --- | --- | --- | --- | --- | --- | --- | --- |
| V budoucnu bych mohl/a skončit ve vztahu se sugar baby/boy. | \| 1 \| 2 \| 3 \| 4 \| 5 \| 6 \| 7 \| \| --- \| --- \| --- \| --- \| --- \| --- \| --- \| |
| Kdybych věděl/a, že to pro mě nebude mít negativní dopady a že to nevyvolá odsouzení ostatních, rád/a bych vztah se sugar baby/boy vyzkoušel/a. | \| 1 \| 2 \| 3 \| 4 \| 5 \| 6 \| 7 \| \| --- \| --- \| --- \| --- \| --- \| --- \| --- \| |
| Pokud by to bylo prospěšné pro můj sexuální život nebo by to zvýšilo úsudek ostatních o mě, uvažoval/a bych o zapojení se do vztahu se sugar baby/boy. | \| 1 \| 2 \| 3 \| 4 \| 5 \| 6 \| 7 \| \| --- \| --- \| --- \| --- \| --- \| --- \| --- \| |
| Vážně bych zvažoval/a zapojení se do vztahu se sugar baby/boy, pokud by to byl způsob, jak najít partnera/partnerku, který/která splňuje všechny mé požadavky. | \| 1 \| 2 \| 3 \| 4 \| 5 \| 6 \| 7 \| \| --- \| --- \| --- \| --- \| --- \| --- \| --- \| |

Language: Dutch

**Country: Netherlands**

Een **suikerrelatie** is een transactionele seksuele relatie waarin een oudere en rijkere partner (suikeroom / mama) materiële middelen verstrekt aan een jongere partner (suikerbaby / jongen) in ruil voor haar of zijn gezelschap. Partners komen meestal samen om samen vrije tijd door te brengen, en seksuele activiteit is alleen mogelijk als beide partners hun toestemming geven. Beoordeel de volgende uitspraken alsof **je een suikerbaby/jongen bent**, d.w.z. een persoon die materiële middelen ontvangt voor zijn / haar gezelschap. Geef aan in hoeverre je het eens bent met elk van de onderstaande uitspraken met behulp van de beoordelingsschalen van zeven punten, variërend van (1) " “helemaal niet mee eens” tot (7) “helemaal mee eens”.

| absoluut oneens = | 1 | 2 | 3 | 4 | 5 | 6 | 7 | = absoluut eens |
| --- | --- | --- | --- | --- | --- | --- | --- | --- |

| Een suikerrelatie is een goede zaak omdat het de suikerbaby/-jongen kan helpen om een ​​bevredigende financiële situatie te hebben. | \| 1 \| 2 \| 3 \| 4 \| 5 \| 6 \| 7 \| \| --- \| --- \| --- \| --- \| --- \| --- \| --- \| |
| --- | --- | --- | --- | --- | --- | --- | --- | --- |
| In de toekomst zou ik een suikerrelatie kunnen aangaan. | \| 1 \| 2 \| 3 \| 4 \| 5 \| 6 \| 7 \| \| --- \| --- \| --- \| --- \| --- \| --- \| --- \| |
| Als ik wist dat ik geen negatief oordeel of consequenties zou krijgen, zou ik graag een suikerrelatie proberen. | \| 1 \| 2 \| 3 \| 4 \| 5 \| 6 \| 7 \| \| --- \| --- \| --- \| --- \| --- \| --- \| --- \| |
| Als het mijn carrière ten goede zou komen, zou ik erover nadenken om een ​​suikerrelatie aan te gaan. | \| 1 \| 2 \| 3 \| 4 \| 5 \| 6 \| 7 \| \| --- \| --- \| --- \| --- \| --- \| --- \| --- \| |
| Ik zou serieus overwegen om een ​​suikerrelatie aan te gaan als ik dacht dat het me zou helpen om een ​​betere financiële situatie te krijgen. | \| 1 \| 2 \| 3 \| 4 \| 5 \| 6 \| 7 \| \| --- \| --- \| --- \| --- \| --- \| --- \| --- \| |

De volgende uitspraken lijken misschien op elkaar. Maar beoordeel nu alstublieft de volgende uitspraken alsof u een sugar daddy / mama bent, d.w.z. een persoon die materiële middelen verschaft voor het gezelschap van haar / zijn partner. Geef aan in hoeverre u het eens bent met elk van de onderstaande uitspraken met behulp van de beoordelingsschalen van zeven punten, variërend van (1) "helemaal niet mee eens" tot (7) "helemaal mee eens".

| absoluut oneens = | 1 | 2 | 3 | 4 | 5 | 6 | 7 | = absoluut eens |
| --- | --- | --- | --- | --- | --- | --- | --- | --- |

| Een suikerrelatie is een goede zaak, omdat het mensen kan helpen zich beter te voelen. | \| 1 \| 2 \| 3 \| 4 \| 5 \| 6 \| 7 \| \| --- \| --- \| --- \| --- \| --- \| --- \| --- \| |
| --- | --- | --- | --- | --- | --- | --- | --- | --- |
| In de toekomst zou ik een suikerrelatie kunnen aangaan. | \| 1 \| 2 \| 3 \| 4 \| 5 \| 6 \| 7 \| \| --- \| --- \| --- \| --- \| --- \| --- \| --- \| |
| Als ik wist dat ik geen negatief oordeel of consequenties zou krijgen, zou ik graag een suikerrelatie proberen. | \| 1 \| 2 \| 3 \| 4 \| 5 \| 6 \| 7 \| \| --- \| --- \| --- \| --- \| --- \| --- \| --- \| |
| Als het gunstig zou zijn voor mijn seksleven of voor het oordeel van anderen over mij, zou ik overwegen om een ​​suikerrelatie aan te gaan. | \| 1 \| 2 \| 3 \| 4 \| 5 \| 6 \| 7 \| \| --- \| --- \| --- \| --- \| --- \| --- \| --- \| |
| Ik zou serieus overwegen om een ​​suikerrelatie aan te gaan als dat de manier was om een ​​partner te vinden die aan al mijn behoeften zou voldoen. | \| 1 \| 2 \| 3 \| 4 \| 5 \| 6 \| 7 \| \| --- \| --- \| --- \| --- \| --- \| --- \| --- \| |

Language: English

**Country: Bangladesh, Jamaica, United States,**

A **sugar relationship** is a transactional sexual relationship in which an older and wealthier partner (sugar daddy/mommy) provides material resources to a younger partner (sugar baby/boy) in return for her or his companionship. Partners usually meet to spend leisure time together, and sexual activity is only involved if both partners give their consent. Please rate the following statements as if **you were a sugar baby/boy**, i.e., a person who receives material resources for her/his companionship. Please indicate the extent to which you agree with each of the below statements using the seven-point rating scales ranging from (1) “absolutely disagree” to (7) “absolutely agree”.

| absolutely disagree = | 1 | 2 | 3 | 4 | 5 | 6 | 7 | = absolutely agree |
| --- | --- | --- | --- | --- | --- | --- | --- | --- |

| A sugar relationship is a good thing because it can help the sugar baby/boy have a satisfactory financial situation. | \| 1 \| 2 \| 3 \| 4 \| 5 \| 6 \| 7 \| \| --- \| --- \| --- \| --- \| --- \| --- \| --- \| |
| --- | --- | --- | --- | --- | --- | --- | --- | --- |
| In the future, I could end up engaging in a sugar relationship. | \| 1 \| 2 \| 3 \| 4 \| 5 \| 6 \| 7 \| \| --- \| --- \| --- \| --- \| --- \| --- \| --- \| |
| If I knew I would not incur negative judgment or consequences, I would like to try a sugar relationship. | \| 1 \| 2 \| 3 \| 4 \| 5 \| 6 \| 7 \| \| --- \| --- \| --- \| --- \| --- \| --- \| --- \| |
| If it would benefit my career, I would think about engaging in a sugar relationship. | \| 1 \| 2 \| 3 \| 4 \| 5 \| 6 \| 7 \| \| --- \| --- \| --- \| --- \| --- \| --- \| --- \| |
| I would seriously consider engaging in a sugar relationship if I thought it would help me have a better financial situation. | \| 1 \| 2 \| 3 \| 4 \| 5 \| 6 \| 7 \| \| --- \| --- \| --- \| --- \| --- \| --- \| --- \| |

The following statements might seem similar. But now, please rate the following statements as if **you were a sugar daddy/mommy**, i.e., a person who provides material resources for her/his partner’s companionship. Please indicate the extent to which you agree with each of the below statements using the seven-point rating scales ranging from (1) “absolutely disagree” to (7) “absolutely agree”.

| absolutely disagree = | 1 | 2 | 3 | 4 | 5 | 6 | 7 | = absolutely agree |
| --- | --- | --- | --- | --- | --- | --- | --- | --- |

| A sugar relationship is a good thing because it can help people feel better. | \| 1 \| 2 \| 3 \| 4 \| 5 \| 6 \| 7 \| \| --- \| --- \| --- \| --- \| --- \| --- \| --- \| |
| --- | --- | --- | --- | --- | --- | --- | --- | --- |
| In the future, I could end up engaging in a sugar relationship. | \| 1 \| 2 \| 3 \| 4 \| 5 \| 6 \| 7 \| \| --- \| --- \| --- \| --- \| --- \| --- \| --- \| |
| If I knew I would not incur negative judgment or consequences, I would like to try a sugar relationship. | \| 1 \| 2 \| 3 \| 4 \| 5 \| 6 \| 7 \| \| --- \| --- \| --- \| --- \| --- \| --- \| --- \| |
| If it would be beneficial for my sex life or for others’ judgment of me, I would consider engaging in a sugar relationship. | \| 1 \| 2 \| 3 \| 4 \| 5 \| 6 \| 7 \| \| --- \| --- \| --- \| --- \| --- \| --- \| --- \| |
| I would seriously consider engaging in a sugar relationship if that was the way to find a partner who would meet all my needs. | \| 1 \| 2 \| 3 \| 4 \| 5 \| 6 \| 7 \| \| --- \| --- \| --- \| --- \| --- \| --- \| --- \| |

Language: Estonian

**Country: Estonia**

Sponsorsuhteks nimetatakse suhet vanema ja jõukama partneri (suhkruissi/suhkruemme) ning noorema partneri vahel (suhkrubeebi), kus vanem partner pakub nooremale raha või muid hüvesid vastutasuks koos ajaveetmise või meeldivate seksuaalsete suhete eest. Sellises suhtes kohtuvad partnerid tavaliselt selleks, et koos vaba aega veeta ning seksuaalselt käiakse läbi vaid juhul, kui mõlemad osapooled sellega nõus on. Palume Teil hinnata järgmiseid väiteid, kujutades ette, et Teie olete suhkrubeebi ehk see partner, kes saab materjaalseid hüvesid vastutasuks oma seltsi pakkumise eest vanemale partnerile. Palume Teil 7-palliskaalal (kus 1 - pole üldse nõus ja 7 - täiesti nõus) märkida, mil määral Te nõustute järgnevate väidetega.

| ei ole üldse nõus = | 1 | 2 | 3 | 4 | 5 | 6 | 7 | = täiesti nõus |
| --- | --- | --- | --- | --- | --- | --- | --- | --- |

| Sponsorsuhe on hea, kuna see tagab suhkrubeebile rahuldustpakkuva majandusliku olukorra. | \| 1 \| 2 \| 3 \| 4 \| 5 \| 6 \| 7 \| \| --- \| --- \| --- \| --- \| --- \| --- \| --- \| |
| --- | --- | --- | --- | --- | --- | --- | --- | --- |
| Tulevikus võiksin kaaluda sponsorsuhtesse astumist. | \| 1 \| 2 \| 3 \| 4 \| 5 \| 6 \| 7 \| \| --- \| --- \| --- \| --- \| --- \| --- \| --- \| |
| Kui ma saaksin kindel olla, et sponsorsuhtes olemine ei paneks teisi minust halvasti arvama ega tooks mulle kaasa ebameeldivaid tagajärgi, siis mulle meeldiks proovida, mis tunne on olla sellises suhtes. | \| 1 \| 2 \| 3 \| 4 \| 5 \| 6 \| 7 \| \| --- \| --- \| --- \| --- \| --- \| --- \| --- \| |
| Ma kaaluksin sponsorsuhtesse astumist juhul, kui see oleks minu karjäärile kasulik. | \| 1 \| 2 \| 3 \| 4 \| 5 \| 6 \| 7 \| \| --- \| --- \| --- \| --- \| --- \| --- \| --- \| |
| Kui sponsorsuhe aitaks mul saavutada paremat majanduslikku olukorda, siis ma kaaluksin tõsiselt sellisesse suhtesse astumist. | \| 1 \| 2 \| 3 \| 4 \| 5 \| 6 \| 7 \| \| --- \| --- \| --- \| --- \| --- \| --- \| --- \| |

Järgnevad väited võivad näida eelmistega sarnased, ent seekord palume Teil ette kujutada, et olete suhkruissi või suhkruemme ehk see inimene, kes vastutasuks seltsi eest pakub oma partnerile materjaalseid hüvesid. Palume Teil 7-palliskaalal (kus 1 - pole üldse nõus ja 7 - täiesti nõus) märkida, mil määral Te nõustute järgnevate väidetega.

| ei ole üldse nõus = | 1 | 2 | 3 | 4 | 5 | 6 | 7 | = täiesti nõus |
| --- | --- | --- | --- | --- | --- | --- | --- | --- |

| Sponsorsuhe on hea, kuna see võib aidata inimestel ennast paremini tunda. | \| 1 \| 2 \| 3 \| 4 \| 5 \| 6 \| 7 \| \| --- \| --- \| --- \| --- \| --- \| --- \| --- \| |
| --- | --- | --- | --- | --- | --- | --- | --- | --- |
| Tulevikus võiksin kaaluda sponsorsuhtesse astumist. | \| 1 \| 2 \| 3 \| 4 \| 5 \| 6 \| 7 \| \| --- \| --- \| --- \| --- \| --- \| --- \| --- \| |
| Kui ma saaksin kindel olla, et sponsorsuhtes olemine ei paneks teisi minust halvasti arvama ega tooks mulle kaasa ebameeldivaid tagajärgi, siis mulle meeldiks proovida, mis tunne on olla sellises suhtes. | \| 1 \| 2 \| 3 \| 4 \| 5 \| 6 \| 7 \| \| --- \| --- \| --- \| --- \| --- \| --- \| --- \| |
| Ma kaaluksin sponsorsuhtesse astumist, kui see parendaks minu seksuaalelu ning teiste arvamust minust. | \| 1 \| 2 \| 3 \| 4 \| 5 \| 6 \| 7 \| \| --- \| --- \| --- \| --- \| --- \| --- \| --- \| |
| Kui sponsorsuhe võimaldaks mul leida partneri, kes vastaks kõikidele minu vajadusetele, siis ma kaaluksin sellisesse suhtesse astumist. | \| 1 \| 2 \| 3 \| 4 \| 5 \| 6 \| 7 \| \| --- \| --- \| --- \| --- \| --- \| --- \| --- \| |

Language: Finnish

**Country: Finland**

**Sokerisuhde** on liiketoiminnallinen seksuaalinen suhde, jossa vanhempi ja varakkaampi kumppani (sugardaddy/sugarmommy) antaa nuoremmalle kumppanille (sugar baby/toyboy) materiaalisia varoja vastineeksi kumppanuudesta. Kumppanit tapaavat yleensä viettääkseen vapaa-aikaa yhdessä. Seksuaalinen toiminta liittyy suhteeseen vain jos molemmat kumppanit antavat suostumuksensa. Arvioi seuraavat väittämät ikään kuin **olisit sugar baby/toyboy**, l., henkilö joka saa aineellisia varoja vastineeksi kumppanuudestaan. Ilmaise kuinka samaa mieltä olet alla olevien väittämien kanssa käyttämällä seitsemän-asteisia arviointiasteikkoja (1:stä) “ehdottomasti eri mieltä” (7:ään) “ehdottomasti samaa mieltä”.

| ehdottomasti eri mieltä = | 1 | 2 | 3 | 4 | 5 | 6 | 7 | = ehdottomasti samaa mieltä |
| --- | --- | --- | --- | --- | --- | --- | --- | --- |

| Sokerisuhde on hyvä asia koska se voi auttaa sugar babya/toyboyta tyydyttävän taloudellisen tilanteen omaamisessa. | \| 1 \| 2 \| 3 \| 4 \| 5 \| 6 \| 7 \| \| --- \| --- \| --- \| --- \| --- \| --- \| --- \| |
| --- | --- | --- | --- | --- | --- | --- | --- | --- |
| Voisin tulevaisuudessa päätyä sokerisuhteeseen. | \| 1 \| 2 \| 3 \| 4 \| 5 \| 6 \| 7 \| \| --- \| --- \| --- \| --- \| --- \| --- \| --- \| |
| Jos tietäisin että en saattaisi itseäni alttiiksi kielteiselle arvostelulle tai seuraamuksille, haluaisin kokeilla sokerisuhdetta. | \| 1 \| 2 \| 3 \| 4 \| 5 \| 6 \| 7 \| \| --- \| --- \| --- \| --- \| --- \| --- \| --- \| |
| Harkitsisin sokerisuhdetta, jos se olisi hyödyksi uralleni. | \| 1 \| 2 \| 3 \| 4 \| 5 \| 6 \| 7 \| \| --- \| --- \| --- \| --- \| --- \| --- \| --- \| |
| Harkitsisin tosissani sokerisuhdetta, jos luulisin sen auttavan minua paremman taloudellisen tilanteen saamisessa. | \| 1 \| 2 \| 3 \| 4 \| 5 \| 6 \| 7 \| \| --- \| --- \| --- \| --- \| --- \| --- \| --- \| |

Seuraavat väittämät saattavat vaikuttaa samankaltaisilta. Mutta nyt, arvioi seuraavat väittämät ikään kuin **olisit sugardaddy/sugarmommyy**, l., henkilö joka antaa aineellisia varoja kumppannilleen vastineeksi hänen’ seurastaan. Ilmaise kuinka samaa mieltä olet alla olevien väittämien kanssa käyttäen seitsemän-asteisia arviointiasteikkoja (1:stä) “ehdottomasti eri mieltä” (7:ään) “ehdottomasti samaa mieltä”.

| ehdottomasti eri mieltä = | 1 | 2 | 3 | 4 | 5 | 6 | 7 | = ehdottomasti samaa mieltä |
| --- | --- | --- | --- | --- | --- | --- | --- | --- |

| Sokerisuhde on hyvä asia, koska se voi auttaa ihmisiä voimaan paremmin. | \| 1 \| 2 \| 3 \| 4 \| 5 \| 6 \| 7 \| \| --- \| --- \| --- \| --- \| --- \| --- \| --- \| |
| --- | --- | --- | --- | --- | --- | --- | --- | --- |
| Tulevaisuudessa voisin päätyä sokerisuhteeseen. | \| 1 \| 2 \| 3 \| 4 \| 5 \| 6 \| 7 \| \| --- \| --- \| --- \| --- \| --- \| --- \| --- \| |
| Haluaisin kokeilla sokerisuhdetta, jos tietäisin että en saattaisi itseäni alttiiksi kielteiselle arvostelulle tai seuraamuksille. | \| 1 \| 2 \| 3 \| 4 \| 5 \| 6 \| 7 \| \| --- \| --- \| --- \| --- \| --- \| --- \| --- \| |
| Harkitsisin sokerisuhdetta, jos se olisi hyödyksi seksielämälleni tai hyödyttäisi miten toiset arvioivat minut. | \| 1 \| 2 \| 3 \| 4 \| 5 \| 6 \| 7 \| \| --- \| --- \| --- \| --- \| --- \| --- \| --- \| |
| Harkitsisin tosissani sokerisuhdetta, jos sillä tavoin löytäisin kumppanin joka täyttäisi kaikki tarpeeni. | \| 1 \| 2 \| 3 \| 4 \| 5 \| 6 \| 7 \| \| --- \| --- \| --- \| --- \| --- \| --- \| --- \| |

Language: French

**Country: France, Belgium, Canada**

Une **sugar relationship** est une relation sexuelle transactionnelle dans laquelle un partenaire plus âgé et plus riche (sugar daddy/mommy) fournit des ressources matérielles à un partenaire plus jeune (sugar baby/boy) en échange de sa compagnie. Les partenaires se rencontrent généralement pour passer du temps libre ensemble, et l'activité sexuelle n'a lieu que si les deux partenaires donnent leur consentement. Veuillez évaluer les affirmations suivantes comme si vous étiez un **sugar baby/boy**, c'est-à-dire une personne qui reçoit des ressources matérielles en échange de sa compagnie. Veuillez indiquer dans quelle mesure vous êtes d'accord avec chacune des affirmations ci-dessous en utilisant les échelles d'évaluation en sept points allant de(1) “absolument pas d'accord” (7) “absolument d'accord”.

| absolument pas d’accord = | 1 | 2 | 3 | 4 | 5 | 6 | 7 | = absolument d'accord |
| --- | --- | --- | --- | --- | --- | --- | --- | --- |

| Une “sugar relationship” est une bonne chose car elle peut aider le sugar baby/boy à avoir une situation financière satisfaisante. | \| 1 \| 2 \| 3 \| 4 \| 5 \| 6 \| 7 \| \| --- \| --- \| --- \| --- \| --- \| --- \| --- \| |
| --- | --- | --- | --- | --- | --- | --- | --- | --- |
| Dans le futur, je pourrais m'engager dans une "sugar relationship". | \| 1 \| 2 \| 3 \| 4 \| 5 \| 6 \| 7 \| \| --- \| --- \| --- \| --- \| --- \| --- \| --- \| |
| Si je savais que je ne subirais pas de jugement négatif ou de conséquences, j'aimerais essayer une “sugar relationship”. | \| 1 \| 2 \| 3 \| 4 \| 5 \| 6 \| 7 \| \| --- \| --- \| --- \| --- \| --- \| --- \| --- \| |
| Si cela pouvait être bénéfique pour ma carrière, j'envisagerais de m'engager dans une “sugar relationship”. | \| 1 \| 2 \| 3 \| 4 \| 5 \| 6 \| 7 \| \| --- \| --- \| --- \| --- \| --- \| --- \| --- \| |
| J'envisagerais sérieusement de m'engager dans une “sugar relationship” si je pensais que cela m'aiderait à avoir une meilleure situation financière. | \| 1 \| 2 \| 3 \| 4 \| 5 \| 6 \| 7 \| \| --- \| --- \| --- \| --- \| --- \| --- \| --- \| |

Les propositions suivantes peuvent sembler similaires aux précédentes. Mais maintenant, veuillez s’il vous plaît évaluer les propositions suivantes comme si **vous étiez un sugar daddy/ sugar mommy**, i.e., une personne qui donne des ressources matérielles à son/sa partenaire’ en échange de sa compagnie. Dans quelle mesure êtes-vous d'accord avec les affirmations suivantes, en utilisant une échelle de 7 points allant de (1) “absolument pas d’accord” à (7) “absolument d'accord”.

| absolument pas d’accord = | 1 | 2 | 3 | 4 | 5 | 6 | 7 | = absolument d'accord |
| --- | --- | --- | --- | --- | --- | --- | --- | --- |

| Une relation de type "sugar daddy / sugar mommy" est une bonne chose car elle peut aider les gens à se sentir mieux. | \| 1 \| 2 \| 3 \| 4 \| 5 \| 6 \| 7 \| \| --- \| --- \| --- \| --- \| --- \| --- \| --- \| |
| --- | --- | --- | --- | --- | --- | --- | --- | --- |
| Dans le futur, je pourrais m'engager dans une "sugar relationship". | \| 1 \| 2 \| 3 \| 4 \| 5 \| 6 \| 7 \| \| --- \| --- \| --- \| --- \| --- \| --- \| --- \| |
| Si je savais que je ne subirais pas de jugement négatif ou de conséquences néfastes, j'aimerais essayer une relation de type "sugar daddy / sugar mommy". | \| 1 \| 2 \| 3 \| 4 \| 5 \| 6 \| 7 \| \| --- \| --- \| --- \| --- \| --- \| --- \| --- \| |
| Si cela pouvait être bénéfique pour ma vie sexuelle ou pour le jugement des autres sur moi, je pourrais m'engager dans une relation de type "sugar daddy / sugar mommy". | \| 1 \| 2 \| 3 \| 4 \| 5 \| 6 \| 7 \| \| --- \| --- \| --- \| --- \| --- \| --- \| --- \| |
| J'envisagerais sérieusement de m'engager dans une relation de type "sugar daddy / sugar mommy" si c’était le moyen de trouver un(e) partenaire qui répondrait à tous mes besoins. | \| 1 \| 2 \| 3 \| 4 \| 5 \| 6 \| 7 \| \| --- \| --- \| --- \| --- \| --- \| --- \| --- \| |

Language: Georgian

**Country: Georgia**

**ანგარებიანი ურთიერთობა** არის გარიგებითი სქესობრივი კავშირი, რომელშიც უფროსი და მდიდარი პარტნიორი („ტკბილი“ მამა / დედა) მატერიალურად უზრუნველყოფს უმცროს პარტნიორს („ტკბილ ბავშვს" / "ტკბილ ბიჭს") მისი ან მისი მეგობრობის სანაცვლოდ. ჩვეულებრივ, პარტნიორები ერთმანეთს ხვდებიან დროის ერთად გასატარებლად და სექსუალური ურთიერთობა დასაშვებია მხოლოდ იმ შემთხვევაში, თუ ორივე პარტნიორს აქვს ამის სურვილი. გთხოვთ, შეაფასოთ ქვემოთ ჩამოთვლილი გამონათქვამები**"ტკბილი ბავშვის" პოზიციიდან**, მაგ., პირი, რომელიც იღებს მატერიალურ სარგებელს თავისი კომპანიონობისთვის. გთხოვთ, მიუთითოთ, თუ რამდენად ეთანხმებით თითოეულ ქვემოთ მოცემულ დებულებას შვიდ საფეხურიან სკალაზე, სადაც (1) ნიშნავს "აბსოლუტურად არ ვეთანხმები", ხოლო (7) "აბსოლუტურად ვეთანხმები".

| აბსოლუტურად არ ვეთანხმები = | 1 | 2 | 3 | 4 | 5 | 6 | 7 | = აბსოლუტურად ვეთანხმები |
| --- | --- | --- | --- | --- | --- | --- | --- | --- |

| ანგარებიანი ურთიერთობა კარგია, რადგან მას შეუძლია დაეხმაროს ბავშვს / ბიჭს დამაკმაყოფილებელი ფინანსური მდგომარეობა ჰქონდეს. | \| 1 \| 2 \| 3 \| 4 \| 5 \| 6 \| 7 \| \| --- \| --- \| --- \| --- \| --- \| --- \| --- \| |
| --- | --- | --- | --- | --- | --- | --- | --- | --- |
| მომავალში შეიძლება ანგარებიანი ურთიერთობით დავასრულო (ჩემი ფინალი შეიძება იყოს ანგარებიანი ურთიერთობა) | \| 1 \| 2 \| 3 \| 4 \| 5 \| 6 \| 7 \| \| --- \| --- \| --- \| --- \| --- \| --- \| --- \| |
| რომ ვიცოდე, რომ ჩემს ქცევას არ მოჰყვება ნეგატიური შეფასება და შედეგები, ვცდიდი ანგარებიან ურთიერთობას. | \| 1 \| 2 \| 3 \| 4 \| 5 \| 6 \| 7 \| \| --- \| --- \| --- \| --- \| --- \| --- \| --- \| |
| თუ ეს სარგებელს მოუტანდა ჩემს კარიერას, ვიფიქრებდი ანგარებიან ურთიერთობაზე. | \| 1 \| 2 \| 3 \| 4 \| 5 \| 6 \| 7 \| \| --- \| --- \| --- \| --- \| --- \| --- \| --- \| |
| სერიოზულად განვიხილავდი ანგარებიან ურთიერთობაში ჩართვას, თუ ვიფიქრებდი, რომ ეს დამეხმარებოდა უკეთესი ფინანსური მდგომარეობის მიღებაში. | \| 1 \| 2 \| 3 \| 4 \| 5 \| 6 \| 7 \| \| --- \| --- \| --- \| --- \| --- \| --- \| --- \| |

ქვემოთმოცემული გამონათქვამები შეიძლება მოგეჩვენოთ წინა გამონათქვამების მსგავსი. თუმცა ახლა გთხოვთ შეაფასოთ შემდეგი დებულებები, თითქოს **„ტკბილი“/სპონსორი მამა / დედა ხართ**, ანუ ადამიანი, რომელიც მატერიალურად უზრუნველყოფს მის პარტნიორს მეგობრობისთვის. გთხოვთ, აღნიშნოთ, თუ რამდენად ეთანხმებით თითოეულ ქვემოთ მოცემულ დებულებას შვიდ ბალიან სკალაზე, სადაც (1) ნიშნავს "აბსოლუტურად არ ვეთანხმები", ხოლო (7) "აბსოლუტურად ვეთანხმები".

| აბსოლუტურად არ ვეთანხმები = | 1 | 2 | 3 | 4 | 5 | 6 | 7 | = აბსოლუტურად ვეთანხმები |
| --- | --- | --- | --- | --- | --- | --- | --- | --- |

| ანგარებიანი ურთიერთობა კარგია, რადგან ის შეიძლება დაეხმაროს ადამიანებს უკეთესად იგრძნონ თავი. | \| 1 \| 2 \| 3 \| 4 \| 5 \| 6 \| 7 \| \| --- \| --- \| --- \| --- \| --- \| --- \| --- \| |
| --- | --- | --- | --- | --- | --- | --- | --- | --- |
| მომავალში შეიძლება ანგარებიანი ურთიერთობით დავასრულო | \| 1 \| 2 \| 3 \| 4 \| 5 \| 6 \| 7 \| \| --- \| --- \| --- \| --- \| --- \| --- \| --- \| |
| რომ ვიცოდე, რომ ჩემს ქცევას არ მოჰყვება ნეგატიური შეფასება და შედეგები, ვცდიდი ანგარებიან ურთიერთობას. | \| 1 \| 2 \| 3 \| 4 \| 5 \| 6 \| 7 \| \| --- \| --- \| --- \| --- \| --- \| --- \| --- \| |
| თუ ეს სარგებელს მოუტანდა ჩემს კარიერას, ვიფიქრებდი ანგარებიან ურთიერთობაზე. | \| 1 \| 2 \| 3 \| 4 \| 5 \| 6 \| 7 \| \| --- \| --- \| --- \| --- \| --- \| --- \| --- \| |
| სერიოზულად განვიხილავდი ანგარებიან ურთიერთობაში ჩართვას, თუ ვიფიქრებდი, რომ ეს დამეხმარებოდა უკეთესი ფინანსური მდგომარეობის მიღებაში. | \| 1 \| 2 \| 3 \| 4 \| 5 \| 6 \| 7 \| \| --- \| --- \| --- \| --- \| --- \| --- \| --- \| |

Language: German

**Country: Austria, Germany, Switzerland**

Eine **"Sugar Beziehung"** ist eine sexuelle Beziehung, in der ein(e) ältere(r) und wohlhabendere(r) Partner / Partnerin (Sugar Daddy / Sugar Mommy) einem / einer jüngeren Partner /Partnerin materielle Ressourcen zur Verfügung stellt und im Gegenzug von dessen / deren Gesellschaft profitiert. Die Partner / Partnerinnen treffen sich normalerweise, um Freizeit zusammen zu verbringen. Sexuelle Aktivitäten sind nur involviert, wenn beide Parteien damit einverstanden sind. Bitte bewerten Sie die folgenden Aussagen, als ob Sie **ein Sugar Baby / Boy wären**, d.h. eine Person, die materielle Ressourcen erhält für die geleistete Gesellschaft. Bitte geben Sie in der Folge an, wie stark Sie jeder der folgenden Aussagen zustimmen, auf einer 7-Punkte-Skala von (1) “Stimme überhaupt nicht zu” bis (7) “Stimme voll zu”.

| stimme überhaupt nicht zu = | 1 | 2 | 3 | 4 | 5 | 6 | 7 | = stimme voll zu |
| --- | --- | --- | --- | --- | --- | --- | --- | --- |

| Eine "Sugar Beziehung" ist eine gute Sache, weil es dem Sugar Baby / Boy zu einer zufriedenstellenden finanziellen Situation verhelfen kann. | \| 1 \| 2 \| 3 \| 4 \| 5 \| 6 \| 7 \| \| --- \| --- \| --- \| --- \| --- \| --- \| --- \| |
| --- | --- | --- | --- | --- | --- | --- | --- | --- |
| Zukünftig könnte ich in einer solchen Beziehung enden. | \| 1 \| 2 \| 3 \| 4 \| 5 \| 6 \| 7 \| \| --- \| --- \| --- \| --- \| --- \| --- \| --- \| |
| Wenn ich wüsste, dass es keine negativen gesellschaftlichen Urteile oder Konsequenzen gäbe, würde ich gerne eine "Sugar Beziehung" probieren. | \| 1 \| 2 \| 3 \| 4 \| 5 \| 6 \| 7 \| \| --- \| --- \| --- \| --- \| --- \| --- \| --- \| |
| Wenn es meiner Karriere helfen würde, würde ich über eine "Sugar Beziehung" nachdenken. | \| 1 \| 2 \| 3 \| 4 \| 5 \| 6 \| 7 \| \| --- \| --- \| --- \| --- \| --- \| --- \| --- \| |
| Ich würde ernsthaft in Erwägung ziehen, eine "Sugar Beziehung" zu führen, wenn ich denken würde, dass es förderlich für meine finanzielle Situation wäre. | \| 1 \| 2 \| 3 \| 4 \| 5 \| 6 \| 7 \| \| --- \| --- \| --- \| --- \| --- \| --- \| --- \| |

Die folgenden Aussagen mögen ähnlich erscheinen. Aber jetzt bewerten Sie bitte die folgenden Aussagen so, als ob **Sie ein / eine Sugar Daddy / Mommy** wären, d.h. eine Person, die materielle Ressourcen im Austausch für die Gesellschaft ihres / seines Partners zur Verfügung stellt. Bitte geben Sie an, inwieweit Sie jeder der folgenden Aussagen zustimmen, indem Sie die siebenstufige Bewertungsskala von (1) “stimme überhaupt nicht zu” bis (7) “stimme voll zu” verwenden.

| stimme überhaupt nicht zu = | 1 | 2 | 3 | 4 | 5 | 6 | 7 | = stimme voll zu |
| --- | --- | --- | --- | --- | --- | --- | --- | --- |

| Eine solche "Sugar Beziehung" ist eine gute Sache, weil es Menschen helfen kann, sich besser zu fühlen. | \| 1 \| 2 \| 3 \| 4 \| 5 \| 6 \| 7 \| \| --- \| --- \| --- \| --- \| --- \| --- \| --- \| |
| --- | --- | --- | --- | --- | --- | --- | --- | --- |
| Zukünftig könnte ich in einer solchen Beziehung enden. | \| 1 \| 2 \| 3 \| 4 \| 5 \| 6 \| 7 \| \| --- \| --- \| --- \| --- \| --- \| --- \| --- \| |
| Wenn ich wüsste, dass es keine negativen gesellschaftlichen Urteile oder Konsequenzen gäbe, würde ich gerne eine "Sugar Beziehung" probieren. | \| 1 \| 2 \| 3 \| 4 \| 5 \| 6 \| 7 \| \| --- \| --- \| --- \| --- \| --- \| --- \| --- \| |
| Wenn es für mein Sexualleben oder für das Urteil Anderer über mich zuträglich wäre, würde ich eine «Sugar Beziehung» in Betracht ziehen.^*^ | \| 1 \| 2 \| 3 \| 4 \| 5 \| 6 \| 7 \| \| --- \| --- \| --- \| --- \| --- \| --- \| --- \| |
| Ich würde ernsthaft in Erwägung ziehen, eine "Sugar Beziehung" zu führen, wenn ich einen Partner / eine Partnerin fände, der/die alle meine Bedürfnisse erfüllt. | \| 1 \| 2 \| 3 \| 4 \| 5 \| 6 \| 7 \| \| --- \| --- \| --- \| --- \| --- \| --- \| --- \| |

* This item of the questionnaire package used in the research was inaccurately used in the following form: Es wäre meinem Sexleben oder dem Urteil anderer über mich zuträglich, wenn ich eine “Sugar Beziehung” in Betracht zöge.

Language: Greek

**Country: Greece**

Μια **«sugar σχέση»** είναι μια ερωτική σχέση συναλλαγής στην οποία ένας μεγαλύτερος και πλουσιότερος σύντροφος (sugar daddy/mommy) παρέχει υλικές απολαβές σε έναν νεότερο σύντροφο (sugar baby/boy) με αντάλλαγμα τη συντροφιά της/του. Οι σύντροφοι συνήθως συναντιούνται για να περάσουν μαζί τον ελεύθερο χρόνο τους και η σεξουαλική δραστηριότητα υπάρχει μόνο εφόσον και οι δύο σύντροφοι συναινέσουν. Παρακαλούμε να αξιολογήσετε τις ακόλουθες δηλώσεις **σαν να ήσασταν μία/ένας sugar baby/boy**, δηλαδή άτομο που λαμβάνει υλικούς πόρους για τη συντροφιά του. Παρακαλούμε να σημειώσετε τον βαθμό στον οποίο συμφωνείτε με καθεμία από τις παρακάτω δηλώσεις χρησιμοποιώντας την επταβάθμια κλίμακα που κυμαίνεται από το (1) «διαφωνώ απόλυτα» έως το (7) «συμφωνώ απόλυτα».

| Διαφωνώ απόλυτα = | 1 | 2 | 3 | 4 | 5 | 6 | 7 | = Συμφωνώ απόλυτα |
| --- | --- | --- | --- | --- | --- | --- | --- | --- |

| Μία «sugar σχέση» είναι κάτι καλό γιατί μπορεί να βοηθήσει την/τον sugar baby/boy να βρίσκεται σε καλή οικονομική κατάσταση. | \| 1 \| 2 \| 3 \| 4 \| 5 \| 6 \| 7 \| \| --- \| --- \| --- \| --- \| --- \| --- \| --- \| |
| --- | --- | --- | --- | --- | --- | --- | --- | --- |
| Στο μέλλον, θα μπορούσα τελικά να εμπλακώ σε μία «sugar σχέση». | \| 1 \| 2 \| 3 \| 4 \| 5 \| 6 \| 7 \| \| --- \| --- \| --- \| --- \| --- \| --- \| --- \| |
| Εάν γνώριζα ότι δεν θα δεχθώ αρνητική κριτική ή αρνητικές συνέπειες, θα ήθελα να δοκιμάσω μία «sugar σχέση». | \| 1 \| 2 \| 3 \| 4 \| 5 \| 6 \| 7 \| \| --- \| --- \| --- \| --- \| --- \| --- \| --- \| |
| Αν αυτό θα βοηθούσε την καριέρα μου, θα σκεφτόμουν να εμπλακώ σε μία «sugar σχέση». | \| 1 \| 2 \| 3 \| 4 \| 5 \| 6 \| 7 \| \| --- \| --- \| --- \| --- \| --- \| --- \| --- \| |
| Αν θεωρούσα ότι θα με βοηθούσε να βρεθώ σε καλύτερη οικονομική κατάσταση, θα σκεφτόμουν σοβαρά να εμπλακώ σε μια «sugar σχέση». | \| 1 \| 2 \| 3 \| 4 \| 5 \| 6 \| 7 \| \| --- \| --- \| --- \| --- \| --- \| --- \| --- \| |

Οι ακόλουθες δηλώσεις μπορεί να φαίνονται παρόμοιες. Αλλά τώρα, παρακαλούμε να αξιολογήσετε τις ακόλουθες δηλώσεις **σαν να ήσασταν ένας/μία sugar daddy/mommyy**, δηλαδή άτομο που παρέχει υλικούς πόρους για τη συντροφιά του/της συντρόφου του. Παρακαλούμε να σημειώσετε τον βαθμό στον οποίο συμφωνείτε με καθεμία από τις παρακάτω δηλώσεις χρησιμοποιώντας την επταβάθμια κλίμακα που κυμαίνεται από το (1) «διαφωνώ απόλυτα» έως το (7) «συμφωνώ απόλυτα».

| Διαφωνώ απόλυτα = | 1 | 2 | 3 | 4 | 5 | 6 | 7 | = Συμφωνώ απόλυτα |
| --- | --- | --- | --- | --- | --- | --- | --- | --- |

| Μία «sugar σχέση» είναι κάτι καλό γιατί μπορεί να βοηθήσει τα άτομα να αισθάνονται καλύτερα. | \| 1 \| 2 \| 3 \| 4 \| 5 \| 6 \| 7 \| \| --- \| --- \| --- \| --- \| --- \| --- \| --- \| |
| --- | --- | --- | --- | --- | --- | --- | --- | --- |
| Στο μέλλον, θα μπορούσα τελικά να εμπλακώ σε μία «sugar σχέση». | \| 1 \| 2 \| 3 \| 4 \| 5 \| 6 \| 7 \| \| --- \| --- \| --- \| --- \| --- \| --- \| --- \| |
| Εάν γνώριζα ότι δεν θα δεχθώ αρνητική κριτική ή αρνητικές συνέπειες, θα ήθελα να δοκιμάσω μία «sugar σχέση». | \| 1 \| 2 \| 3 \| 4 \| 5 \| 6 \| 7 \| \| --- \| --- \| --- \| --- \| --- \| --- \| --- \| |
| Αν θα ήταν ωφέλιμο για τη σεξουαλική μου ζωή ή για τη γνώμη των άλλων για μένα, θα σκεφτόμουν να εμπλακώ σε μία «sugar σχέση». | \| 1 \| 2 \| 3 \| 4 \| 5 \| 6 \| 7 \| \| --- \| --- \| --- \| --- \| --- \| --- \| --- \| |
| Θα σκεφτόμουν σοβαρά να εμπλακώ σε μια «sugar σχέση», αν αυτός ήταν ο τρόπος να βρω έναν σύντροφο που θα ικανοποιούσε όλες τις ανάγκες μου. | \| 1 \| 2 \| 3 \| 4 \| 5 \| 6 \| 7 \| \| --- \| --- \| --- \| --- \| --- \| --- \| --- \| |

Language: Hebrew

**Country: Israel**

**מערכת יחסים מסוג "שוגר" (Sugar relationship)** היא מערכת יחסים מינית בה פרטנר מבוגר ועשיר יותר ("שוגר דאדי/מאמי") מספק משאבים חומריים לפרטנר צעיר יותר ("שוגר בייבי"), בתמורה לחברתו. הפרטנרים בדרך כלל נפגשים כדי לבלות יחד בפעילויות פנאי, ופעילות מינית מתרחשת רק אם שני הפרטנרים מסכימים לכך.   לפניך מספר הצהרות. אנא השב עליהן כשאתה שוקל את האופציה של להיות **"שוגר בייבי"**, כלומר, אדם שמקבל משאבים חומריים בתמורה לחברתו. כעת, ציין עד כמה אתה מסכים עם כל אחת מההצהרות שלהלן, בשימוש בסולם 7-נקודות, הנע בין (1)“כלל לא מסכים” ל(7)“מסכים בהחלט”.

| מסכים בהחלט = | 7 | 6 | 5 | 4 | 3 | 2 | 1 | = כלל לא מסכים |
| --- | --- | --- | --- | --- | --- | --- | --- | --- |

| \| 7 \| 6 \| 5 \| 4 \| 3 \| 2 \| 1 \| \| --- \| --- \| --- \| --- \| --- \| --- \| --- \| | מערכת יחסים מסוג "שוגר" היא דבר טוב, כיוון שהיא עוזרת ל"שוגר בייבי" להיות במצב כלכלי מספק. |
| --- | --- | --- | --- | --- | --- | --- | --- | --- |
| \| 7 \| 6 \| 5 \| 4 \| 3 \| 2 \| 1 \| \| --- \| --- \| --- \| --- \| --- \| --- \| --- \| | בעתיד, אוכל לקיים מערכת יחסים מסוג "שוגר". |
| \| 7 \| 6 \| 5 \| 4 \| 3 \| 2 \| 1 \| \| --- \| --- \| --- \| --- \| --- \| --- \| --- \| | אם הייתי יודע שלא יהיה שיפוט שלילי או השלכות, הייתי רוצה לנסות מערכת יחסים מסוג "שוגר". |
| \| 7 \| 6 \| 5 \| 4 \| 3 \| 2 \| 1 \| \| --- \| --- \| --- \| --- \| --- \| --- \| --- \| | אם זה היה מועיל לקריירה שלי, הייתי חושב על לקיים מערכת יחסים מסוג "שוגר". |
| \| 7 \| 6 \| 5 \| 4 \| 3 \| 2 \| 1 \| \| --- \| --- \| --- \| --- \| --- \| --- \| --- \| | הייתי שוקל ברצינות לקיים מערכת יחסים מסוג "שוגר" אם הייתי חושב שזה יעזור לי להיות במצב כלכלי טוב יותר. |

ההצהרות הבאות עשויות להיראות דומות. אבל עכשיו, אנא השב עליהן כשאתה שוקל את האופציה של להיות  **"שוגר דאדי/מאמי"**,כלומר, אדם שמספק משאבים חומריים בתמורה לחברת הפרטנר שלו. כעת, ציין עד כמה אתה מסכים עם כל אחת מההצהרות שלהלן, בשימוש בסולם 7-נקודות, הנע בין (1)“כלל לא מסכים” ל(7)“מסכים בהחלט”.

| מסכים בהחלט = | 7 | 6 | 5 | 4 | 3 | 2 | 1 | = כלל לא מסכים |
| --- | --- | --- | --- | --- | --- | --- | --- | --- |

| \| 7 \| 6 \| 5 \| 4 \| 3 \| 2 \| 1 \| \| --- \| --- \| --- \| --- \| --- \| --- \| --- \| | מערכת יחסים מסוג "שוגר" היא דבר טוב, כיוון שהיא יכולה לעזור לאנשים להרגיש טוב יותר. |
| --- | --- | --- | --- | --- | --- | --- | --- | --- |
| \| 7 \| 6 \| 5 \| 4 \| 3 \| 2 \| 1 \| \| --- \| --- \| --- \| --- \| --- \| --- \| --- \| | בעתיד, אוכל לקיים מערכת יחסים מסוג "שוגר" |
| \| 7 \| 6 \| 5 \| 4 \| 3 \| 2 \| 1 \| \| --- \| --- \| --- \| --- \| --- \| --- \| --- \| | אם הייתי יודע שלא יהיה שיפוט שלילי או השלכות, הייתי רוצה לנסות מערכת יחסים מסוג "שוגר". |
| \| 7 \| 6 \| 5 \| 4 \| 3 \| 2 \| 1 \| \| --- \| --- \| --- \| --- \| --- \| --- \| --- \| | אם זה היה מועיל לחיי המין שלי או לאופן בו אחרים יעריכו אותי, הייתי חשוב על לקיים מערכת יחסים מסוג "שוגר". |
| \| 7 \| 6 \| 5 \| 4 \| 3 \| 2 \| 1 \| \| --- \| --- \| --- \| --- \| --- \| --- \| --- \| | הייתי שוקל ברצינות לקיים מערכת יחסים מסוג "שוגר" אם זו הייתה הדרך למצוא פרטנר שיענה על כל צרכיי. |

Language: Hungarian

**Country: Hungary**

A **sugar kapcsolat** olyan üzleti alapú viszony, amelyben az idősebb és tehetősebb fél (sugar daddy, ha férfi, illetve sugar mommy, ha nő) anyagi támogatást nyújt a fiatalabb félnek (sugar baby, ha nő, illetve sugar boy, ha férfi) a találkozásokért cserébe. A találkozások általában szabadidős tevékenységek, amelyek akkor szexuális természetűek, ha mindkét fél beleegyezik ebbe. Kérjük, értékelje a következő állításokat úgy, mintha **sugar baby/boy lenne**, azaz egy olyan személy, aki anyagi forrásokat kap társaságáért cserébe. Kérjük, jelölje az alábbi állítások után, hogy mennyire ért egyet velük egy hétfokú skála segítségével, ahol (1) “egyáltalán nem értek egyet“ és (7) “teljes mértékben egyetértek“.

| egyáltalán nem értek egyet = | 1 | 2 | 3 | 4 | 5 | 6 | 7 | = teljes mértékben egyetértek |
| --- | --- | --- | --- | --- | --- | --- | --- | --- |

| A sugar kapcsolat jó dolog, mert segít, hogy a sugar baby/boy kielégítő anyagi helyzetben legyen. | \| 1 \| 2 \| 3 \| 4 \| 5 \| 6 \| 7 \| \| --- \| --- \| --- \| --- \| --- \| --- \| --- \| |
| --- | --- | --- | --- | --- | --- | --- | --- | --- |
| Elképzelhető, hogy a jövőben részt veszek sugar kapcsolatban | \| 1 \| 2 \| 3 \| 4 \| 5 \| 6 \| 7 \| \| --- \| --- \| --- \| --- \| --- \| --- \| --- \| |
| Ha tudnám, hogy nem jár negatív megítéléssel és következményekkel, akkor ki szeretném próbálni a sugar kapcsolatot. | \| 1 \| 2 \| 3 \| 4 \| 5 \| 6 \| 7 \| \| --- \| --- \| --- \| --- \| --- \| --- \| --- \| |
| Ha előnyös lenne az életem alakulása szempontjából, elgondolkodnék azon, hogy részt vegyek sugar kapcsolatban. | \| 1 \| 2 \| 3 \| 4 \| 5 \| 6 \| 7 \| \| --- \| --- \| --- \| --- \| --- \| --- \| --- \| |
| Komolyan fontolóra venném a sugar kapcsolat kialakítását, ha ez ha tartós anyagi biztonsághoz juttatna. | \| 1 \| 2 \| 3 \| 4 \| 5 \| 6 \| 7 \| \| --- \| --- \| --- \| --- \| --- \| --- \| --- \| |

A következő állítások ismerősnek tűnhetnek. De most kérjük, értékelje a következő állításokat úgy, mintha **sugar daddy/mommy** lenne, vagyis olyan ember lenne, aki anyagi forrásokat biztosít partnere társaságáért cserébe. Kérjük, jelölje az alábbi állítások után, hogy mennyire ért egyet velük egy hétfokú skála segítségével, ahol (1) “egyáltalán nem értek egyet“ és (7) “teljes mértékben egyetértek“.

| egyáltalán nem értek egyet = | 1 | 2 | 3 | 4 | 5 | 6 | 7 | = teljes mértékben egyetértek |
| --- | --- | --- | --- | --- | --- | --- | --- | --- |

| A sugar kapcsolat jó dolog, mert segít, hogy az emberek jobban érezzék magukat. | \| 1 \| 2 \| 3 \| 4 \| 5 \| 6 \| 7 \| \| --- \| --- \| --- \| --- \| --- \| --- \| --- \| |
| --- | --- | --- | --- | --- | --- | --- | --- | --- |
| Elképzelhető, hogy a jövőben részt veszek sugar kapcsolatban. | \| 1 \| 2 \| 3 \| 4 \| 5 \| 6 \| 7 \| \| --- \| --- \| --- \| --- \| --- \| --- \| --- \| |
| Ha tudnám, hogy nem jár negatív megítéléssel és következményekkel, akkor ki szeretném próbálni a sugar kapcsolatot. | \| 1 \| 2 \| 3 \| 4 \| 5 \| 6 \| 7 \| \| --- \| --- \| --- \| --- \| --- \| --- \| --- \| |
| Ha előnyös lenne a szexuális életem vagy a külső megítélésem szempontjából, elgondolkodnék azon, hogy részt vegyek sugar kapcsolatban. | \| 1 \| 2 \| 3 \| 4 \| 5 \| 6 \| 7 \| \| --- \| --- \| --- \| --- \| --- \| --- \| --- \| |
| Komolyan fontolóra venném sugar kapcsolat kialakítását, ha így egy számomra minden igényt kielégítő partnert találhatnék. | \| 1 \| 2 \| 3 \| 4 \| 5 \| 6 \| 7 \| \| --- \| --- \| --- \| --- \| --- \| --- \| --- \| |

Language: Italian

**Country: Italy**

Una **relazione sugar** è una relazione sessuale reciprocamente vantaggiosa nella quale un/una partner più grande e più ricco/a (paparino o sugar daddy/mammina o sugar mommy) fornisce beni materiali ad un/una partner più giovane (ragazza sugar o sugar baby/ragazzo sugar o sugar boy) in cambio della sua compagnia. I/Le partner solitamente si incontrano per passare del tempo libero insieme e hanno rapporti sessuali solo se entrambi/e sono d’accordo.   Per favore, rispondi alle seguenti domande come se **tu fossi una ragazza sugar/un ragazzo sugar**, cioè, una persona che riceve beni materiali per la sua compagnia. Per favore, indica in che misura sei d'accordo con ogni affermazione sottostante utilizzando la scala di risposta a 7 livelli che va da (1) “assolutamente in disaccordo” a (7) “assolutamente in accordo ”.

| assolutamente in disaccordo = | 1 | 2 | 3 | 4 | 5 | 6 | 7 | = assolutamente d'accordo |
| --- | --- | --- | --- | --- | --- | --- | --- | --- |

| Una "relazione sugar" è una buona cosa perchè può aiutare la ragazza sugar/il ragazzo sugar ad avere una condizione economica soddisfacente. | \| 1 \| 2 \| 3 \| 4 \| 5 \| 6 \| 7 \| \| --- \| --- \| --- \| --- \| --- \| --- \| --- \| |
| --- | --- | --- | --- | --- | --- | --- | --- | --- |
| In futuro, potrei finire per impegnarmi in una "relazione sugar". | \| 1 \| 2 \| 3 \| 4 \| 5 \| 6 \| 7 \| \| --- \| --- \| --- \| --- \| --- \| --- \| --- \| |
| Se sapessi di non andare incontro a giudizi o conseguenze negative, mi piacerebbe provare una "relazione sugar". | \| 1 \| 2 \| 3 \| 4 \| 5 \| 6 \| 7 \| \| --- \| --- \| --- \| --- \| --- \| --- \| --- \| |
| Se fosse vantaggioso per la mia carriera lavorativa, considererei di impegnarmi in una "relazione sugar". | \| 1 \| 2 \| 3 \| 4 \| 5 \| 6 \| 7 \| \| --- \| --- \| --- \| --- \| --- \| --- \| --- \| |
| Considererei seriamente di impegnarmi in una "relazione sugar" se pensassi che potesse aiutarmi ad avere una condizione economica migliore. | \| 1 \| 2 \| 3 \| 4 \| 5 \| 6 \| 7 \| \| --- \| --- \| --- \| --- \| --- \| --- \| --- \| |

Le seguenti affermazioni potrebbere sembrare simili. Valuta le seguenti affermazioni come se **fossi un paparino (sugar daddy) o una mammina (sugar mommy)**, cioè una persona che fornisce vantaggi economici e/o pratici in cambio della compagnia del/della suo/a partner’ Indica in che misura sei d'accordo con ogni affermazione sottostante utilizzando la scala di risposta che va da (1) “assolutamente in disaccordo” a (7) “assolutamente d'accordo”.

| assolutamente in disaccordo = | 1 | 2 | 3 | 4 | 5 | 6 | 7 | = assolutamente d'accordo |
| --- | --- | --- | --- | --- | --- | --- | --- | --- |

| Una "relazione sugar" è una buona cosa perchè può aiutare le persone a sentirsi meglio. | \| 1 \| 2 \| 3 \| 4 \| 5 \| 6 \| 7 \| \| --- \| --- \| --- \| --- \| --- \| --- \| --- \| |
| --- | --- | --- | --- | --- | --- | --- | --- | --- |
| In futuro, potrei finire per impegnarmi in una "relazione sugar". | \| 1 \| 2 \| 3 \| 4 \| 5 \| 6 \| 7 \| \| --- \| --- \| --- \| --- \| --- \| --- \| --- \| |
| Se sapessi di non andare incontro a commenti o conseguenze negative, mi piacerebbe provare una "relazione sugar". | \| 1 \| 2 \| 3 \| 4 \| 5 \| 6 \| 7 \| \| --- \| --- \| --- \| --- \| --- \| --- \| --- \| |
| Se fosse vantaggioso per la mia vita sessuale o per la mia reputazione, considererei di impegnarmi in una "relazione sugar". | \| 1 \| 2 \| 3 \| 4 \| 5 \| 6 \| 7 \| \| --- \| --- \| --- \| --- \| --- \| --- \| --- \| |
| Considererei seriamente di impegnarmi in una "relazione sugar" se fosse il modo per trovare un/a partner che soddisfacesse tutti i miei bisogni. | \| 1 \| 2 \| 3 \| 4 \| 5 \| 6 \| 7 \| \| --- \| --- \| --- \| --- \| --- \| --- \| --- \| |

Language: Japanese

**Country: Japan**

**シュガーリレーションシップ**とは、年上で裕福な相手（シュガーダディ／マミィ）が若い人物（シュガーベイビー／ボーイ）に対して交際の見返りとして物的な資源（金銭や様々なプレゼントなど）を渡すという、取引型の関係のことをいいます。こうした関係にある2人は、通常休日を一緒に過ごしますが、互いが同意した場合には性的な関係を持つことがあります。   ここで、 もし**あなたがシュガーベイビー（交際の見返りに物的資源を受け取る若い人）であった**としたら、以下の文章に対してどのように思いますか？各文章が、あなたの考えにどのくらい当てはまるかを、（1）“まったく当てはまらない”から（7）“非常によく当てはまる”までの7段階の選択肢から選んで下さい。

| まったく当てはまらない = | 1 | 2 | 3 | 4 | 5 | 6 | 7 | = 非常によく当てはまる |
| --- | --- | --- | --- | --- | --- | --- | --- | --- |

| シュガーリレーションシップは、シュガーベイビー／ボーイの経済状況を充足させるのに役立つので、良いことである。 | \| 1 \| 2 \| 3 \| 4 \| 5 \| 6 \| 7 \| \| --- \| --- \| --- \| --- \| --- \| --- \| --- \| |
| --- | --- | --- | --- | --- | --- | --- | --- | --- |
| 将来、私はシュガーリレーションシップを利用する可能性がある。 | \| 1 \| 2 \| 3 \| 4 \| 5 \| 6 \| 7 \| \| --- \| --- \| --- \| --- \| --- \| --- \| --- \| |
| もしネガティブな判断・結果を下されたり招いたりすることがないとわかれば、私はシュガーリレーションシップを試すだろう。 | \| 1 \| 2 \| 3 \| 4 \| 5 \| 6 \| 7 \| \| --- \| --- \| --- \| --- \| --- \| --- \| --- \| |
| もし自分のキャリアに役立つのなら、私はシュガーリレーションシップの利用を検討するだろう。 | \| 1 \| 2 \| 3 \| 4 \| 5 \| 6 \| 7 \| \| --- \| --- \| --- \| --- \| --- \| --- \| --- \| |
| もし自分の経済状況を好転させられると思えたら、私はシュガーリレーションシップの利用を真剣に考えるだろう。 | \| 1 \| 2 \| 3 \| 4 \| 5 \| 6 \| 7 \| \| --- \| --- \| --- \| --- \| --- \| --- \| --- \| |

以下の文章は、先ほどのものと似ています。しかし今度は、もし**あなたがシュガーダディ／マミィ（交際の見返りに物的資源を提供する人）であったら**と考えて、回答して下さい。各文章が、あなたの考えにどのくらい当てはまるかを、（1）“まったく当てはまらない”から（7）“非常によく当てはまる”までの7段階の選択肢から選んで下さい。

| まったく当てはまらない = | 1 | 2 | 3 | 4 | 5 | 6 | 7 | = 非常によく当てはまる |
| --- | --- | --- | --- | --- | --- | --- | --- | --- |

| シュガーリレーションシップは、人々を良い気分にするので、良いことだと思う。 | \| 1 \| 2 \| 3 \| 4 \| 5 \| 6 \| 7 \| \| --- \| --- \| --- \| --- \| --- \| --- \| --- \| |
| --- | --- | --- | --- | --- | --- | --- | --- | --- |
| 将来、私はシュガーリレーションシップを利用する可能性がある。 | \| 1 \| 2 \| 3 \| 4 \| 5 \| 6 \| 7 \| \| --- \| --- \| --- \| --- \| --- \| --- \| --- \| |
| もしネガティブな判断・結果を下されたり招いたりすることがないとわかれば、私はシュガーリレーションシップを試すだろう。 | \| 1 \| 2 \| 3 \| 4 \| 5 \| 6 \| 7 \| \| --- \| --- \| --- \| --- \| --- \| --- \| --- \| |
| もし自分の性生活や他人からの評価にプラスになるのであれば、私はシュガーリレーションシップの利用を検討するだろう。 | \| 1 \| 2 \| 3 \| 4 \| 5 \| 6 \| 7 \| \| --- \| --- \| --- \| --- \| --- \| --- \| --- \| |
| もし自分の欲求を全て満たしてくれる相手を見つけられるのなら、私はシュガーリレーションシップの利用を真剣に考えるだろう。 | \| 1 \| 2 \| 3 \| 4 \| 5 \| 6 \| 7 \| \| --- \| --- \| --- \| --- \| --- \| --- \| --- \| |

Language: Korean

**Country: South Korea**

**조건만남**이란 나이가 많고 경제적으로 부유한 파트너(나이와 돈이 더 많은 중년 남성/여성)가 자신보다 어린 파트너(슈거 베이비/보이)에게 교제의 대가로 물질적 자원을 제공하는 거래적 성관계를 의미합니다. 이들은 보통 만나서 함께 여가시간을 보내고, 성행위는 양쪽 파트너가 모두 동의할 때만 갖습니다. **귀하가 슈거 베이비/보이**, 즉 파트너에게 교제의 대가로 물질적 자원을 받는 사람이라고 가정해보십시오. 다음의 각 진술문에 대하여 (1)“전혀 동의하지 않음”부터 (7)“전적으로 동의함”까지의 7점 척도를 사용하여 동의하시는 정도를 표시하십시오.

| 전혀 동의하지 않음 = | 1 | 2 | 3 | 4 | 5 | 6 | 7 | =전적으로 동의함 |
| --- | --- | --- | --- | --- | --- | --- | --- | --- |

| 조건만남(원조교제)은 슈거 베이비/보이로 하여금 만족스러운 재정상태를 갖도록 도와주기 때문에 좋은 것이다. | \| 1 \| 2 \| 3 \| 4 \| 5 \| 6 \| 7 \| \| --- \| --- \| --- \| --- \| --- \| --- \| --- \| |
| --- | --- | --- | --- | --- | --- | --- | --- | --- |
| 나는 앞으로 조건만남(원조교제)을 끝내게 될 것이다. | \| 1 \| 2 \| 3 \| 4 \| 5 \| 6 \| 7 \| \| --- \| --- \| --- \| --- \| --- \| --- \| --- \| |
| 내가 부정적 비판이나 결과에 처하게 되지 않는다면, 나는 조건만남(원조교제)을 시도해보고 싶다. | \| 1 \| 2 \| 3 \| 4 \| 5 \| 6 \| 7 \| \| --- \| --- \| --- \| --- \| --- \| --- \| --- \| |
| 내 경력에 도움이 된다면, 나는 조건만남(원조교제) 갖는 것을 생각해볼 것이다. | \| 1 \| 2 \| 3 \| 4 \| 5 \| 6 \| 7 \| \| --- \| --- \| --- \| --- \| --- \| --- \| --- \| |
| 내가 더 나은 재정상태가 되도록 도움이 된다면, 나는 조건만남(원조교제) 갖는 것을 진지하게 고려해볼 것이다. | \| 1 \| 2 \| 3 \| 4 \| 5 \| 6 \| 7 \| \| --- \| --- \| --- \| --- \| --- \| --- \| --- \| |

다음 진술문들은 비슷해 보일 수 있습니다. 그러나 이번에는, **귀하가 슈거대디/마미**, 즉, 자신보다 어린 파트너에게 교제의 대가로 물질적 자원을 제공하는 돈 많은 남성/여성이라고 가정하고 다음의 진술문을 평가해주십시오. 다음의 각 진술문에 대하여 (1) “전혀 동의하지 않음”부터 (7) “전적으로 동의함”까지의 7점 척도를 사용하여 동의하시는 정도를 표시하십시오.

| 전혀 동의하지 않음 = | 1 | 2 | 3 | 4 | 5 | 6 | 7 | =전적으로 동의함 |
| --- | --- | --- | --- | --- | --- | --- | --- | --- |

| 조건만남(원조교제)은 사람들을 기분 좋게 해줄 수 있기 때문에 좋은 것이다. | \| 1 \| 2 \| 3 \| 4 \| 5 \| 6 \| 7 \| \| --- \| --- \| --- \| --- \| --- \| --- \| --- \| |
| --- | --- | --- | --- | --- | --- | --- | --- | --- |
| 나는 앞으로 조건만남(원조교제)을 끝내게 될 것이다. | \| 1 \| 2 \| 3 \| 4 \| 5 \| 6 \| 7 \| \| --- \| --- \| --- \| --- \| --- \| --- \| --- \| |
| 내가 부정적 비판이나 결과에 처하게 되지 않는다면, 나는 조건만남(원조교제)을 시도해보고 싶다 | \| 1 \| 2 \| 3 \| 4 \| 5 \| 6 \| 7 \| \| --- \| --- \| --- \| --- \| --- \| --- \| --- \| |
| 내 성생활이나 나에 대한 남들의 평가에 이익이 된다면, 나는 조건만남(원조교제) 갖는 것을 고려해볼 것이다 | \| 1 \| 2 \| 3 \| 4 \| 5 \| 6 \| 7 \| \| --- \| --- \| --- \| --- \| --- \| --- \| --- \| |
| 그것이 나의 모든 욕구를 충족시켜 줄 파트너를 찾는 방법이라면, 나는 조건만남(원조교제) 갖는 것을 진지하게 고려해볼 것이다. | \| 1 \| 2 \| 3 \| 4 \| 5 \| 6 \| 7 \| \| --- \| --- \| --- \| --- \| --- \| --- \| --- \| |

Language: Lithuanian

**Country: Lithuania**

„Cukriniai” santykiai (angl. *sugar relationship*)– tai seksualiniai santykiai abipusiu sutarimu, kuomet vyresnis ir turtingesnis partneris (cukrinis tėtukas/mamytė) materialiai aprūpina jaunesnį partnerį (cukrinę mažutę/berniuką) mainais į jo/jos draugiją. Partneriai paprastai susitinka drauge leisti laisvalaikį, o seksualine veikla užsiimama tik tada, kai abu partneriai sutinka. Prašome įvertinti kiekvieną teiginį taip, lyg **Jūs būtumėte cukrinė mažutė/berniukas**, t.y. asmuo, kuris gauna materialinį atlygį už savo draugiją. Prašomenurodyti, kiek Jūs sutinkate su kiekvienu žemiau pateiktu teiginiu, naudodami septynių balų skalę nuo (1) „visiškai nesutinku“ iki (7) „visiškai sutinku“.

| visiškai nesutinku = | 1 | 2 | 3 | 4 | 5 | 6 | 7 | = visiškai sutinku |
| --- | --- | --- | --- | --- | --- | --- | --- | --- |

| „Cukriniai” santykiai yra geras dalykas, nes jie padeda cukrinei mažutei/berniukui būti geroje finansinėje situacijoje. | \| 1 \| 2 \| 3 \| 4 \| 5 \| 6 \| 7 \| \| --- \| --- \| --- \| --- \| --- \| --- \| --- \| |
| --- | --- | --- | --- | --- | --- | --- | --- | --- |
| Ateityje aš galėčiau užmegzti „cukrinius” santykius. | \| 1 \| 2 \| 3 \| 4 \| 5 \| 6 \| 7 \| \| --- \| --- \| --- \| --- \| --- \| --- \| --- \| |
| Jei žinočiau, kad nesulauksiu neigiamų pasekmių, norėčiau išbandyti „cukrinius” santykius. | \| 1 \| 2 \| 3 \| 4 \| 5 \| 6 \| 7 \| \| --- \| --- \| --- \| --- \| --- \| --- \| --- \| |
| Jei tai padėtų mano karjerai, aš galvočiau apie įsitraukimą į „cukrinius” santykius. | \| 1 \| 2 \| 3 \| 4 \| 5 \| 6 \| 7 \| \| --- \| --- \| --- \| --- \| --- \| --- \| --- \| |
| Aš rimtai svarstyčiau apie įsitraukimą į „cukrinius” santykius, jei tai pagerintų mano finansinę situaciją. | \| 1 \| 2 \| 3 \| 4 \| 5 \| 6 \| 7 \| \| --- \| --- \| --- \| --- \| --- \| --- \| --- \| |

Toliau pateikti teiginiai gali atrodyti panašūs. Bet dabar prašome įvertinti teiginius lyg J**ūs pats (-i) būtumėte cukrinis tėvelis/mamytė**, t.y. asmuo, kuris teikiam aterialinį atlygį už savo partnerio (-ės) draugiją. Prašome nurodyti, kiek sutinkate su kiekvienu toliau pateiktu teiginiu naudodami septynių balų skalę nuo (1) „visiškainesutinku“ iki (7) „visiškai sutinku“.

| visiškai nesutinku = | 1 | 2 | 3 | 4 | 5 | 6 | 7 | = visiškai sutinku |
| --- | --- | --- | --- | --- | --- | --- | --- | --- |

| „Cukriniai” santykiai yra geras dalykas, nes padeda žmonėms pasijusti geriau. | \| 1 \| 2 \| 3 \| 4 \| 5 \| 6 \| 7 \| \| --- \| --- \| --- \| --- \| --- \| --- \| --- \| |
| --- | --- | --- | --- | --- | --- | --- | --- | --- |
| Ateityje aš galėčiau užmegzti „cukrinius” santykius. | \| 1 \| 2 \| 3 \| 4 \| 5 \| 6 \| 7 \| \| --- \| --- \| --- \| --- \| --- \| --- \| --- \| |
| Jei žinočiau, kad nesulauksiu neigiamų pasekmių, norėčiau išbandyti „cukrinius” santykius. | \| 1 \| 2 \| 3 \| 4 \| 5 \| 6 \| 7 \| \| --- \| --- \| --- \| --- \| --- \| --- \| --- \| |
| Jei tai būtų naudinga mano seksualiniam gyvenimui ar tam, kaip mane mato kiti, aš galvočiau apie įsitraukimą į „cukrinius” santykius. | \| 1 \| 2 \| 3 \| 4 \| 5 \| 6 \| 7 \| \| --- \| --- \| --- \| --- \| --- \| --- \| --- \| |
| Aš rimtai svarstyčiau apie įsitraukimą I „cukrinius” santykius, jei tai būtų būdas rasti partnerį (-ę), atliepiantį (-čią) visus mano poreikius. | \| 1 \| 2 \| 3 \| 4 \| 5 \| 6 \| 7 \| \| --- \| --- \| --- \| --- \| --- \| --- \| --- \| |

Language: Macedonian

**Country: Macedonia**

**„Спонзорска врска“** е сексуална врска, во која постар/а и побогат/а партнер/ка („спонзор/ка“) обезбедува материјални средства за помлад/а партнер/ка („женска/машка спонзоруша“ или „спонзорирано лице“), а за возврат ја добива нејзината/неговата придружба. Партнерите се среќаваат за задоволство, а сексуалната активност е вклучена само кога двата партнери се согласни.   Ве молиме оценете ги следните искази, од перспектива на **„спонзорирано лице“**, т.е. личност која добива материјални услуги за нејзината придружба и наклонетост. Ве молиме назначете го степенот до кој (не) се согласувате со секој од долунаведените искази, користејќи 7-степена скала, од (1) “апсолутно не се согласувам” до (7) “апсолутно се согласувам”.

| апсолутно не се согласувам = | 1 | 2 | 3 | 4 | 5 | 6 | 7 | = апсолутно се согласувам |
| --- | --- | --- | --- | --- | --- | --- | --- | --- |

| „Спонзорската врска“ е добра работа затоа што може да му помогне на „спонзорираното лице“ да има завидна финансиска состојба. | \| 1 \| 2 \| 3 \| 4 \| 5 \| 6 \| 7 \| \| --- \| --- \| --- \| --- \| --- \| --- \| --- \| |
| --- | --- | --- | --- | --- | --- | --- | --- | --- |
| Во иднина, би можел/а да завршам во „спонзорска врска“. | \| 1 \| 2 \| 3 \| 4 \| 5 \| 6 \| 7 \| \| --- \| --- \| --- \| --- \| --- \| --- \| --- \| |
| Да знаев дека нема да сносам негативни последици или осуди, јас би сакал/а да пробам „спонзорска врска“. | \| 1 \| 2 \| 3 \| 4 \| 5 \| 6 \| 7 \| \| --- \| --- \| --- \| --- \| --- \| --- \| --- \| |
| Доколку би било корисно за мојата кариера, би размислил/а за впуштање во „спонзорска врска“. | \| 1 \| 2 \| 3 \| 4 \| 5 \| 6 \| 7 \| \| --- \| --- \| --- \| --- \| --- \| --- \| --- \| |
| Сериозно би размислил/а за впуштање во „спонзорска врска“ ако знаев дека тоа би ми помогнало да имам подобра финансиска состојба. | \| 1 \| 2 \| 3 \| 4 \| 5 \| 6 \| 7 \| \| --- \| --- \| --- \| --- \| --- \| --- \| --- \| |

Следните искази можат да ви изгледаат слични со претходните. Сепак, ве замолуваме оценете ги следните искази од перспектива на **„спонзор/ка“,** т.е. личност која обезбедува материјални услуги во замена за придружба и наклонетост од партнер/ка. Ве молиме назначете го степенот до кој (не) се согласувате со секој од наведените искази, користејќи ја 7-степената скала, од (1) “апсолутно не се согласувам” до (7) “апсолутно се согласувам”.

| апсолутно не се согласувам = | 1 | 2 | 3 | 4 | 5 | 6 | 7 | = апсолутно се согласувам |
| --- | --- | --- | --- | --- | --- | --- | --- | --- |

| „Спонзорска врска“ е добра работа зашто им помага на луѓето да се чувствуваат подобро. | \| 1 \| 2 \| 3 \| 4 \| 5 \| 6 \| 7 \| \| --- \| --- \| --- \| --- \| --- \| --- \| --- \| |
| --- | --- | --- | --- | --- | --- | --- | --- | --- |
| Во иднина би можел/а да завршам во „спонзорска врска“. | \| 1 \| 2 \| 3 \| 4 \| 5 \| 6 \| 7 \| \| --- \| --- \| --- \| --- \| --- \| --- \| --- \| |
| Да знаев дека нема да сносам негативни последици или осуди, јас би сакал/а да пробам „спонзорска врска“. | \| 1 \| 2 \| 3 \| 4 \| 5 \| 6 \| 7 \| \| --- \| --- \| --- \| --- \| --- \| --- \| --- \| |
| Доколку тоа би било корисно за мојот сексуален живот или за претставата што другите би ја имале за мене, јас би ја зел/а предвид можноста да се впуштам во „спонзорска врска“ | \| 1 \| 2 \| 3 \| 4 \| 5 \| 6 \| 7 \| \| --- \| --- \| --- \| --- \| --- \| --- \| --- \| |
| Сериозно би размислил/а за впуштање во „спонзорска врска“ ако тоа е начин да си најдам партнер/ка кој/а би можел/а да ги задоволи сите мои потреби. | \| 1 \| 2 \| 3 \| 4 \| 5 \| 6 \| 7 \| \| --- \| --- \| --- \| --- \| --- \| --- \| --- \| |

Language: Malay

**Country: Malaysia**

A **"Perhubungan Sugar"** adalah perhubungan secara seksual berbentuk urusniaga di mana pasangan yang lebih tua atau berharta (sugar mummy/daddy) menyediakan sumber-sumber berbentuk material kepada pasangan yang lebih muda (sugar baby/boy) sebagai balasan terhadap perhubungan yang terjalin. Pasangan selalunya berjumpa dan menghabiskan masa bersama, dan aktiviti seksual berlaku jika kedua-duanya bersetuju. Sila nilaikan kenyataan berikut seolah **anda adalah sugar baby/boy**, iaitu, seseorang yang menerima balasan berbentuk material di atas kemesraan mereka bersama. Sila tentukan setakat manakah anda setuju dengan setiap kenyataan di bawah dengan menggunakan tujuh poin nilai daripada (1) “ Sangat tidak setuju ” hingga (7) “Sangat setuju”.

| sama sekali tidak setuju = | 1 | 2 | 3 | 4 | 5 | 6 | 7 | = sangat setuju |
| --- | --- | --- | --- | --- | --- | --- | --- | --- |

| ‘Perhubungan sugar’ adalah sesuatu yang baik kerana ia membantu ‘sugar baby/boy’ mempunyai kewangan yang memuaskan | \| 1 \| 2 \| 3 \| 4 \| 5 \| 6 \| 7 \| \| --- \| --- \| --- \| --- \| --- \| --- \| --- \| |
| --- | --- | --- | --- | --- | --- | --- | --- | --- |
| Di masa hadapan, saya mungkin akan terlibat dalam ‘perhubungan sugar’ | \| 1 \| 2 \| 3 \| 4 \| 5 \| 6 \| 7 \| \| --- \| --- \| --- \| --- \| --- \| --- \| --- \| |
| Jika saya tahu ia tidak mempunyai pandangan atau kesan yang negatif, saya ingin mencuba menjalinkan ‘perhubungan sugar’ | \| 1 \| 2 \| 3 \| 4 \| 5 \| 6 \| 7 \| \| --- \| --- \| --- \| --- \| --- \| --- \| --- \| |
| Jika ianya menguntungkan karier saya, saya mungkin melibatkan diri dalam ‘perhubungan sugar’ | \| 1 \| 2 \| 3 \| 4 \| 5 \| 6 \| 7 \| \| --- \| --- \| --- \| --- \| --- \| --- \| --- \| |
| Saya mungkin akan mempertimbangkan dengan serius untuk melibatkan diri dalam ‘perhubungan sugar' jika saya rasa ia boleh membantu saya mempunyai kewangan yang lebih baik | \| 1 \| 2 \| 3 \| 4 \| 5 \| 6 \| 7 \| \| --- \| --- \| --- \| --- \| --- \| --- \| --- \| |

Kenyataan berikut mungkin kelihatan serupa. Tetapi, kali ini, sila nilaikan kenyataan seolah-olah anda **adalah sugar daddy/Mummy**, iaitu, seseorang yang akan menyediakan balasan berbentuk material kepada "pasangan sugar" mereka.’ Sila tentukan setakat manakah anda setuju dengan setiap kenyataan di bawah dengan menggunakan tujuh poin nilai daripada (1) “sangat tidak setuju” hingga (7) “sangat setuju”.

| sama sekali tidak setuju = | 1 | 2 | 3 | 4 | 5 | 6 | 7 | = sangat setuju |
| --- | --- | --- | --- | --- | --- | --- | --- | --- |

| ‘Perhubungan sugar’ adalah sesuatu yang baik kerana ia membantu orang lain untuk merasa lebih selesa | \| 1 \| 2 \| 3 \| 4 \| 5 \| 6 \| 7 \| \| --- \| --- \| --- \| --- \| --- \| --- \| --- \| |
| --- | --- | --- | --- | --- | --- | --- | --- | --- |
| Di masa hadapan, saya mungkin akan terlibat dalam ‘perhubungan sugar’ | \| 1 \| 2 \| 3 \| 4 \| 5 \| 6 \| 7 \| \| --- \| --- \| --- \| --- \| --- \| --- \| --- \| |
| Jika saya tahu ia tidak mempunyai pandangan atau kesan yang negatif, saya ingin mencuba menjalinkan ‘perhubungan sugar’ | \| 1 \| 2 \| 3 \| 4 \| 5 \| 6 \| 7 \| \| --- \| --- \| --- \| --- \| --- \| --- \| --- \| |
| Jika ianya bermanfaat kepada kehidupan seksual saya atau pandangan orang lain terhadap saya, saya akan mempertimbangkan untuk menjalinkan ‘perhubungan sugar’ | \| 1 \| 2 \| 3 \| 4 \| 5 \| 6 \| 7 \| \| --- \| --- \| --- \| --- \| --- \| --- \| --- \| |
| Saya mungkin akan mempertimbangkan dengan serius untuk melibatkan diri dalam ‘perhubungan sugar’ jika ia adalah cara untuk mendapatkan pasangan yang memenuhi segala keperluan saya. | \| 1 \| 2 \| 3 \| 4 \| 5 \| 6 \| 7 \| \| --- \| --- \| --- \| --- \| --- \| --- \| --- \| |

Language: Norwegian

**Country: Norway**

Et **sugardate-forhold** er et seksuelt bytteforhold der en eldre og rikere partner (sugar daddy eller sugar mama) sørger for materielle ressurser til en yngre partner (suger babe/boy) i retur for hans eller hennes selskap. Partnerne møtes vanligvis for å tilbringe tid sammen, og seksuell aktivitet er bare involvert dersom begge parter samtykker til det. Vennligst ta stilling til påstandene under som om **du var en suger babe/boy**, d.v.s. en person som får materielle ressurser i retur for ditt selskap. Vennligst vis i hvilken grad du er enig i påstandene under ved å bruke 7-punkts skalaen som går fra (1) “helt uenig ” til (7) “helt enig”.

| helt uenig = | 1 | 2 | 3 | 4 | 5 | 6 | 7 | = helt enig |
| --- | --- | --- | --- | --- | --- | --- | --- | --- |

| Et sugerdate-forhold er bra, siden det kan hjelpe den som er sugerbabe/boy til å ha en tilfredsstillende økonomi | \| 1 \| 2 \| 3 \| 4 \| 5 \| 6 \| 7 \| \| --- \| --- \| --- \| --- \| --- \| --- \| --- \| |
| --- | --- | --- | --- | --- | --- | --- | --- | --- |
| Jeg kan godt ende opp med å ha et sugerdate-forhold i fremtiden | \| 1 \| 2 \| 3 \| 4 \| 5 \| 6 \| 7 \| \| --- \| --- \| --- \| --- \| --- \| --- \| --- \| |
| Dersom jeg visste det ikke vil medføre fordømmelse eller negative konsekvenser, vil jeg vurdere å prøve et sugerdate- forhold. | \| 1 \| 2 \| 3 \| 4 \| 5 \| 6 \| 7 \| \| --- \| --- \| --- \| --- \| --- \| --- \| --- \| |
| Dersom det vil være fordelaktig for karierren min, vil jeg vurdere å engasjere meg i et sugardate-forhold | \| 1 \| 2 \| 3 \| 4 \| 5 \| 6 \| 7 \| \| --- \| --- \| --- \| --- \| --- \| --- \| --- \| |
| Jeg vil seriøst vurdere å involvere meg i et sugerdate-forhold dersom jeg tror det vil hjelpe meg økonomisk | \| 1 \| 2 \| 3 \| 4 \| 5 \| 6 \| 7 \| \| --- \| --- \| --- \| --- \| --- \| --- \| --- \| |

De følgende utsagnene virker kanskje like. Men vennligst vurder dem som om **du var en sugerdaddy/mama**, d.v.s. en person som gir materielle ressurser i bytte for partnerens selskap. Vennligst vis i hvilken grad du er enig i utsagnene ved å bruke 7-punkts skalaen som går fra (1) “helt uenig” til (7) “helt enig”.

| helt uenig = | 1 | 2 | 3 | 4 | 5 | 6 | 7 | = helt enig |
| --- | --- | --- | --- | --- | --- | --- | --- | --- |

| Det er bra å ha et sugardate-forhold, for det får en til å føle seg bedre | \| 1 \| 2 \| 3 \| 4 \| 5 \| 6 \| 7 \| \| --- \| --- \| --- \| --- \| --- \| --- \| --- \| |
| --- | --- | --- | --- | --- | --- | --- | --- | --- |
| Jeg kan godt ende opp med å ha et sugardate-forhold i fremtiden | \| 1 \| 2 \| 3 \| 4 \| 5 \| 6 \| 7 \| \| --- \| --- \| --- \| --- \| --- \| --- \| --- \| |
| Dersom jeg visste at det ikke ville føre til fordømmelse eller negative konsekvenser, ønsker jeg å forsøke sugardating | \| 1 \| 2 \| 3 \| 4 \| 5 \| 6 \| 7 \| \| --- \| --- \| --- \| --- \| --- \| --- \| --- \| |
| Dersom det vil være fordelaktig for mitt seksualliv eller for andres oppfatning av meg, vil jeg vurdere å ha et sugerdate-forhold | \| 1 \| 2 \| 3 \| 4 \| 5 \| 6 \| 7 \| \| --- \| --- \| --- \| --- \| --- \| --- \| --- \| |
| Jeg vil seriøst vurdere å involvere meg i et sugerdate-forhold dersom det er en måte å finne en partner som vil tilfredsstille alle mine behov på | \| 1 \| 2 \| 3 \| 4 \| 5 \| 6 \| 7 \| \| --- \| --- \| --- \| --- \| --- \| --- \| --- \| |

Language: Persian

**Country: Iran**

رابطه شوگر یک رابطه جنسی معاملاتی است که در آن یک پاتنر مسن تر و ثروتمندتر (شوگر ددی/ مامی) در ازای معاشرت با وی، منابع مادی یک شریک جوان را فراهم می کند. پارتنرها معمولاً برای گذران اوقات فراغت خود با هم ملاقات می کنند و فعالیت جنسی فقط در صورت رضایت هر دو طرف صورت می پذیرد. لطفاً جمله های زیر را به گونه ای ارزیابی کنید که گویا شما یک پارتنر جوان هستید، یعنی شخصی که منابع مادی را برای همراهی خود دریافت می کند. لطفاً میزان موافقت خود را با هر یک از عبارات زیر با استفاده از مقیاسهای هفت امتیازی از (1) "کاملاً مخالف" تا (7) "کاملاً موافق" نشان دهید.

| کاملا موافقم = | 7 | 6 | 5 | 4 | 3 | 2 | 1 | = کاملا مخالفم |
| --- | --- | --- | --- | --- | --- | --- | --- | --- |

| رابطه ی شوگری رابطه ی خوبی است زیرا می تواند به پارتنر جوان کمک کند تا از نظر مالی وضعیت مطلوبی داشته باشد. () | \| 7 \| 6 \| 5 \| 4 \| 3 \| 2 \| 1 \| \| --- \| --- \| --- \| --- \| --- \| --- \| --- \| |
| --- | --- | --- | --- | --- | --- | --- | --- | --- |
| در آینده می توانم در نهایت درگیر یک رابطه شوگر شوم. () | \| 7 \| 6 \| 5 \| 4 \| 3 \| 2 \| 1 \| \| --- \| --- \| --- \| --- \| --- \| --- \| --- \| |
| اگر می دانستم که مورد قضاوت یا عواقب منفی قرار نخواهم گرفت ، دوست داشتم یک رابطه شوگر را امتحان کنم. () | \| 7 \| 6 \| 5 \| 4 \| 3 \| 2 \| 1 \| \| --- \| --- \| --- \| --- \| --- \| --- \| --- \| |
| اگر به سود شغلم باشد، به درگیری در یک رابطه شوگر فکر خواهم کنم. () | \| 7 \| 6 \| 5 \| 4 \| 3 \| 2 \| 1 \| \| --- \| --- \| --- \| --- \| --- \| --- \| --- \| |
| اگر فکر کنم که درگیری در یک رابطه اینچنینی به من کمک خواهد کرد تا از نظر مالی وضعیت بهتری داشته باشم، به طور جدی درگیری در این رابطه را در نظر خواهم گرفت. () | \| 7 \| 6 \| 5 \| 4 \| 3 \| 2 \| 1 \| \| --- \| --- \| --- \| --- \| --- \| --- \| --- \| |

عبارات زیر ممکن است مشابه به نظر برسند. اما اکنون ، لطفاً اظهارات زیر را چنان ارزیابی کنید که گویی شما یک شوگر ددی/ شوگر مامی هستید، یعنی شخصی که منابع مادی را برای همراهی پارتنر خود فراهم می کند.

لطفاً میزان موافقت خود را با هر یک از عبارات زیر با استفاده از مقیاسهای هفت امتیازی از (1) "کاملاً مخالف" تا (7) "کاملاً موافق" نشان دهید.

| کاملا موافقم = | 7 | 6 | 5 | 4 | 3 | 2 | 1 | = کاملا مخالفم |
| --- | --- | --- | --- | --- | --- | --- | --- | --- |

| رابطه ی این چنینی چیز خوبی است زیرا می تواند به افراد کمک کند تا احساس بهتری داشته باشند. () | \| 7 \| 6 \| 5 \| 4 \| 3 \| 2 \| 1 \| \| --- \| --- \| --- \| --- \| --- \| --- \| --- \| |
| --- | --- | --- | --- | --- | --- | --- | --- | --- |
| در آینده می توانم در نهایت درگیر یک رابطه این چنینی شوم. () | \| 7 \| 6 \| 5 \| 4 \| 3 \| 2 \| 1 \| \| --- \| --- \| --- \| --- \| --- \| --- \| --- \| |
| اگر می دانستم که مورد قضاوت یا عواقب منفی قرار نخواهم گرفت ، دوست داشتم یک رابطه این چنینی را امتحان کنم. () | \| 7 \| 6 \| 5 \| 4 \| 3 \| 2 \| 1 \| \| --- \| --- \| --- \| --- \| --- \| --- \| --- \| |
| اگر این به نفع زندگی جنسی من یا قضاوت دیگران درباره من باشد، من درگیری در رابطه ی این چنینی را در نظر خواهم گرفت. () | \| 7 \| 6 \| 5 \| 4 \| 3 \| 2 \| 1 \| \| --- \| --- \| --- \| --- \| --- \| --- \| --- \| |
| اگر رابطه این چنینی راهی برای یافتن پارتنری باشد که تمام نیازهای من را برآورده کند، من به طور جدی درگیر این رابطه می شوم. | \| 7 \| 6 \| 5 \| 4 \| 3 \| 2 \| 1 \| \| --- \| --- \| --- \| --- \| --- \| --- \| --- \| |

Language: Polish

**Country: Poland**

**Sponsoring** to transakcyjna relacja seksualna, w której starszy i bogatszy partner (osoba sponsorująca; ang. sugar daddy/mommy) zapewnia dobra materialne młodszemu partnerowi (osobie sponsorowanej) w zamian za ich towarzystwo. Partnerzy zazwyczaj spotykają się, by wspólnie spędzać czas wolny, a aktywność seksualna ma miejsce tylko wtedy, gdy oboje wyrażą na to zgodę. Oceń proszę poniższe stwierdzenia tak, **jakbyś był osobą sponsorowaną**, tzn. osobą, która otrzymuje dobra materialne w zamian za towarzystwo. Wskaż stopień, w jakim zgadzasz się z każdym z poniższych stwierdzeń używając siedmiostopniowej skali od (1) “zdecydowanie się nie zgadzam” do (7) “zdecydowanie się zgadzam”.

| całkowicie się nie zgadzam = | 1 | 2 | 3 | 4 | 5 | 6 | 7 | = całkowicie się zgadzam |
| --- | --- | --- | --- | --- | --- | --- | --- | --- |

| Sponsorowana relacja jest dobrą rzeczą, ponieważ może pomóc sponsorowanej osobie osiągnąć satysfakcjonującą sytuację finansową. | \| 1 \| 2 \| 3 \| 4 \| 5 \| 6 \| 7 \| \| --- \| --- \| --- \| --- \| --- \| --- \| --- \| |
| --- | --- | --- | --- | --- | --- | --- | --- | --- |
| W przyszłości mógłbym/mogłabym zaangażować się w sponsorowaną relację. | \| 1 \| 2 \| 3 \| 4 \| 5 \| 6 \| 7 \| \| --- \| --- \| --- \| --- \| --- \| --- \| --- \| |
| Gdybym wiedział/a, że nie spotkają mnie żadne negatywne komentarze lub konsekwencje, chciałbym/chciałabym spróbować sponsorowanej relacji. | \| 1 \| 2 \| 3 \| 4 \| 5 \| 6 \| 7 \| \| --- \| --- \| --- \| --- \| --- \| --- \| --- \| |
| Gdyby to pomogło rozwinąć moją karierę, zastanowiłbym/zastanowiłabym się nad zaangażowaniem się w sponsorowaną relację. | \| 1 \| 2 \| 3 \| 4 \| 5 \| 6 \| 7 \| \| --- \| --- \| --- \| --- \| --- \| --- \| --- \| |
| Poważnie bym się zastanowił/a nad zaangażowaniem się w sponsorowaną relację, gdybym myśłał/a, że pomoże mi to poprawić moją sytuację finansową. | \| 1 \| 2 \| 3 \| 4 \| 5 \| 6 \| 7 \| \| --- \| --- \| --- \| --- \| --- \| --- \| --- \| |

Poniższe stwierdzenia mogą wydawać się podobne. Ale tym razem, oceń proszę je tak, **jakbyś był/a sponsorującą osobą**, tzn. osobą, która zapewnia dobra materialne w zamian za towarzystwo partnera/partnerki. Wskaż proszę, w jaki stopniu zgadzasz z każdym z poniższych stwierdzeń używając siedmiostopniowej skali od (1) “zdecydowanie się nie zgadzam” do (7) “zdecydowanie się zgadzam”.

| całkowicie się nie zgadzam = | 1 | 2 | 3 | 4 | 5 | 6 | 7 | = całkowicie się zgadzam |
| --- | --- | --- | --- | --- | --- | --- | --- | --- |

| Sponsorowana relacja jest czymś dobrym, ponieważ może pomóc ludziom czuć się lepiej. | \| 1 \| 2 \| 3 \| 4 \| 5 \| 6 \| 7 \| \| --- \| --- \| --- \| --- \| --- \| --- \| --- \| |
| --- | --- | --- | --- | --- | --- | --- | --- | --- |
| W przyszłości mógłbym/łabym zaangażować się w sponsorowaną relację. | \| 1 \| 2 \| 3 \| 4 \| 5 \| 6 \| 7 \| \| --- \| --- \| --- \| --- \| --- \| --- \| --- \| |
| Gdybym wiedział/a, że nie spotkają mnie żadne negatywne komentarze lub konsekwencje, chciałbym/chciałabym spróbować sponsorowanej relacji. | \| 1 \| 2 \| 3 \| 4 \| 5 \| 6 \| 7 \| \| --- \| --- \| --- \| --- \| --- \| --- \| --- \| |
| Gdyby to było korzystne dla mojego życia seksualnego lub dla poprawienia opinii innych na mój temat, rozważyłbym/rozważyłabym sponsorowaną relację. | \| 1 \| 2 \| 3 \| 4 \| 5 \| 6 \| 7 \| \| --- \| --- \| --- \| --- \| --- \| --- \| --- \| |
| Poważnie zastanowiłbym/łabym się nad sponsorowaną relacją, gdybym w ten sposób mógł/mogła znaleźć partnera/partnerkę, który/a by spełniał/a wszystkie moje potrzeby. | \| 1 \| 2 \| 3 \| 4 \| 5 \| 6 \| 7 \| \| --- \| --- \| --- \| --- \| --- \| --- \| --- \| |

Language: Portuguese

**Country: Portugal**

Uma "sugar relationship" é uma relação sexual transacional em que um/a parceiro/a mais velho/a e rico/a (designado/a "sugar daddy/mommy") oferece recursos materiais a um/a parceiro/a mais jovem (designado/a "sugar baby/boy"), em troca da sua companhia. Os/as parceiros/as geralmente encontram-se para passar momentos de lazer juntos e a atividade sexual só está envolvida se ambos derem o seu consentimento. Neste inquérito iremos usar estes estrangeirismos por serem usados com maior frequência. Por favor, avalie as seguintes afirmações como se **fosse um/a "sugar baby/boy"**, i.e., a pessoa que recebe recursos materiais por dar a sua companhia. Por favor, indique em que medida concorda com cada uma das seguintes afirmações usando uma escala de 7 pontos que varia entre (1) “discordo absolutamente” a (7) “concordo absolutamente”.

| Discordo totalmente = | 1 | 2 | 3 | 4 | 5 | 6 | 7 | = Concordo totalmente |
| --- | --- | --- | --- | --- | --- | --- | --- | --- |

| Um relacionamento de tipo "sugar" é bom porque pode ajudar a rapariga ou rapaz ("sugar baby/boy") a ter uma situação financeira mais satisfatória. | \| 1 \| 2 \| 3 \| 4 \| 5 \| 6 \| 7 \| \| --- \| --- \| --- \| --- \| --- \| --- \| --- \| |
| --- | --- | --- | --- | --- | --- | --- | --- | --- |
| No futuro, eu poderia acabar por me envolver numa relação "sugar". | \| 1 \| 2 \| 3 \| 4 \| 5 \| 6 \| 7 \| \| --- \| --- \| --- \| --- \| --- \| --- \| --- \| |
| Se soubesse que não haveria julgamentos ou consequências negativas, eu gostaria de tentar ter uma relação "sugar". | \| 1 \| 2 \| 3 \| 4 \| 5 \| 6 \| 7 \| \| --- \| --- \| --- \| --- \| --- \| --- \| --- \| |
| Se isso beneficiasse a minha carreira, eu pensaria em envolver-me numa relação "sugar". | \| 1 \| 2 \| 3 \| 4 \| 5 \| 6 \| 7 \| \| --- \| --- \| --- \| --- \| --- \| --- \| --- \| |
| Consideraria seriamente envolver-me numa relação "sugar", se achasse que me ajudaria a ter uma situação financeira melhor. | \| 1 \| 2 \| 3 \| 4 \| 5 \| 6 \| 7 \| \| --- \| --- \| --- \| --- \| --- \| --- \| --- \| |

As próximas questões podem parecer semelhantes. Mas agora, por favor avalie as seguintes afirmações como se **fosse um/a "sugar daddy/mother"**, i.e., a pessoa que sustenta materialmente o/a parceiro/a pela sua companhia. Por favor, indique em que medida concorda com cada uma das seguintes afirmações utilizando uma escala de 7 pontos que varia entre (1) “discordo totalmente” e (7) “concordo totalmente”.

| Discordo totalmente = | 1 | 2 | 3 | 4 | 5 | 6 | 7 | = Concordo totalmente |
| --- | --- | --- | --- | --- | --- | --- | --- | --- |

| Um relacionamento "sugar" é bom porque pode ajudar as pessoas a sentirem-se melhor. | \| 1 \| 2 \| 3 \| 4 \| 5 \| 6 \| 7 \| \| --- \| --- \| --- \| --- \| --- \| --- \| --- \| |
| --- | --- | --- | --- | --- | --- | --- | --- | --- |
| No futuro, eu poderia acabar por me envolver numa relação "sugar". | \| 1 \| 2 \| 3 \| 4 \| 5 \| 6 \| 7 \| \| --- \| --- \| --- \| --- \| --- \| --- \| --- \| |
| Se soubesse que não haveria julgamentos ou consequências negativas, eu gostaria de tentar ter uma relação "sugar". | \| 1 \| 2 \| 3 \| 4 \| 5 \| 6 \| 7 \| \| --- \| --- \| --- \| --- \| --- \| --- \| --- \| |
| Se isso fosse benéfico para a minha vida sexual ou para o julgamento que os/as outros/as fazem sobre mim, eu consideraria envolver-me numa relação "sugar". | \| 1 \| 2 \| 3 \| 4 \| 5 \| 6 \| 7 \| \| --- \| --- \| --- \| --- \| --- \| --- \| --- \| |
| Consideraria seriamente envolver-me numa relação "sugar" se essa fosse a forma de encontrar um/a parceiro/a que respondesse a todas as minhas necessidades. | \| 1 \| 2 \| 3 \| 4 \| 5 \| 6 \| 7 \| \| --- \| --- \| --- \| --- \| --- \| --- \| --- \| |

Language: Romanian

**Country: Romania, Moldova**

O **relație sugar** este o relație sexuală bazată pe schimb în care un partener mai bătrân și mai bogat (sugar mommy/ daddy) oferă resurse materiale unui partener mai tânăr (sugar baby/ boy) în schimb pentru compania ei sau a lui. Partenerii de obicei se întâlnesc pentru a petrece timp de plăcere împreună, iar activitățile sexuale sunt implicate doar dacă ambii parteneri sunt de acord.  Vă rugăm să dați un scor următoarelor afirmații ca și cum **ați fi dumneavoastră sugar baby/ boy**, adică o persoană care primește resurse materiale în schimbul companiei pe care o oferă. Vă rugăm indicați măsura în care sunteți de acord cu fiecare dintre afirmațiile de mai jos folosind o scală în șapte puncte care merge de la (1) “dezacord absolut” până la (7) “acord absolut”.

| dezacord total = | 1 | 2 | 3 | 4 | 5 | 6 | 7 | = acord total |
| --- | --- | --- | --- | --- | --- | --- | --- | --- |

| O relație de tip sugar este un lucru bun deoarece îl ajută pe sugar baby/ boy sa aibă o situație financiară satisfăcătoare | \| 1 \| 2 \| 3 \| 4 \| 5 \| 6 \| 7 \| \| --- \| --- \| --- \| --- \| --- \| --- \| --- \| |
| --- | --- | --- | --- | --- | --- | --- | --- | --- |
| În viitor, aș putea să mă angajez într-o relație de tip sugar | \| 1 \| 2 \| 3 \| 4 \| 5 \| 6 \| 7 \| \| --- \| --- \| --- \| --- \| --- \| --- \| --- \| |
| Dacă aș ști că nu ar exista consecințe negative și nu aș fi judecat mi-ar plăcea să încerc o relație de tip sugar | \| 1 \| 2 \| 3 \| 4 \| 5 \| 6 \| 7 \| \| --- \| --- \| --- \| --- \| --- \| --- \| --- \| |
| Dacă carierea mea ar avea de beneficiat m-aș angaja într-o relație de tip sugar | \| 1 \| 2 \| 3 \| 4 \| 5 \| 6 \| 7 \| \| --- \| --- \| --- \| --- \| --- \| --- \| --- \| |
| Aș lua serios în considerare să mă angajez într-o relație de tip sugar dacă m-ar ajuta să am o situație financiară mai bună | \| 1 \| 2 \| 3 \| 4 \| 5 \| 6 \| 7 \| \| --- \| --- \| --- \| --- \| --- \| --- \| --- \| |

Următoarele afirmații pot părea similare. Dar acum acordați un scor următoarelor afirmații ca și cum **dumneavoastră ați fi sugar daddy/mommy**, adică o persoană care furnizează resurse materiale partenerului său în schimbul’companiei. Vă rugăm indicați măsura în care sunteți de acord cu fiecare dintre afirmațiile de mai jos folosind o scală în șapte puncte de la (1) "dezacord absolut" până la (7) "acord absolut".

| dezacord total = | 1 | 2 | 3 | 4 | 5 | 6 | 7 | = acord total |
| --- | --- | --- | --- | --- | --- | --- | --- | --- |

| O relație de tip sugar este un lucru bun pentru că pot să ajut oamenii să se simtă mai bine | \| 1 \| 2 \| 3 \| 4 \| 5 \| 6 \| 7 \| \| --- \| --- \| --- \| --- \| --- \| --- \| --- \| |
| --- | --- | --- | --- | --- | --- | --- | --- | --- |
| În viitor, aș putea să ajung să mă implic într-o relație de tip sugar | \| 1 \| 2 \| 3 \| 4 \| 5 \| 6 \| 7 \| \| --- \| --- \| --- \| --- \| --- \| --- \| --- \| |
| Dacă aș ști că nu ar avea consecințe negative și nu aș fi judecat mi-ar plăcea să încep o relație de tip sugar | \| 1 \| 2 \| 3 \| 4 \| 5 \| 6 \| 7 \| \| --- \| --- \| --- \| --- \| --- \| --- \| --- \| |
| Dacă ar fi benefic pentru viața mea sexuală sau pentru modul în care alții mă judecă pe mine aș lua în considerare să mă implic într-o relație de tip sugar | \| 1 \| 2 \| 3 \| 4 \| 5 \| 6 \| 7 \| \| --- \| --- \| --- \| --- \| --- \| --- \| --- \| |
| Aș lua serios în considerare o relație de tip sugar dacă acesta ar fi un mod în care aș găsi un partener care să îndeplinească toate nevoile mele. | \| 1 \| 2 \| 3 \| 4 \| 5 \| 6 \| 7 \| \| --- \| --- \| --- \| --- \| --- \| --- \| --- \| |

Language: Russian

**Country: Russia**

**Отношения по расчету** - это взаимовыгодные отношения между партнерами, в которых старший и более богатый партнер (папик/мамочка) предоставляет материальные ресурсы молодому партнеру (содержанка/содержанец) в обмен на общение с ним/ней. Партнеры обычно встречаются, чтобы вместе провести свободное время, и сексуальная связь допускается только по обоюдному согласию партнеров. Оцените, пожалуйста, свою степень согласия со следующими утверждениями**, представив себя на месте содержанки/содержанца**, т.е. человека, который получает материальную выгоду в обмен на дружеское общение. Оцените степень своего согласия с каждым утверждением, приведённым ниже, используя 7-балльную шкалу от (1) “совершенно не согласен(-на)” до (7) “совершенно согласен(-на)”.

| совершенно не согласен(-на) = | 1 | 2 | 3 | 4 | 5 | 6 | 7 | = совершенно согласен(-на) |
| --- | --- | --- | --- | --- | --- | --- | --- | --- |

| Отношения по расчету - это хорошо, поскольку они обеспечивают содержанке/содержанцу достаток. | \| 1 \| 2 \| 3 \| 4 \| 5 \| 6 \| 7 \| \| --- \| --- \| --- \| --- \| --- \| --- \| --- \| |
| --- | --- | --- | --- | --- | --- | --- | --- | --- |
| В будущем я мог(-ла) бы вступить в отношения по расчету. | \| 1 \| 2 \| 3 \| 4 \| 5 \| 6 \| 7 \| \| --- \| --- \| --- \| --- \| --- \| --- \| --- \| |
| Если бы я знал(-а), что НЕ навлеку на себя осуждения или негативных последствий, я бы хотел(-а) попробовать вступить в отношения по расчету. | \| 1 \| 2 \| 3 \| 4 \| 5 \| 6 \| 7 \| \| --- \| --- \| --- \| --- \| --- \| --- \| --- \| |
| Я бы подумал(-а) о том, чтобы вступить в отношения по расчету, если бы это благотворно повлияло на мою карьеру. | \| 1 \| 2 \| 3 \| 4 \| 5 \| 6 \| 7 \| \| --- \| --- \| --- \| --- \| --- \| --- \| --- \| |
| Я бы всерьез задумался(-лась) о том, чтобы вступить в отношения по расчету, если бы это помогло мне улучшить финансовое положение. | \| 1 \| 2 \| 3 \| 4 \| 5 \| 6 \| 7 \| \| --- \| --- \| --- \| --- \| --- \| --- \| --- \| |

Следующие утверждения могут показаться похожими. Но теперь оцените следующие утверждения, как если бы **Вы были спонсором в отношениях по расчету**, т.е. человеком, который предоставляет материальные ресурсы для отношений со своим партнером. Укажите, насколько Вы согласны с каждым из приведенных ниже утверждений, используя семибалльную шкалу оценок от (1) “совершенно не согласен(-на)” до (7) “совершенно согласен(-на)”.

| совершенно не согласен(-на) = | 1 | 2 | 3 | 4 | 5 | 6 | 7 | = совершенно согласен(-на) |
| --- | --- | --- | --- | --- | --- | --- | --- | --- |

| Отношения по расчету это хорошо, потому что они могут помочь людям чувствовать себя лучше. | \| 1 \| 2 \| 3 \| 4 \| 5 \| 6 \| 7 \| \| --- \| --- \| --- \| --- \| --- \| --- \| --- \| |
| --- | --- | --- | --- | --- | --- | --- | --- | --- |
| В будущем я мог(-ла) бы вступить в отношения по расчету. | \| 1 \| 2 \| 3 \| 4 \| 5 \| 6 \| 7 \| \| --- \| --- \| --- \| --- \| --- \| --- \| --- \| |
| Если бы я знал(-а), что НЕ навлеку на себя осуждения или негативных последствий, я бы хотел(-а) попробовать вступить в отношения по расчету. | \| 1 \| 2 \| 3 \| 4 \| 5 \| 6 \| 7 \| \| --- \| --- \| --- \| --- \| --- \| --- \| --- \| |
| Я бы подумал(-а) о том, чтобы вступить в отношения по расчету, если это благотворно повлияет на мою сексуальную жизнь или мою репутацию. | \| 1 \| 2 \| 3 \| 4 \| 5 \| 6 \| 7 \| \| --- \| --- \| --- \| --- \| --- \| --- \| --- \| |
| Я бы всерьез задумался(-лась) о том, чтобы вступить в отношения по расчету, если бы это был способ найти партнера, который отвечал бы всем моим потребностям. | \| 1 \| 2 \| 3 \| 4 \| 5 \| 6 \| 7 \| \| --- \| --- \| --- \| --- \| --- \| --- \| --- \| |

Language: Serbian

**Country: Serbia, Montenegro**

Veza  **sa sponzor(k)om ili sponzorska veza (eng. sugar relationship)** je transakciona seksualna veza u kojoj stariji/a i bogatiji/a partner tj. sponzor ili partnerka - sponzorka (eng. sugar daddy/mommy) pruža materijalna sredstva mlađem partneru – sponzorašu ili sponzoruši (eng. sugar baby/boy) kao nadoknadu za druženje. Partneri se obično sastaju kako bi proveli slobodno vrijeme zajedno, a seksualne aktivnosti su uključene samo ako oba partnera daju svoj pristanak. Molimo ocijenite sljedeće izjave kao da ste vi **sugar baby/boy,** ,tj. osoba koja prima materijalna sredstva za svoje druženje. Navedite u kojoj mjeri se slažete sa svakom od sljedećih izjava koristeći skalu ocjenjivanja od sedam nivoa u rasponu od (1) “„apsolutno se ne slažem“” do (7) “„apsolutno se slažem“”.

| uopšte se ne slažem = | 1 | 2 | 3 | 4 | 5 | 6 | 7 | = potpuno se slažem |
| --- | --- | --- | --- | --- | --- | --- | --- | --- |

| Sponzorska veza (engl. sugar relationship) je dobra stvar jer može pomoći partneru koji je u vezi sa "sponzorom" da ima zadovoljavajuću finansijsku situaciju. | \| 1 \| 2 \| 3 \| 4 \| 5 \| 6 \| 7 \| \| --- \| --- \| --- \| --- \| --- \| --- \| --- \| |
| --- | --- | --- | --- | --- | --- | --- | --- | --- |
| U budućnosti bih mogao/la da budem u sponzorskoj vezi. | \| 1 \| 2 \| 3 \| 4 \| 5 \| 6 \| 7 \| \| --- \| --- \| --- \| --- \| --- \| --- \| --- \| |
| Kad bih znao/la da ne bi bilo negativnih osuda ili posljedica, želio/la bih da budem u sponzorskoj vezi. | \| 1 \| 2 \| 3 \| 4 \| 5 \| 6 \| 7 \| \| --- \| --- \| --- \| --- \| --- \| --- \| --- \| |
| Ako bi to koristilo mojoj karijeri, razmislio/la bih o tome da se upustim u sponzorsku vezu. | \| 1 \| 2 \| 3 \| 4 \| 5 \| 6 \| 7 \| \| --- \| --- \| --- \| --- \| --- \| --- \| --- \| |
| Ozbiljno bih razmotrio/la mogućnost sponzorske veze ako bih mislio/la da će to pomoći poboljšanju moje finansijske situacije. | \| 1 \| 2 \| 3 \| 4 \| 5 \| 6 \| 7 \| \| --- \| --- \| --- \| --- \| --- \| --- \| --- \| |

Sljedeće izjave mogu izgledati slično prethodnim. Ali sada, molimo Vas da ocijenite sljedeće izjave kao da **ste Vi sponzor ili sponzorka (engl. sugar daddy/mommy)**, tj. osoba koja pruža materijalna sredstva svom partneru/ki u zamjenu za ’druženje. Navedite u kojoj se mjeri slažete sa svakom od sljedećih izjava koristeći sedmostepene skale u rasponu od (1) “apsolutno se ne slažem” do (7) “apsolutno se slažem”.

| uopšte se ne slažem = | 1 | 2 | 3 | 4 | 5 | 6 | 7 | = potpuno se slažem |
| --- | --- | --- | --- | --- | --- | --- | --- | --- |

| Sponzorska veza je dobra stvar jer može pomoći ljudima da se bolje osjećaju. | \| 1 \| 2 \| 3 \| 4 \| 5 \| 6 \| 7 \| \| --- \| --- \| --- \| --- \| --- \| --- \| --- \| |
| --- | --- | --- | --- | --- | --- | --- | --- | --- |
| U budućnosti bih mogao/la započeti sponzorsku vezu. | \| 1 \| 2 \| 3 \| 4 \| 5 \| 6 \| 7 \| \| --- \| --- \| --- \| --- \| --- \| --- \| --- \| |
| Upustio/la bih se u sponzorsku vezu kada bih znao/la da na sebe neću navući negativne komentare ili posljedice. | \| 1 \| 2 \| 3 \| 4 \| 5 \| 6 \| 7 \| \| --- \| --- \| --- \| --- \| --- \| --- \| --- \| |
| Ozbiljno bih razmislio/la da se upustim u sponzorsku vezu ukoliko bi bila korisna za moj seksulani život ili bi doprinijela boljem mišljenju drugih o meni. | \| 1 \| 2 \| 3 \| 4 \| 5 \| 6 \| 7 \| \| --- \| --- \| --- \| --- \| --- \| --- \| --- \| |
| Ozbiljno bih razmotrio/la mogućnost sponzorske veze ako bih mislio/la da će mi to pomoći u pronalasku partnera/ice koji/ja bi zadovoljavao/la sve moje potrebe. | \| 1 \| 2 \| 3 \| 4 \| 5 \| 6 \| 7 \| \| --- \| --- \| --- \| --- \| --- \| --- \| --- \| |

Language: Slovak

**Country: Slovakia**

**Sladký vzťah** (v angličtine sugar relationship) je transakčný vzťah medzi pohlaviami, v ktorom starší a bohatší partner (označovaný ako sladký tato alebo sladká mamina - sugar daddy/mommy) poskytuje materiálne zdroje mladšiemu partnerovi (sladké dievča / chlapec - sugar baby/boy) na oplátku za jeho/jej spoločnosť. Partneri sa zvyčajne stretávajú, aby spolu trávili svoj voľný čas a k sexu dochádza len vtedy, ak s tým obaja súhlasia. Ohodnoťte prosím nasledujúce tvrdenia, **akoby ste boli sladkým dievčaťom / chlapcom**, t. j. osobou, ktorá za svoju spoločnosť dostáva materiálne zdroje. Uveďte, do akej miery súhlasíte s každým z nasledujúcich výrokov pomocou 7-bodovej hodnotiacej stupnice od (1) “absolútne nesúhlasím” do (7) “absolútne súhlasím”.

| absolútne nesúhlasím = | 1 | 2 | 3 | 4 | 5 | 6 | 7 | = absolútne súhlasím |
| --- | --- | --- | --- | --- | --- | --- | --- | --- |

| Sladký vzťah je dobrá vec, pretože môže sladkému dievčaťu/chlapcovi pomôcť k uspokojivej finančnej situácii. | \| 1 \| 2 \| 3 \| 4 \| 5 \| 6 \| 7 \| \| --- \| --- \| --- \| --- \| --- \| --- \| --- \| |
| --- | --- | --- | --- | --- | --- | --- | --- | --- |
| V budúcnosti by som mohol/mohla skončiť v sladkom vzťahu. | \| 1 \| 2 \| 3 \| 4 \| 5 \| 6 \| 7 \| \| --- \| --- \| --- \| --- \| --- \| --- \| --- \| |
| Rád/Rada by som vyskúšal/a sladký vzťah, keby som vedel/a, že za to nebudem negatívne posudzovaný/á, alebo z toho nebudú plynúť negatívne dôsledky. | \| 1 \| 2 \| 3 \| 4 \| 5 \| 6 \| 7 \| \| --- \| --- \| --- \| --- \| --- \| --- \| --- \| |
| Zvážil/a by som sladký vzťah, keby to pomohlo mojej kariére. | \| 1 \| 2 \| 3 \| 4 \| 5 \| 6 \| 7 \| \| --- \| --- \| --- \| --- \| --- \| --- \| --- \| |
| Vážne by som zvážil/a sladký vzťah, ak by som si myslel/a, že to zlepší moju finančnú situáciu. | \| 1 \| 2 \| 3 \| 4 \| 5 \| 6 \| 7 \| \| --- \| --- \| --- \| --- \| --- \| --- \| --- \| |

Nasledujúce tvrdenia Vám môžu pripadať podobné. Teraz však, prosím, ohodnoťte tieto tvrdenia tak, **akoby ste boli sladkým tatom / maminou**, teda osobou, ktorá poskytuje materiálne zdroje pre svojho partnera. Pomocou 7-bodovej hodnotiacej škály, prosím, uveďte do akej miery súhlasíte s každým, nasledujúcim tvrdením od (1) “absolútne nesúhlasím” to (7) “absolútne súhlasím”.

| absolútne nesúhlasím = | 1 | 2 | 3 | 4 | 5 | 6 | 7 | = absolútne súhlasím |
| --- | --- | --- | --- | --- | --- | --- | --- | --- |

| Sladký vzťah je dobrá vec, pretože môže pomôcť ľuďom cítiť sa lepšie. | \| 1 \| 2 \| 3 \| 4 \| 5 \| 6 \| 7 \| \| --- \| --- \| --- \| --- \| --- \| --- \| --- \| |
| --- | --- | --- | --- | --- | --- | --- | --- | --- |
| V budúcnosti by som mohol/mohla skončiť v sladkom vzťahu. | \| 1 \| 2 \| 3 \| 4 \| 5 \| 6 \| 7 \| \| --- \| --- \| --- \| --- \| --- \| --- \| --- \| |
| Rád/Rada by som vyskúšala sladký vzťah, keby som vedela, že za to nebudem negatívne posudzovaný/á, alebo z toho nebudú plynúť negatívne dôsledky. | \| 1 \| 2 \| 3 \| 4 \| 5 \| 6 \| 7 \| \| --- \| --- \| --- \| --- \| --- \| --- \| --- \| |
| Zvážil/a by som sladký vzťah, keby to bolo prospešné pre môj sexuálny život, alebo prospešné pre vytvorenie mienky o mne. | \| 1 \| 2 \| 3 \| 4 \| 5 \| 6 \| 7 \| \| --- \| --- \| --- \| --- \| --- \| --- \| --- \| |
| Vážne by som zvážil/a sladký vzťah, ak by to bol spôsob, ako nájsť partnera, ktorý by vyhovoval všetkým mojim potrebám. | \| 1 \| 2 \| 3 \| 4 \| 5 \| 6 \| 7 \| \| --- \| --- \| --- \| --- \| --- \| --- \| --- \| |

Language: Slovenian

**Country: Slovenia**

**"Sponzorsko" partnersko razmerje (angl. "sugar relationship")** je transakcijsko spolno razmerje, v katerem starejši/-a in bogatejši/-a partner/-ka (sponzorski očka oz. sponzorska mami; angl. "sugar daddy" in "sugar mommy") v zameno za druženje zagotovi materialna sredstva mlajšemu/-i partnerju/-ki ("sponzoriranemu" dekletu oz. fantu; angl. "sugar baby" in "sugar boy"). Partnerja se navadno sestaneta, da bi skupaj preživela prosti čas, spolna aktivnost pa je vključena le, če oba partnerja v to privolita.  Prosimo, ocenite naslednje izjave, kot da **ste "sponzorirano" dekle oz. fant** , torej oseba, ki za druženje prejema materialna sredstva. Prosimo označite, v kolikšni meri se strinjate z vsako od spodnjih trditev, pri čemer uporabite sedemstopnjsko ocenjevalno lestvico od (1) “sploh se ne strinjam” do (7) “povsem se strinjam”.

| sploh se ne strinjam = | 1 | 2 | 3 | 4 | 5 | 6 | 7 | = povsem se strinjam |
| --- | --- | --- | --- | --- | --- | --- | --- | --- |

| Sponzorsko partnersko razmerje je dobra stvar, saj lahko "sponzoriranemu" dekletu oz. fantu pomaga do zadovoljivega finančnega stanja. | \| 1 \| 2 \| 3 \| 4 \| 5 \| 6 \| 7 \| \| --- \| --- \| --- \| --- \| --- \| --- \| --- \| |
| --- | --- | --- | --- | --- | --- | --- | --- | --- |
| V prihodnosti bi se lahko zapletel/-la v "sponzorsko" partnersko razmerje. | \| 1 \| 2 \| 3 \| 4 \| 5 \| 6 \| 7 \| \| --- \| --- \| --- \| --- \| --- \| --- \| --- \| |
| Če bi vedel/-a, da ne bom deležen/-na negativnega obsojanja ali posledic, bi preizkusil/-a "sponzorsko" partnersko razmerje. | \| 1 \| 2 \| 3 \| 4 \| 5 \| 6 \| 7 \| \| --- \| --- \| --- \| --- \| --- \| --- \| --- \| |
| Če bi to koristilo moji karieri, bi razmišljal/-a o tem, da bi se spustil/-a v "sponzorsko" partnersko razmerje. | \| 1 \| 2 \| 3 \| 4 \| 5 \| 6 \| 7 \| \| --- \| --- \| --- \| --- \| --- \| --- \| --- \| |
| Resno bi razmislil/-a o tem, da bi se spustil/-a v "sponzorsko" partnersko razmerje, če bi mislil/-a, da mi bo to pomagalo do boljšega finančnega stanja. | \| 1 \| 2 \| 3 \| 4 \| 5 \| 6 \| 7 \| \| --- \| --- \| --- \| --- \| --- \| --- \| --- \| |

Naslednje izjave se bodo morda zdele podobne, vendar tokrat ocenite naslednje trditve, kot da **ste "sponzorski" očka/"sponzorska" mami**, torej oseba, ki svojemu/-i partnerju/-ki za druženje nudi materialna sredstva. Prosimo označite, v kolikšni meri se strinjate z vsako od spodnjih trditev, pri čemer uporabite sedemstopnjsko ocenjevalno lestvico od (1) “sploh se ne strinjam” do (7) “povsem se strinjam”.

| sploh se ne strinjam = | 1 | 2 | 3 | 4 | 5 | 6 | 7 | = povsem se strinjam |
| --- | --- | --- | --- | --- | --- | --- | --- | --- |

| Sponzorsko partnersko razmerje je dobra stvar, saj lahko ljudem pomaga, da se počutijo bolje. | \| 1 \| 2 \| 3 \| 4 \| 5 \| 6 \| 7 \| \| --- \| --- \| --- \| --- \| --- \| --- \| --- \| |
| --- | --- | --- | --- | --- | --- | --- | --- | --- |
| V prihodnosti bi se lahko zapletel/-la v "sponzorsko" partnersko razmerje. | \| 1 \| 2 \| 3 \| 4 \| 5 \| 6 \| 7 \| \| --- \| --- \| --- \| --- \| --- \| --- \| --- \| |
| Če bi vedel/-a, da ne bom deležen/-na negativnega obsojanja ali posledic, bi preizkusil/-a "sponzorsko" partnersko razmerje. | \| 1 \| 2 \| 3 \| 4 \| 5 \| 6 \| 7 \| \| --- \| --- \| --- \| --- \| --- \| --- \| --- \| |
| Če bi bilo to koristno za moje spolno življenje ali mnenje drugih o meni, bi razmislil/-a o tem, da bi se spustil/-a v "sponzorsko" partnersko razmerje. | \| 1 \| 2 \| 3 \| 4 \| 5 \| 6 \| 7 \| \| --- \| --- \| --- \| --- \| --- \| --- \| --- \| |
| Resno bi razmislil/-a o tem, da bi se spustil/-a v "sponzorsko" partnersko razmerje, če bi na ta način našel/-la partnerja/-ko, ki bi zadovoljil/-a vse moje potrebe. | \| 1 \| 2 \| 3 \| 4 \| 5 \| 6 \| 7 \| \| --- \| --- \| --- \| --- \| --- \| --- \| --- \| |

Language: Spanish

**Country: Spain**

La **relación de "sugar"** es una relación sexual transaccional en la que una pareja mayor y más rica (sugar mommy/daddy) proporciona recursos materiales a una pareja más joven (bebé/niño de azúcar) a cambio de su compañía. Las parejas generalmente se reúnen para pasar tiempo libre juntos, y la actividad sexual solo se involucra si ambos dan su consentimiento. Califique las siguientes afirmaciones **como si fuera un sugar baby/boy**, es decir, una persona que recibe recursos materiales para su compañía. Indique hasta qué punto está de acuerdo con cada una de ellas utilizando las escalas de calificación de siete puntos que van desde (1) “absolutamente en desacuerdo” a (7) ”absolutamente de acuerdo”.

| absolutamente en desacuerdo = | 1 | 2 | 3 | 4 | 5 | 6 | 7 | = absolutamente de acuerdo |
| --- | --- | --- | --- | --- | --- | --- | --- | --- |

| Una relación "sugar" es algo bueno porque puede ayudar al sugar baby/boy a tener una situación financiera satisfactoria. | \| 1 \| 2 \| 3 \| 4 \| 5 \| 6 \| 7 \| \| --- \| --- \| --- \| --- \| --- \| --- \| --- \| |
| --- | --- | --- | --- | --- | --- | --- | --- | --- |
| En el futuro, podría terminar entablando una relación "sugar" | \| 1 \| 2 \| 3 \| 4 \| 5 \| 6 \| 7 \| \| --- \| --- \| --- \| --- \| --- \| --- \| --- \| |
| Si supiera que no incurriría en juicios ni consecuencias negativas, me gustaría intentar una relación "sugar" | \| 1 \| 2 \| 3 \| 4 \| 5 \| 6 \| 7 \| \| --- \| --- \| --- \| --- \| --- \| --- \| --- \| |
| Si eso beneficiara mi carrera, pensaría en entablar una relación "sugar" | \| 1 \| 2 \| 3 \| 4 \| 5 \| 6 \| 7 \| \| --- \| --- \| --- \| --- \| --- \| --- \| --- \| |
| Consideraría seriamente entablar una relación "sugar" si pensara que me ayudaría a tener una mejor situación financiera. | \| 1 \| 2 \| 3 \| 4 \| 5 \| 6 \| 7 \| \| --- \| --- \| --- \| --- \| --- \| --- \| --- \| |

Las siguientes declaraciones pueden parecer similares. Pero ahora, califique las siguientes afirmaciones **como si fuera un "sugar mommy/daddy"**, es decir, una persona que proporciona recursos materiales para la compañía de su pareja. Indique hasta qué punto está de acuerdo con cada una de ellas utilizando las escalas de calificación de siete puntos que van desde (1) “absolutamente en desacuerdo” hasta (7) “absolutamente de acuerdo”.

| absolutamente en desacuerdo = | 1 | 2 | 3 | 4 | 5 | 6 | 7 | = absolutamente de acuerdo |
| --- | --- | --- | --- | --- | --- | --- | --- | --- |

| Una relación "sugar" es algo bueno porque puede ayudar a las personas a sentirse mejor. | \| 1 \| 2 \| 3 \| 4 \| 5 \| 6 \| 7 \| \| --- \| --- \| --- \| --- \| --- \| --- \| --- \| |
| --- | --- | --- | --- | --- | --- | --- | --- | --- |
| En el futuro, podría terminar entablando una relación "sugar". | \| 1 \| 2 \| 3 \| 4 \| 5 \| 6 \| 7 \| \| --- \| --- \| --- \| --- \| --- \| --- \| --- \| |
| Si supiera que no incurriría en juicios ni consecuencias negativas, me gustaría intentar una relación "sugar" | \| 1 \| 2 \| 3 \| 4 \| 5 \| 6 \| 7 \| \| --- \| --- \| --- \| --- \| --- \| --- \| --- \| |
| Si fuera beneficioso para mi vida sexual o para el juicio de otros sobre mí, consideraría entablar una relación "sugar". | \| 1 \| 2 \| 3 \| 4 \| 5 \| 6 \| 7 \| \| --- \| --- \| --- \| --- \| --- \| --- \| --- \| |
| Consideraría seriamente entablar una relación "sugar" si esa fuera la forma de encontrar una pareja que satisfaga todas mis necesidades. | \| 1 \| 2 \| 3 \| 4 \| 5 \| 6 \| 7 \| \| --- \| --- \| --- \| --- \| --- \| --- \| --- \| |

Language: Spanish (Latin American)

**Country: Argentina, Bolivia, Chile, Colombia, Dominican Republic, Ecuador, El Salvador, Guatemala, Honduras, Mexico, Peru, Uruguay, and Venezuela.**

La **relación de "sugar"** es una relación sexual transaccional en la que una pareja mayor y más rica (sugar mommy/daddy) proporciona recursos materiales a una pareja más joven (bebé/niño de azúcar) a cambio de su compañía. Las parejas generalmente se reúnen para pasar tiempo libre juntos, y la actividad sexual solo se involucra si ambos dan su consentimiento. Califique las siguientes afirmaciones **como si** **fuera un sugar baby/boy**, es decir, una persona que recibe recursos materiales para su compañía. Indique hasta qué punto está de acuerdo con cada una de ellas utilizando las escalas de calificación de siete puntos que van desde (1)  “absolutamente en desacuerdo” a (7) “totalmente de acuerdo”.

| absolutamente en desacuerdo = | 1 | 2 | 3 | 4 | 5 | 6 | 7 | = absolutamente de acuerdo |
| --- | --- | --- | --- | --- | --- | --- | --- | --- |

| Una relación "sugar" es algo bueno porque puede ayudar al sugar baby/boy a tener una situación financiera satisfactoria. | \| 1 \| 2 \| 3 \| 4 \| 5 \| 6 \| 7 \| \| --- \| --- \| --- \| --- \| --- \| --- \| --- \| |
| --- | --- | --- | --- | --- | --- | --- | --- | --- |
| En el futuro, podría terminar entablando una relación "sugar" | \| 1 \| 2 \| 3 \| 4 \| 5 \| 6 \| 7 \| \| --- \| --- \| --- \| --- \| --- \| --- \| --- \| |
| Si supiera que no incurriría en juicios ni consecuencias negativas, me gustaría intentar una relación "sugar" | \| 1 \| 2 \| 3 \| 4 \| 5 \| 6 \| 7 \| \| --- \| --- \| --- \| --- \| --- \| --- \| --- \| |
| Si eso beneficiara mi carrera, pensaría en entablar una relación "sugar" | \| 1 \| 2 \| 3 \| 4 \| 5 \| 6 \| 7 \| \| --- \| --- \| --- \| --- \| --- \| --- \| --- \| |
| Consideraría seriamente entablar una relación "sugar" si pensara que me ayudaría a tener una mejor situación financiera. | \| 1 \| 2 \| 3 \| 4 \| 5 \| 6 \| 7 \| \| --- \| --- \| --- \| --- \| --- \| --- \| --- \| |

Las siguientes declaraciones pueden parecer similares. Pero ahora, califique las siguientes afirmaciones **como si fuera un "sugar mommy/daddy"**, es decir, una persona que proporciona recursos materiales para la compañía de su pareja. Indique hasta qué punto está de acuerdo con cada una de ellas utilizando las escalas de calificación de siete puntos que van desde (1) “absolutamente en desacuerdo” hasta (7) “absolutamente de acuerdo”.

| absolutamente en desacuerdo = | 1 | 2 | 3 | 4 | 5 | 6 | 7 | = absolutamente de acuerdo |
| --- | --- | --- | --- | --- | --- | --- | --- | --- |

| Una relación "sugar" es algo bueno porque puede ayuda a las personas a sentirse mejor. | \| 1 \| 2 \| 3 \| 4 \| 5 \| 6 \| 7 \| \| --- \| --- \| --- \| --- \| --- \| --- \| --- \| |
| --- | --- | --- | --- | --- | --- | --- | --- | --- |
| En el futuro, podría terminar entablando una relación "sugar". | \| 1 \| 2 \| 3 \| 4 \| 5 \| 6 \| 7 \| \| --- \| --- \| --- \| --- \| --- \| --- \| --- \| |
| Si supiera que no incurriría en juicios ni consecuencias negativas, me gustaría intentar una relación "sugar" | \| 1 \| 2 \| 3 \| 4 \| 5 \| 6 \| 7 \| \| --- \| --- \| --- \| --- \| --- \| --- \| --- \| |
| Si fuera beneficioso para mi vida sexual o para el juicio de otros sobre mí, consideraría entablar una relación "sugar". | \| 1 \| 2 \| 3 \| 4 \| 5 \| 6 \| 7 \| \| --- \| --- \| --- \| --- \| --- \| --- \| --- \| |
| Consideraría seriamente entablar una relación "sugar" si esa fuera la forma de encontrar una pareja que satisfaga todas mis necesidades. | \| 1 \| 2 \| 3 \| 4 \| 5 \| 6 \| 7 \| \| --- \| --- \| --- \| --- \| --- \| --- \| --- \| |

Language: Swedish

**Country: Sweden**

Ett **sugarförhållande** är ett transaktionellt sexuellt förhållande där en äldre och rikare partner (sugar daddy/mommy) ger materiella resurser till en yngre partner (sugar baby/boy) i utbyte mot hennes eller hans sällskap. Partners träffas vanligtvis för att tillbringa tid tillsammans, och sexuell aktivitet är endast inblandad om båda parter ger sitt samtycke. Vänligen betygsätt följande påståenden som om **du vore en sugar baby/boy**, dvs. en person som får materiella resurser för sitt sällskap. Vänligen ange i vilken utsträckning du håller med om vart och ett av nedanstående påståenden på en sju-punkts betygsskala från (1) “håller absolut inte med” till (7) “håller absolut med”.

| håller absolut inte med = | 1 | 2 | 3 | 4 | 5 | 6 | 7 | = håller absolut med |
| --- | --- | --- | --- | --- | --- | --- | --- | --- |

| Ett sugarförhållande är någonting bra eftersom det kan hjälpa sugarbabyn/boy att ha en tillfredsställande ekonomisk situation. | \| 1 \| 2 \| 3 \| 4 \| 5 \| 6 \| 7 \| \| --- \| --- \| --- \| --- \| --- \| --- \| --- \| |
| --- | --- | --- | --- | --- | --- | --- | --- | --- |
| I framtiden skulle jag kunna ha ett sugarförhållande. | \| 1 \| 2 \| 3 \| 4 \| 5 \| 6 \| 7 \| \| --- \| --- \| --- \| --- \| --- \| --- \| --- \| |
| Om jag visste att jag inte skulle bli negativt bedömd eller få negativa konsekvenser skulle jag vilja prova ett sugarförhållande. | \| 1 \| 2 \| 3 \| 4 \| 5 \| 6 \| 7 \| \| --- \| --- \| --- \| --- \| --- \| --- \| --- \| |
| Om det skulle gynna min karriär skulle jag överväga att ha ett sugarförhållande. | \| 1 \| 2 \| 3 \| 4 \| 5 \| 6 \| 7 \| \| --- \| --- \| --- \| --- \| --- \| --- \| --- \| |
| Jag skulle allvarligt överväga att ha ett sugarförhållande om jag trodde att det skulle hjälpa mig att få en bättre ekonomisk situation. | \| 1 \| 2 \| 3 \| 4 \| 5 \| 6 \| 7 \| \| --- \| --- \| --- \| --- \| --- \| --- \| --- \| |

Följande påståenden kan verka lika. Men betygsätt vänligen följande påståenden som om **du vore en sugar daddy/mommy**, dvs. en person som bistår materiella resurser för en partners sällskap. Vänligen ange i vilken utsträckning du håller med om vart och ett av nedanstående påståenden på en sju-punkts betygsskala från (1) “håller absolut inte med” to (7) “håller absolut med”.

| håller absolut inte med = | 1 | 2 | 3 | 4 | 5 | 6 | 7 | = håller absolut med |
| --- | --- | --- | --- | --- | --- | --- | --- | --- |

| Ett sugarförhållande är bra eftersom det kan hjälpa människor att må bättre. | \| 1 \| 2 \| 3 \| 4 \| 5 \| 6 \| 7 \| \| --- \| --- \| --- \| --- \| --- \| --- \| --- \| |
| --- | --- | --- | --- | --- | --- | --- | --- | --- |
| I framtiden skulle jag kunna ha ett sugarförhållande. | \| 1 \| 2 \| 3 \| 4 \| 5 \| 6 \| 7 \| \| --- \| --- \| --- \| --- \| --- \| --- \| --- \| |
| Om jag visste att jag inte skulle bli negativt bedömd eller få negativa konsekvenser skulle jag vilja prova ett sugarförhållande. | \| 1 \| 2 \| 3 \| 4 \| 5 \| 6 \| 7 \| \| --- \| --- \| --- \| --- \| --- \| --- \| --- \| |
| Om det skulle gynna mitt sexliv eller andras bedömning av mig, skulle jag överväga att ha ett sugarförhållande. | \| 1 \| 2 \| 3 \| 4 \| 5 \| 6 \| 7 \| \| --- \| --- \| --- \| --- \| --- \| --- \| --- \| |
| Jag skulle allvarligt överväga att ha ett sugarförhållande om det var sättet att hitta en partner som skulle tillgodose alla mina behov. | \| 1 \| 2 \| 3 \| 4 \| 5 \| 6 \| 7 \| \| --- \| --- \| --- \| --- \| --- \| --- \| --- \| |

Language: Turkish

**Country: Türkiye, Azerbaijan**

Bir **şeker ilişkisi**, daha yaşlı ve daha zengin bir partnerin (şeker baba/şeker anne) genç bir partnere (şeker kız/şeker oğlan) eşliği karşılığında maddi kaynaklar sağladığı bir cinsel ilişkidir. Partnerler genellikle birlikte zaman geçirmek için bir araya gelirler ve cinsel aktivite sadece her iki tarafın da rıza göstermesi durumunda gerçekleşir. Lütfen aşağıdaki ifadeleri kendinizi **bir şeker kız/şeker oğlanmış gibi düşünerek**, yani eşliği karşılığında maddi kaynak elde eden bir kişi gibi değerlendirin. Lütfen aşağıdaki ifadelerin her birine ne ölçüde katıldığınızı, (1) “kesinlikle katılmıyorum” ile (7) “kesinlikle katılıyorum” arasında değişen yedili derecelendirme ölçeği kullanarak, belirtiniz.

| kesinlikle katılmıyorum = | 1 | 2 | 3 | 4 | 5 | 6 | 7 | = kesinlikle katılıyorum |
| --- | --- | --- | --- | --- | --- | --- | --- | --- |

| Bence şeker ilişkisi iyi bir şeydir, çünkü şeker kız/oğlanın tatmin edici bir maddi duruma sahip olmasına yardımcı olabilir. | \| 1 \| 2 \| 3 \| 4 \| 5 \| 6 \| 7 \| \| --- \| --- \| --- \| --- \| --- \| --- \| --- \| |
| --- | --- | --- | --- | --- | --- | --- | --- | --- |
| Gelecekte bir şeker ilişkisine girebilirim. | \| 1 \| 2 \| 3 \| 4 \| 5 \| 6 \| 7 \| \| --- \| --- \| --- \| --- \| --- \| --- \| --- \| |
| Olumsuz yargılara veya bedellere maruz kalmayacağımı bilseydim, bir şeker ilişkisini denemek isterdim. | \| 1 \| 2 \| 3 \| 4 \| 5 \| 6 \| 7 \| \| --- \| --- \| --- \| --- \| --- \| --- \| --- \| |
| Kariyerime bir fayda sağlayacaksa, bir şeker ilişkisine girmeyi düşünürdüm. | \| 1 \| 2 \| 3 \| 4 \| 5 \| 6 \| 7 \| \| --- \| --- \| --- \| --- \| --- \| --- \| --- \| |
| Daha iyi bir maddi duruma sahip olmama yardımcı olacağını düşünseydim, şeker ilişkisine girmeyi ciddi olarak değerlendirirdim. | \| 1 \| 2 \| 3 \| 4 \| 5 \| 6 \| 7 \| \| --- \| --- \| --- \| --- \| --- \| --- \| --- \| |

Aşağıdaki ifadeler öncekilere benzer görünebilir. Fakat şimdi, lütfen aşağıdaki ifadeleri kendinizi bir **şeker baba/anneymiş gibi düşünerek**, yani eşliği için partnerine maddi kaynaklar sağlayan bir kişi gibi değerlendirin. Lütfen aşağıdaki ifadelerin her birine ne ölçüde katıldığınızı, (1) “kesinlikle katılmıyorum” ile (7) “kesinlikle katılıyorum” arasında değişen yedili derecelendirme ölçeği kullanarak, belirtiniz.

| kesinlikle katılmıyorum = | 1 | 2 | 3 | 4 | 5 | 6 | 7 | = kesinlikle katılıyorum |
| --- | --- | --- | --- | --- | --- | --- | --- | --- |

| Şeker ilişkisi iyi bir şeydir, çünkü insanların kendilerini daha iyi hissetmelerine yardımcı olabilir. | \| 1 \| 2 \| 3 \| 4 \| 5 \| 6 \| 7 \| \| --- \| --- \| --- \| --- \| --- \| --- \| --- \| |
| --- | --- | --- | --- | --- | --- | --- | --- | --- |
| Gelecekte bir şeker ilişkisine girebilirim. | \| 1 \| 2 \| 3 \| 4 \| 5 \| 6 \| 7 \| \| --- \| --- \| --- \| --- \| --- \| --- \| --- \| |
| Olumsuz yargılara veya bedellere maruz kalmayacağımı bilseydim, bir şeker ilişkisini denemek isterdim. | \| 1 \| 2 \| 3 \| 4 \| 5 \| 6 \| 7 \| \| --- \| --- \| --- \| --- \| --- \| --- \| --- \| |
| Cinsel hayatıma veya başkalarının benim hakkımdaki değerlendirmelerine faydası olacaksa, şeker ilişkisine girmeyi düşünürdüm. | \| 1 \| 2 \| 3 \| 4 \| 5 \| 6 \| 7 \| \| --- \| --- \| --- \| --- \| --- \| --- \| --- \| |
| Tüm ihtiyaçlarımı karşılayacak bir partner bulmanın yolu bu olsaydı, bir şeker ilişkisine girmeyi ciddi olarak değerlendirirdim. | \| 1 \| 2 \| 3 \| 4 \| 5 \| 6 \| 7 \| \| --- \| --- \| --- \| --- \| --- \| --- \| --- \| |

Language: Ukrainian

**Country: Ukraine**

**Цукрові стосунки** - це трансакційні сексуальні стосунки, в яких старший та заможніший партнер (цукровий тато / мама) надає матеріальні ресурси молодшому партнеру (цукровій дівчинці / хлопчику) в обмін на її або його товариство. Партнери зазвичай зустрічаються, щоб провести дозвілля разом, а сексуальна активність задіяна лише за умови згоди обох партнерів. Будь ласка, оцініть наступні твердження так, ніби **Ви цукрова дівчинка / хлопчик**, тобто людина, яка отримує матеріальні ресурси за своє товариство. Будь ласка, вкажіть, наскільки Ви погоджуєтесь із кожним із наведених нижче тверджень, використовуючи семибальну шкалу оцінок, що варіюється від (1) “абсолютно не погоджуюсь” до (7) “абсолютно погоджуюсь”.

| абсолютно не погоджуюсь = | 1 | 2 | 3 | 4 | 5 | 6 | 7 | = абсолютно погоджуюсь |
| --- | --- | --- | --- | --- | --- | --- | --- | --- |

| Цукрові стосунки - це добре, оскільки вони можуть допомогти цукровій дівчинці / хлопчику мати бажаний матеріальний стан. | \| 1 \| 2 \| 3 \| 4 \| 5 \| 6 \| 7 \| \| --- \| --- \| --- \| --- \| --- \| --- \| --- \| |
| --- | --- | --- | --- | --- | --- | --- | --- | --- |
| В майбутньому, я міг (могла) би брати участь у цукрових стосунках. | \| 1 \| 2 \| 3 \| 4 \| 5 \| 6 \| 7 \| \| --- \| --- \| --- \| --- \| --- \| --- \| --- \| |
| Якби я знав (-ла), що не зазнаю осуду чи негативних наслідків, я хотів (-ла) би спробувати цукрові стосунки. | \| 1 \| 2 \| 3 \| 4 \| 5 \| 6 \| 7 \| \| --- \| --- \| --- \| --- \| --- \| --- \| --- \| |
| Якби це пішло на користь моїй кар'єрі, я б подумав (-ла) про те, щоб вступити у цукрові стосунки. | \| 1 \| 2 \| 3 \| 4 \| 5 \| 6 \| 7 \| \| --- \| --- \| --- \| --- \| --- \| --- \| --- \| |
| Я б серйозно задумався (-лася) про цукрові стосунки, якби думав (-ла), що це допоможе мені покращити фінансове становище. | \| 1 \| 2 \| 3 \| 4 \| 5 \| 6 \| 7 \| \| --- \| --- \| --- \| --- \| --- \| --- \| --- \| |

Наступні твердження можуть здатися схожими. Але тепер, будь ласка, оцініть наступні твердження так, ніби **Ви цукровий тато / мама**, тобто людина, яка надає матеріальні ресурси за спілкування зі своїм партнером’. Будь ласка, вкажіть, наскільки Ви погоджуєтесь із кожним із наведених нижче тверджень, використовуючи семибальну шкалу оцінок, що варіюється від (1) “абсолютно не погоджуюсь” до (7) “абсолютно погоджуюсь”.

| абсолютно не погоджуюсь = | 1 | 2 | 3 | 4 | 5 | 6 | 7 | = абсолютно погоджуюсь |
| --- | --- | --- | --- | --- | --- | --- | --- | --- |

| Цукрові стосунки - це добре, тому що це може допомогти людям почуватися краще. | \| 1 \| 2 \| 3 \| 4 \| 5 \| 6 \| 7 \| \| --- \| --- \| --- \| --- \| --- \| --- \| --- \| |
| --- | --- | --- | --- | --- | --- | --- | --- | --- |
| В майбутньому, я міг (могла) би брати участь у цукрових стосунках. | \| 1 \| 2 \| 3 \| 4 \| 5 \| 6 \| 7 \| \| --- \| --- \| --- \| --- \| --- \| --- \| --- \| |
| Якби я знав (-ла), що не зазнаю осуду чи негативних наслідків, я хотів (-ла) би спробувати цукрові стосунки. | \| 1 \| 2 \| 3 \| 4 \| 5 \| 6 \| 7 \| \| --- \| --- \| --- \| --- \| --- \| --- \| --- \| |
| Якщо це було б корисно для мого сексуального життя або для формування думки інших про мене, я б подумав (-ла) про те, щоб вступити у цукрові стосунки. | \| 1 \| 2 \| 3 \| 4 \| 5 \| 6 \| 7 \| \| --- \| --- \| --- \| --- \| --- \| --- \| --- \| |
| Я б серйозно подумав (-ла) про те, щоб вступити в цукрові стосунки, якби це був спосіб знайти партнера, який би задовольнив усі мої потреби. | \| 1 \| 2 \| 3 \| 4 \| 5 \| 6 \| 7 \| \| --- \| --- \| --- \| --- \| --- \| --- \| --- \| |
